# Navigating Unexplored Territories of the Interrupted Ugi and Passerini Reactions toward Peptidomimetics

**DOI:** 10.1021/acs.orglett.4c04810

**Published:** 2025-02-17

**Authors:** Paraskevi-Kleio Anastasiou, Michael Fragkiadakis, Maria Thomaidi, Konstantinos G. Froudas, Constantinos G. Neochoritis

**Affiliations:** Department of Chemistry, University of Crete, Heraklion 70013, Greece

## Abstract

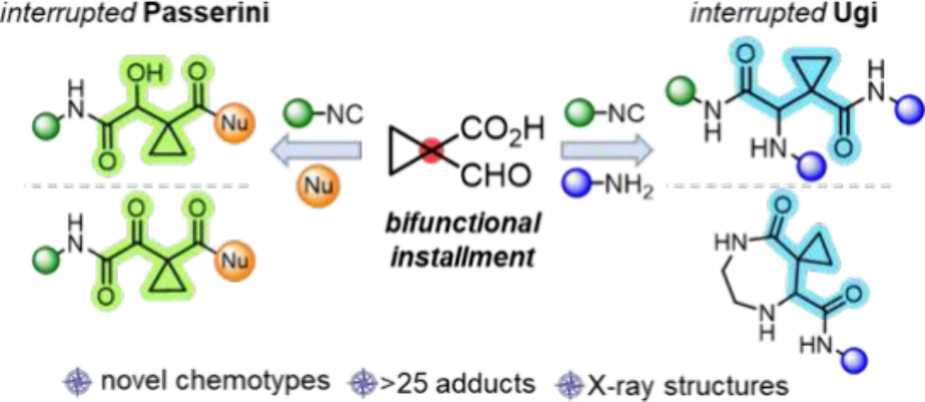

Interrupted reactions redirect established processes, often resulting in unexpected and novel outcomes. By employing a building block containing both acidic and oxo functionalities tethered to the same carbon, we uncovered interrupted variants of the Ugi and Passerini reactions. More than 20 derivatives with a peptide-like framework have been synthesized, demonstrating the broad scope and versatility of these reactions. Additional studies explored the use of various nucleophiles and postmodifications to expand even more the chemical diversity.

Over a century after the discovery of the Passerini reaction (P-3CR)^1,2^ and 60 years since the classical Ugi reaction (U-4CR),^3,4^ these two isocyanide-based multicomponent reactions (IMCRs) continue to hold a prominent position in the modern organic chemist’s toolkit.^5−9^ Moreover, they remain an active area of exploration, offering a fertile ground for new discoveries related to their mechanisms and reactive intermediates.^5,10^

A common mechanistic step in the Ugi and Passerini reactions is the formation of nitrilium ions **1** and **4**, respectively.^11^ The investigation of these intermediates has revealed new synthetic options, shedding light on the interrupted reaction pathways. The potential of the interrupted Ugi and Passerini reactions has been highlighted multiple times, particularly for their ability to selectively intercept this highly reactive nitrilium ion intermediate (Scheme 1, A).^12,13^ In the U-4CR reaction, by replacing the carboxylic acid component with another nucleophile, intermediates can be formed that still undergo a Mumm-type rearrangement.^12^ Alternatively, nucleophiles typically attached to one of the other three components (amine, aldehyde, or isocyanide) can intramolecularly trap the nitrilium ion intermediate without triggering a Mumm rearrangement.^12^ Such pathways offer strategies for engineering enhancements to established MCRs and expand their scope. Some well-known variations include the Ugi Tetrazole (UT-4CR, **8**),^14^ Ugi-Smiles (US-4CR),^15^ Ugi-(thio)hydantoin (UH-4CR),^16^ Ugi-split (**9**),^17^ and Groebke–Blackburn–Bienayme reactions (GBB-3CR, **14**)^18^ (see the Supporting Information, SI). In certain cases, a nucleophilic addition by the solvent plays an interruptive role,^19^ as illustrated by Ugi et al.^20^ in his seminal Ugi 5-center-4-component reaction (U5C-4CR) and by Dömling et al. (**12**),^21^ who utilized this approach to produce iminodicarboxamide derivatives. Besides solvents, a variety of nucleophiles have been employed.^22,23^ Ruijter et al.^23^ introduced a novel Passerini-type reaction, incorporating hexafluoroisopropanol as the acid component toward β-amino alcohols **15** (see the SI for illustrated examples). These developments illustrate how even well-established MCRs can be redirected to yield unconventional yet highly valuable synthetic methodologies. Our idea was to employ a building block where both the acidic and oxo components are tethered to the same carbon (Scheme 1, B). We anticipated that incorporating such a bifunctional component in an Ugi three-center, four-component (U3C-4CR) and a Passerini two-center, three-component (P2C-3CR) reaction would yield the highly strained 2-azetidinones^24^ and 2-oxetanones,^25^ respectively. This approach presents an opportunity to intercept the intermediates with a competing nucleophile, discovering a novel chemical space for peptide-type scaffolds **16** and **17**. Aspoxicillin^26^ and various Cathepsin B inhibitors^27^ (PDB IDs: 1QDQ, 3HHI, 3QSD, 8T8N, 2DC8) among others are based on those frameworks (highlighted by blue and green color for the Ugi and Passerini interrupted versions, respectively). We selected 1-formylcyclopropane-1-carboxylic acid (**20**) as a suitable bifunctional building block. This unique molecule features both formyl and carboxylic groups attached to the same C-1 position on a simple cyclopropyl ring, making it a valuable synthetic building block. Following a standard procedure,^28^ it can be efficiently synthesized from inexpensive starting materials via Ti-Claisen condensation (compound **18**, see the SI), initially producing methyl 1-formylcyclopropanecarboxylate (**19**), followed by a hydrolysis reaction under basic conditions to yield the targeted starting material **20**. The reaction is scalable (it is routinely performed in our lab on a 20–40 mmol scale) and straightforward, enabling the easy incorporation of the cyclopropyl ring. Taking into consideration, cyclopropanes with their unique structural motif and presence in commercially available pharmaceutical candidates and drugs^29^ serve as a bioisostere of 1,2-disubstituted aromatic rings.^30^ Moreover, they offer both metabolic stability and bioactivity, whereas the functionalization of strained cyclopropane frameworks represents an important challenge for chemical synthesis.^31^ To verify our assumptions, a U-4CR was conducted in methanol at 55 °C, utilizing benzylamine, benzyl isocyanide, and **20**. To our delight, the reaction yielded adduct **16a**, providing evidence that **20** does not facilitate the Mumm rearrangement. Instead, the intermediate is intercepted by a second molecule of the starting amine, which acts as the nucleophile, and at the same time, the cyclopropyl ring remains untouched (Scheme 2). Next, various conditions were evaluated to identify the optimal reaction parameters (see the SI). The reaction was carried out either under neat conditions (no solvent) or 2,2,2-trifluoroethanol (TFE) and methanol, at room temperature or reflux. All conditions resulted in the formation of the desired products with similar yields. However, refluxing methanol was selected as the solvent due to its compatibility with a broader range of starting materials. Additionally, different equivalents of the amine were tested, revealing that using two equivalents of the amine, i.e., in case of the derivative **16m**, resulted in a 72% yield of the product, compared to a 66% yield with one equivalent. To address the scope and limitations of this novel interrupted U3C-4CR, a library of 14 derivatives was built, in overall yields of 40–72% (Scheme 2). The synthesis utilized a range of amines, including aliphatic amines, benzyl amines, and anilines with diverse substitution patterns. Benzyl, phenyl, and aliphatic isocyanides were also used, demonstrating a broad scope. We were able to determine the crystal structure of derivative **16n**, revealing the formation of an intramolecular hydrogen bond between the proton of the amide and the nitrogen of the secondary amine groups, which stabilizes the 1,4-bisamide framework (Scheme 2).

**Scheme 1:**
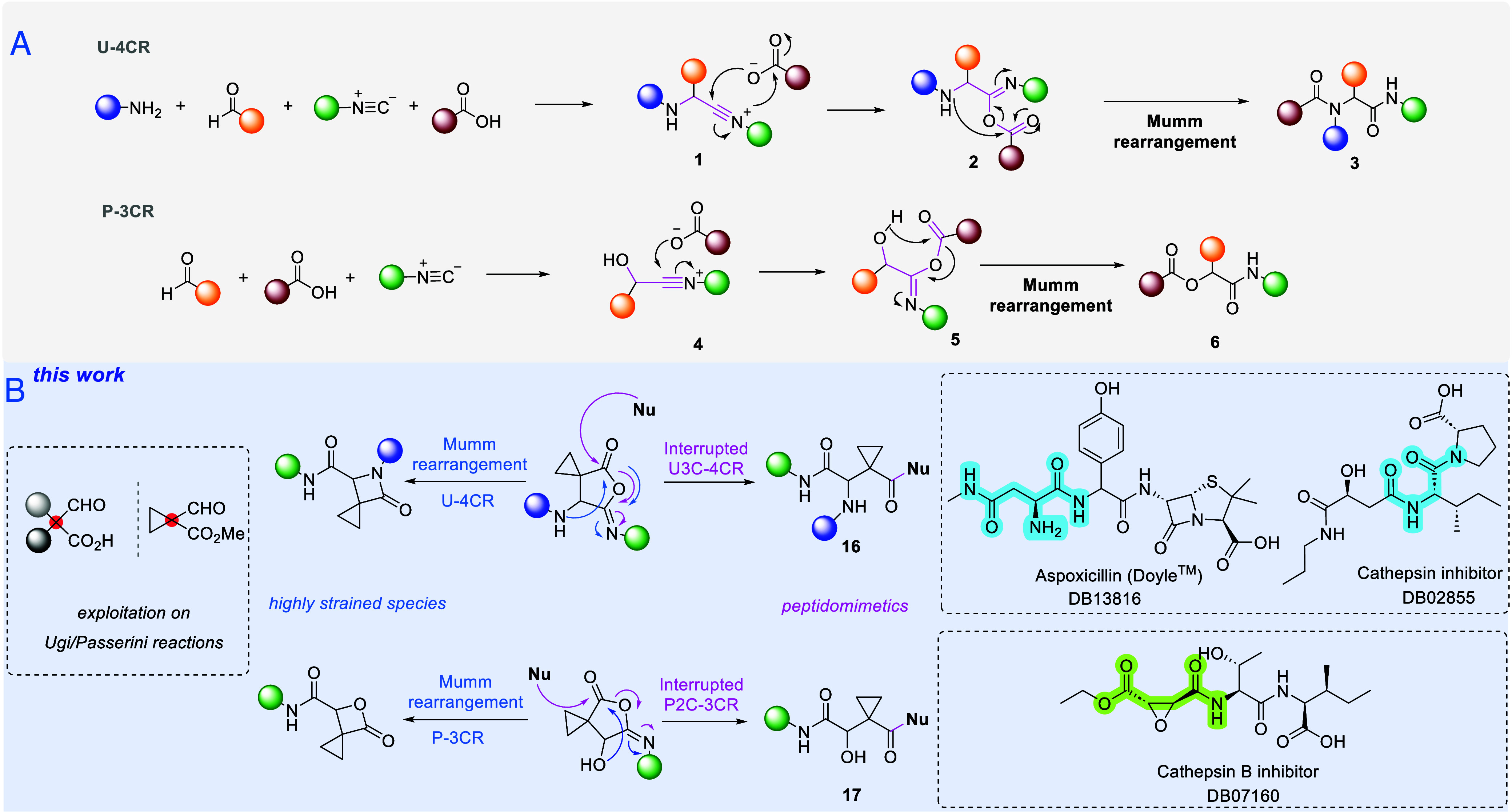
(A) The General Mechanism of the U-4CR and P-3CR Involving the Common Nitrilium Ion; (B) Exploitation of the Interrupted Ugi and Passerini Reactions Based on a Cyclopropyl Building Block Where Both the Acid and Oxo Components Are Tethered to the Same Carbon (Red Highlighted), Affording the Valuable Scaffolds **16** and **17** (Blue and Green Highlighted Frameworks from Bioactive Compounds)

**Scheme 2:**
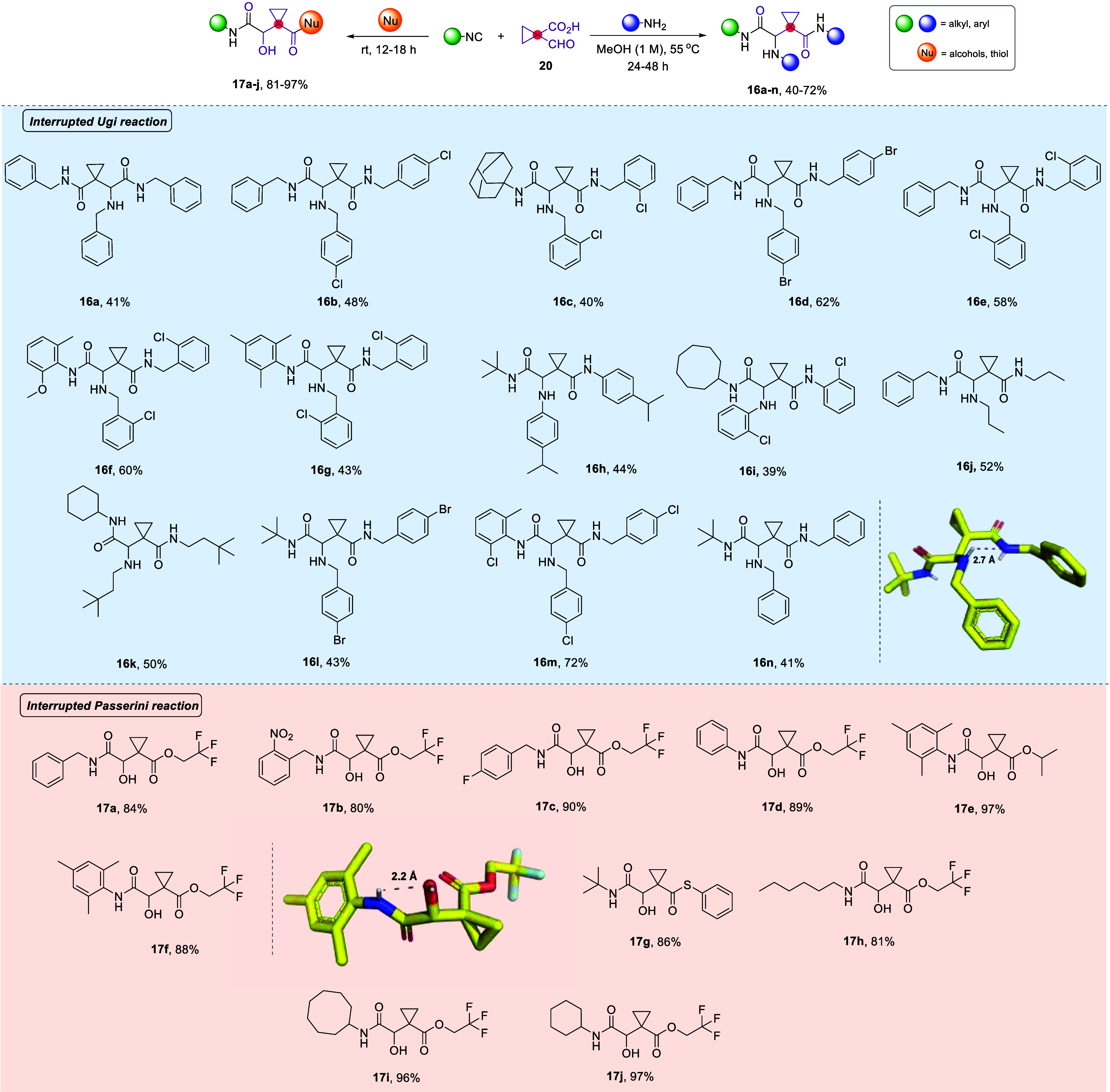
Synthesis of the Key Building Block Which Was Employed in the Interrupted Ugi and Passerini Reactions; the Synthesized Libraries of the U3CR-4C (**16a**–**n**) and P2CR-3C (**17a**–**j**) Single crystal structures of **16n** (CCDC 2412460) and **17f** (CCDC 2412459) were obtained as racemic mixtures (*the hydrogen atoms have been omitted in the crystal structures besides the polar −NH and −OH for clarity reasons*).

In line with this approach, a P2C-3CR was conducted using the same bifunctional building block **20** and an isocyanide in the presence of a nucleophile, which acted in many cases as the solvent (Scheme 2). The Mumm rearrangement was successfully interrupted (see the SI) despite the difficulties that in general the Passerini reaction presents mechanistically,^1,13^ yielding a library of 10 derivatives, in overall yield 81–97%. Different isocyanides have been employed, aliphatic, phenyl, and benzylic ones (Scheme 2), whereas various solvents and reaction conditions were screened to evaluate the reaction’s scope and efficiency regarding the nucleophile (see the SI).

Among these, TFE and isopropanol provided the best results (even at equimolar amounts), delivering the corresponding adduct under mild conditions (55 °C, 12 h) at rt in short reaction times. Solvents with poor nucleophilic properties, such as dichloromethane (DCM), dichloroethane (DCE), and acetonitrile, primarily resulted in unreacted starting materials, even when the temperature and reaction times increased. Also, solvents such as ethanol and methanol led to either unreacted starting materials or traces of the corresponding product with most of the starting material remaining unconverted (see the SI). Expanding the scope of the nucleophile, we have also utilized thiophenol (equimolar), which remarkably even at equimolar amounts under neat conditions yielded the corresponding thioester **17g**.^32^ We were able to solve the crystal structures for compound **17f**, revealing an intramolecular hydrogen bond between the oxygen from the hydroxyl and the −NH– groups, that stabilizes the structure (see the SI for examples of drugs and bioactive compounds with similar scaffolds).

The next question was whether, during the U3CR-4C reaction, preinstalling an attacking additional nucleophile would result in the aforementioned intermolecular interruption (blue arrows) or if the reaction would proceed intramolecularly (orange arrows), leading to the formation of a seven-membered heterocycle (Scheme 3). Thus, when we employed the ethylenediamine, the intramolecular attack from the nitrogen of the diamine (orange arrows) outcompeted the intermolecular attack from the nitrogen of the second molecule of the amine, (blue arrows) yielding compound **18** in 65% yield. On the contrary, when hydroxy propylamine was used, the reaction afforded the intermolecular interrupted version due to the possibly decreased nucleophilicity of the oxygen, affording compound **16o** in 73% yield.

**Scheme 3:**
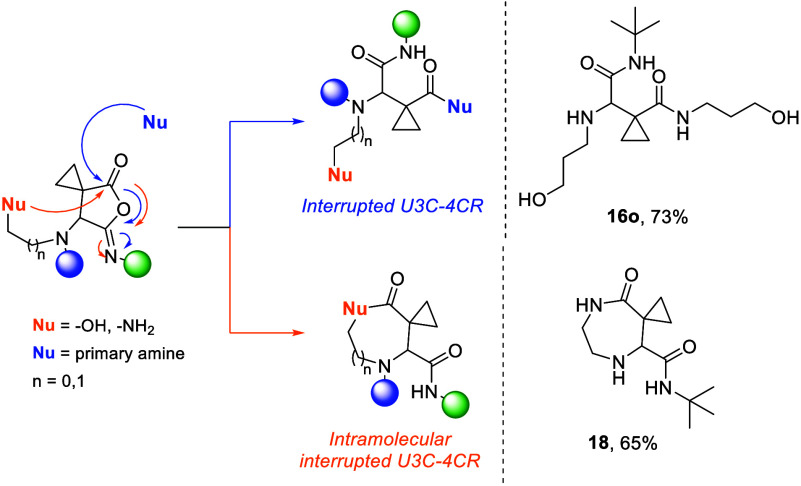
Employment of Bis-Nucleophilic Amines in the Interrupted U3CR-4C Yielding Either the Intramolecularly Cyclized Derivative **18** (Nu = −NH_2_) or the Standard **16o** (Nu = −OH)

Polycarbonyl compounds in which multiple carbonyl groups are directly juxtaposed and influence each other’s chemical reactivity are of great importance and at a certain degree underexplored.^33^ For example, the propensity of tricarbonyl compounds for nucleophilic addition reactions has been recognized, but the applications are strictly limited.^34^ For that reason, we decided to perform a vicinal difunctionalization by oxidizing the hydroxyl group of Passerini adduct **17** (Scheme 4). Therefore, treatment of **17b**–**d**,**f**,**l**,**h**,**j** with pyridinium chlorochromate (PCC) afforded the expected, oxidized tricarbonyl compound **19a**–**d**. Interestingly, when benzyl isocyanides (**17b**,**c**) were employed, the reaction led to the formation of the strained derivative of spiro tricarbonyl imide **21a**,**c** (Scheme 4) alongside the expected product (ratio of conversion **19**:**21** 66%–34%, as indicated by the ^1^H NMR of the reaction mixture). In addition, adducts consisting of the simple phenyl isocyanide (**17d**) were converted 90% to the oxidized and only 10% to the cyclized imide **21d**. Notably, during purification with column chromatography (silica gel), only the cyclic imide was isolated in all cases, regardless of the isocyanide that was employed. This suggests that the formation of these unique tricarbonyl cyclic imides—disclosed here for the first time—is not only highly favored under acidic conditions but also proceeds with remarkable efficiency during workup (see the SI).^35^ In order to verify and establish our theory, we investigated the cyclization conditions, performing a basic treatment with sodium hydride or pyridine of compounds **17** without PCC to determine if the cyclization process could occur independently of the oxidation process.^36^ However, these attempts did not yield the cyclized product. Next, we tested the hypothesis that cyclization might be facilitated by the acidic conditions of silica gel during purification. For that reason, we investigated the reaction under acidic conditions. Derivative **17a** was oxidized and showed conversion of 72% to the oxidized product and 28% to the cyclized product. Upon treatment with *p*TSA the conversion shifted to 50:50. Further treatment with 3 N HCl increased the conversion to 63% cyclized product and 37% oxidized product. Such a result stands in contrast to the general approach, where cyclic imides are traditionally accessed through basic conditions and using anhydrides.^35,37−39^

**Scheme 4:**
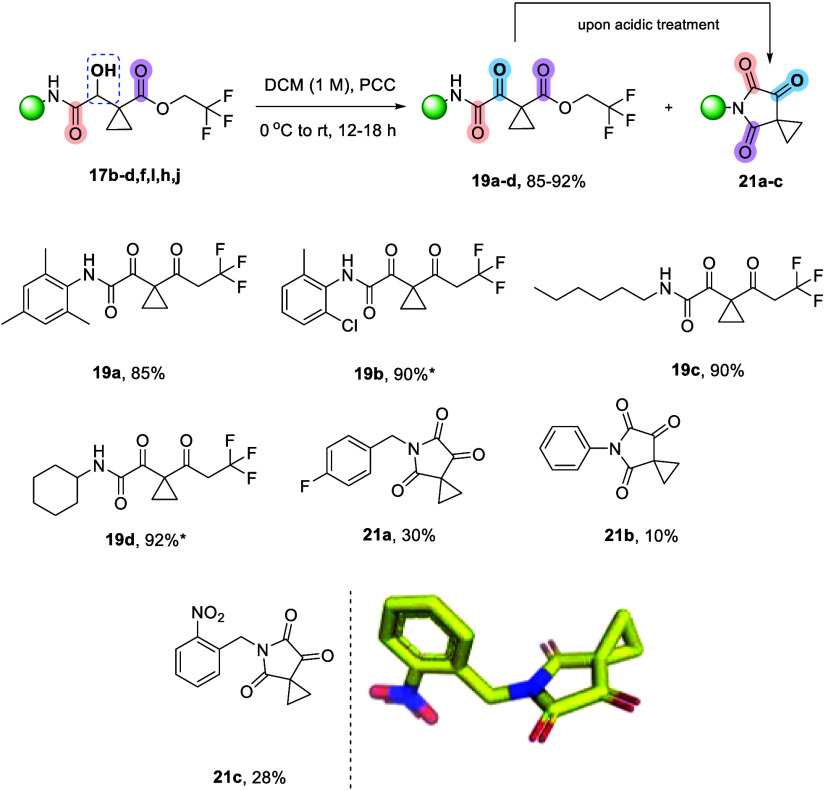
Vicinal Difunctionalization by Oxidizing the Hydroxyl Group of the P2C-3CR Adduct **17** toward the Tricarbonyl Compound **19** and the Cyclic Imide **21** The **17j** toward **19j** was further used without column chromatography. A single crystal structure of **21c** (CCDC 2412461) was obtained. *The corresponding compound***17****was oxidized without purification*.

The realm of interrupted Ugi and Passerini reactions remains largely underexplored. Using a specific cyclopropyl building block that incorporates both acidic and aldehyde functionalities on the same carbon, we hypothesized that an interrupted process would occur instead of forming the highly strained expected spiro-adducts. Consequently, we successfully interrupted both U-4CR and P-3CR, leading to novel chemotypes that form the core framework of various peptide-based bioactive compounds. We synthesized 14 U3C-4CR and 10 P2C-3CR derivatives, demonstrating the broad applicability of these reactions. Furthermore, we expanded the utility of the resulting scaffolds by modifying the functionality of the nucleophilic centers and conducting vicinal difunctionalization through oxidation processes. Single-crystal structures not only confirmed our hypothesis but also provided valuable structural insights and potential binding modes.

## Data Availability

The data underlying this study are available in the published article and its Supporting Information.

## Supporting Information Available

General procedures, characterization data for products (NMR and MS), crystallographic data, and description of mechanisms (PDF)
