# Supplementary material for: Navigating Unexplored Territories of the Interrupted Ugi and Passerini Reactions toward Peptidomimetics
Source: Org Lett. 2025 Feb 17;27(8):1829–34. doi: 10.1021/acs.orglett.4c04810 (PMC11877518; doi:10.1021/acs.orglett.4c04810)
Supplement: Supplementary file 1 — ol4c04810_si_001.pdf [file ol4c04810_si_001.pdf]

Navigating unexplored territories of the interrupted Ugi and Passerini reactions towards peptidomimetics

Paraskevi-Kleio Anastasiou, Michael Fragkiadakis, Maria Thomaidi, Konstantinos G. Froudas, Constantinos G. Neochoritis*

Supporting Information

Table of Contents

1. Experimental materials and methods 2
2. Representative examples of their interrupted versions accessing other chemotypes 3
3. Synthetic procedures and analytical data 4
4. Plausible mechanisms 18
5. Exemplary copies of NMR spectra of novel compounds 19
6. Single crystal x-ray structure determination...54

1. Experimental materials and methods

All the reagents and solvents were purchased from Sigma-Aldrich, AK Scientific, Fluorochem, Abcr GmbH, Acros and were used without further purification. Thin layer chromatography was performed on Millipore precoated silica gel plates (0.20 mm thick, particle size 25 μm). Nuclear magnetic resonance spectra were recorded on Bruker Avance 500 spectrometers {^{1}H NMR (500 MHz), ^{13}C NMR (125 MHz)}. Chemical shifts for ^{1}H NMR were reported as δ values and coupling constants were in hertz (Hz). The following abbreviations were used for spin multiplicity: s = singlet, br s = broad singlet, d = doublet, t = triplet, q = quartet, quin = quintet, dd = double of doublets, dt = double of triplets, td = triplet of doublets, m = multiplet. Chemical shifts for ^{13}C NMR were reported in ppm relative to the solvent peak. High resolution mass spectra were recorded using a LTQ-Orbitrap-XL (Thermo) at a resolution of 60000@m/z400. Single crystal X-ray diffraction data were collected on a Bruker D8 Venture diffractometer equipped with a Cu Incoatec microfocus IμS 3.0 source, a Photon II detector operating in shutterless mode and a cryostream 800 system (Oxford Cryosystems) for temperature regulation. Melting points have been measured in an electrothermal melting point apparatus.

2. Representative examples of their interrupted versions accessing other chemotypes

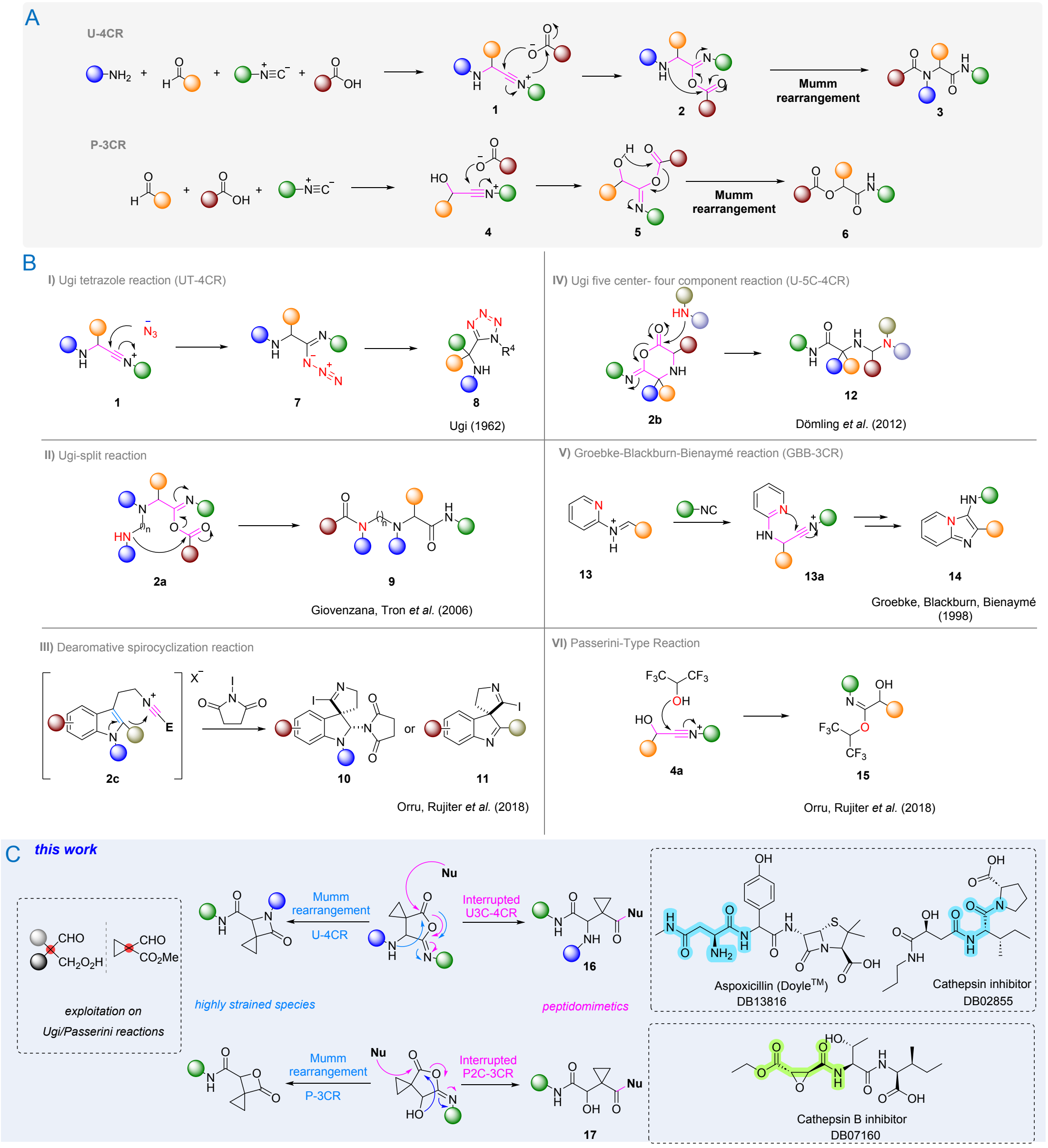

3. Synthetic procedures and analytical data

Synthesis of 1-formylcyclopropane-1-carboxylic acid (20)[1]

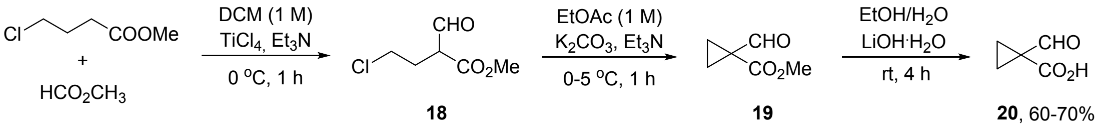

In an oven-dried, two-necked flask, methyl-4-chlorobutyrate (20 mmol, 1 equiv.), methyl formate (60 mmol, 3 equiv.) and DCM (20 mL) were added. The solution was immersed in an ice bath and titanium tetrachloride (44 mmol, 2.2 equiv.), was added dropwise. Then, triethylamine was also added dropwise. The reaction was stirred at 0 ℃ for 1 hour. Then, water (20 mL) was added dropwise into the mixture. The biphasic mixture was transferred in a flask and the initial reaction flask was rinsed with EtOAc (2x1 mL). The mixture was condensed and extractions with EtOAc / H_2O (x2 times) and EtOAc / brine (x2 times) took place. The organic phase was collected, dried over anhydrous sodium sulfate, filtered and condensed. In an oven-dried, two-necked flask, methyl 4-chloro-2-formylbutanoate (24 mmol, 1 equiv.) and EtOAc (24 mL, 1 M) were added. The solution was immersed in an ice bath and potassium carbonate was added into 5 equal parts, over a time of 10 minutes. Then, triethylamine (2.4 mmol, 0.1 equiv.) was added in one portion and the mixture was stirred at 0 ℃ for 2 hours. Then, water was added (24 mL) and extractions with H_2O / EtOAc (x2 times) and brine / EtOAc (x2 times) took place. The organic phase was collected, dried over anhydrous sodium sulfate, filtered and condensed, yielding methyl 1-formylcyclopropane-1-carboxylate.

Lithium hydroxide monohydrate (19.5 mmol, 5 equiv.) was added in H_2O (1 mL) and the solution was stirred, until it was completely dissolved. EtOH (1 mL) and methyl 1-formylcyclopropane-1-carboxylate (3.9 mmol, 1 equiv.) were added, and the reaction was stirred for 4 hours. The mixture was condensed and extraction with DCM / H_2O (1 time) took place. The aqueous phase was quenched with HCl (1 M) until pH~2-3 and extractions with DCM / H_2O (x4 times) took place. The organic phase was collected, dried over anhydrous sodium sulfate, filtered and condensed, yielding the corresponding 1-formylcyclopropane-1-carboxylic acid in 60-70% total yield (3.3 g, 29 mmol).

General procedure for the Ugi three center-four component reaction (U3C-4CR)

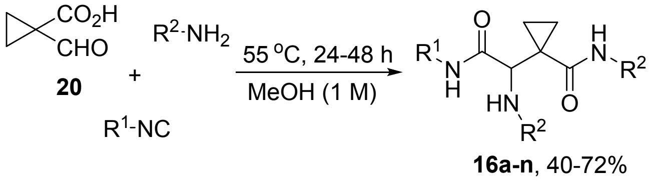

To a stirred solution of 1-formylcyclopropane-1-carboxylic acid (0.2-0.3 mmol, 1 equiv.) in MeOH (1 M), the primary amine (0.2-0.3 mmol, 1 equiv.) was added at room temperature. The reaction mixture was stirred vigorously for 15 min. Then, the isocyanide (0.2-0.3 mmol, 1 equiv.) was added, and the reaction mixture was stirred vigorously at 55 °C for 18 hours on a heating plate. The solvent was removed under

reduced pressure and the reaction mixture was purified with column chromatography (P.E.- EtOAc 7:1-3:1) to yield compounds **16**.

The same experimental procedure was followed for reactions with ethylene diamine and 3-aminopropan-1-ol towards **16o** and **18**.

Optimization of the U3C-4CR

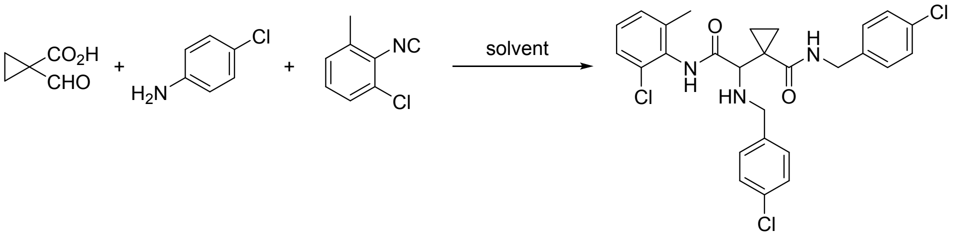

Entry	Solvent	Temperature (°C)	Time (h)	Equiv. (amine)	Yield (%)
**1**	-	25	18	1	30
**2**	TFE	80	18	1	18
**3**	MeOH	25	18	1	32
**4**	MeOH	55	18	1	68
**5**	MeOH	55	18	2	72

General procedure for the Passerini two center-three-component reaction (P2C-3CR)

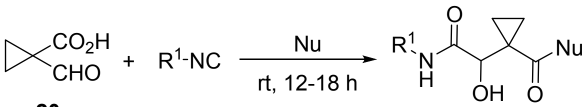

20

17a-k, 81-97%

To a stirred solution of 1-formylcyclopropane-1-carboxylic acid (0.2-0.3 mmol, 1 equiv.) and isocyanide (0.2-0.3 mmol, 1 equiv.), the corresponding nucleophile (1 M) was added. The reaction mixture was stirred at room temperature for 18 hours. The solvent was removed under reduced pressure and the reaction mixture was purified with column chromatography (PE - EtOAc 7:1-3:1) to yield compounds **17**.

Optimization of the P2C-3CR

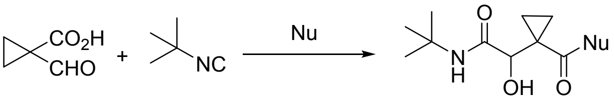

Entry	Solvent	Temperature (°C)	Time (h)	Yield (%)
**1**	TFE	40	48	44
**2**	TFE	25	12-18	50
**3**	MeOH	55	48	N/A
**4**	DCE	60	48	N/A
**5**	ACN	60	48	N/A

General Procedure for the PCC oxidation

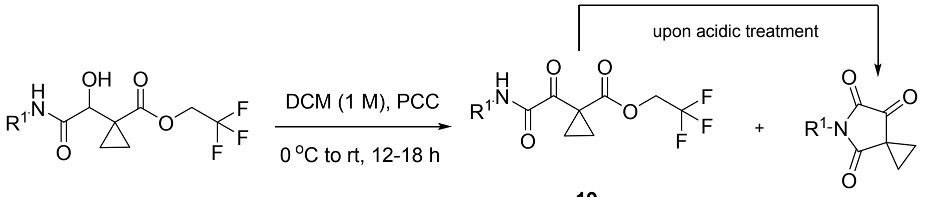

17 **19** **21**

To a stirred solution of compounds **17** (0.05-0.07 mmol, 1 equiv.) in DCM (0.25 M), pyridinium chlorochromate (PCC) was added at 0 ℃. The reaction mixture was stirred at room temperature for 16 - 18 h. Then, Et_2O was added and the reaction mixture was left at -4 ℃ for 1 h. Filtration through SiO_2 with DCM took place. The solvent was removed under reduced pressure and the reaction mixture was purified with column chromatography (PE - EtOAc 7:1-5:1) to yield compounds **19** and **21**.

Optimization of the PCC oxidation

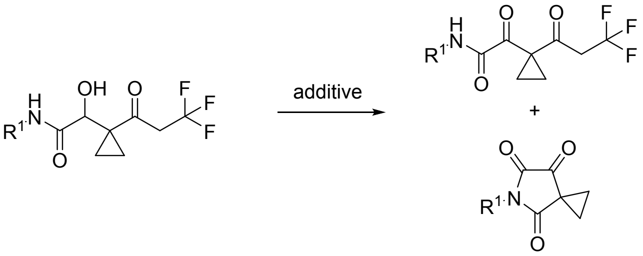

Entry	Solvent	Temperature (°C)	PCC (equiv.)	Additive	Time (h)	Conversion [19(%):21(%)]
**1**	DCM	25	-	Pyridine	12-18	N/A
**2**	DCM	25	-	NaH	12-18	N/A
**3**	DCM	25	1.5	-	12-18	70:30
**4**	DCM	25	1.5	SiO_2	12-18	70:30
**5**	DCM	25	1.5	Pyridine	12-18	70:30
**6**	DCM	25	1.5	*p*TSA	12-18	50:50
**7**	DCM	25	1.5	HCl (3 N)	12-18	40:60

_N_-benzyl-1-(1,2-bis(benzylamino)-2-oxoethyl)cyclopropane-1-carboxamide (16a)

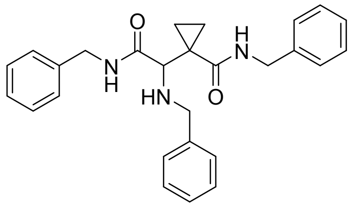

To a stirred solution of 1-formylcyclopropane-1-carboxylic acid (0.114 g, 1.0 mmol, 1 equiv.) in MeOH (1 M), benzylamine (0.107 g, 0.11 ml, 1 mmol, 1 equiv.) was added at room temperature. The reaction mixture was stirred vigorously for 30 min. Then, the benzyl isocyanide (0.117 g, 1 mmol, 1 equiv.) was added, and the reaction mixture was stirred vigorously at 55 °C overnight on a heating plate. The solvent was removed under reduced pressure and the reaction mixture was purified with column chromatography (P.E.- EtOAc 7:1-3:1) to yield compound **16a** (170 mg, 41% yield) as amorphous white solid. ^{1}H NMR (500 MHz, $CDCl_3$): δ 9.31 (s, 1H), 7.34 – 7.27 (m, 7H), 7.25 - 7.21 (m, 7H), 7.12 – 7.10 (m, 2H), 6.76 (t, J = 5.8 Hz, 1H), 4.48 – 4.28 (m, 4H), 4.30 (dd, J_1 = 15.0 Hz, J_2 = 5.0 Hz, 1H), 3.92 (d, *J* = 13.0 Hz, 1H), 3.72 (d, *J* = 13.0 Hz, 1H), 2.93 (s, 1H), 1.52 – 1.48 (m, 1H), 1.28 – 1.24 (m, 1H), 0.82 – 0.78 (m, 1H), 0.73 – 0.69 (m, 1H); ^{13}C NMR (125 MHz, $CDCl_3$): δ 171.9, 170.6, 138.5, 138.5, 137.7, 128.7, 128.6, 128.0, 127.5, 127.5, 127.5, 127.3, 127.2, 64.4, 51.6, 43.7, 43.5, 23.6, 14.4, 10.4; HRMS (ESI) m/z: [M + H]$^+$ Calcd for $C_{27}H_{29}N_3O_2H$ 428.2338; Found 428.2330.

1-(2-(benzylamino)-1-((4-chlorobenzyl)amino)-2-oxoethyl)-_N_-(4-chlorobenzyl) cyclopropane-1-carboxamide (16b)

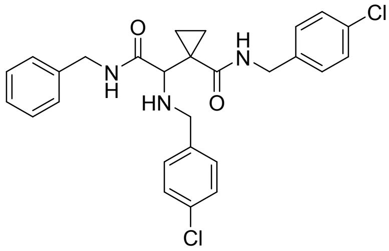

118.3 mg, 48% yield, amorphous white solid. ^{1}H NMR (500 MHz, $CDCl_3$): δ 9.24 (t, *J* = 5.5 Hz, 1H), 7.32 – 7.29 (m, 2H), 7.28 – 7.26 (m, 1H), 7.25 (s, 1H), 7.20 (d, *J* = 8.5 Hz, 3H), 7.12 (d, *J* = 8.0 Hz, 2H), 7.02 (d, *J* = 8.0 Hz, 2H), 6.86 – 6.83 (m, 1H), 4.46 – 4.33 (m, 3H), 4.25 – 4.21 (m, 1H), 3.84 (d, *J* = 13.0 Hz, 1H), 3.69 (d, *J* = 13.0 Hz, 1H), 2.92 (s, 1H), 1.46 – 1.42 (m, 1H), 1.25 – 1.21 (m, 1H), 0.78 – 0.70 (m, 2H); ^{13}C NMR (125 MHz, $CDCl_3$): δ 171.9, 170.4, 137.6, 137.0, 136.9, 133.1, 132.9, 129.3, 128.9, 128.7, 127.5, 127.4, 64.1, 50.7, 43.6, 42.7, 23.6, 14.2, 10.5; HRMS (ESI) m/z: [M + Na]$^+$ Calcd for $C_{27}H_{27}Cl_2N_3O_2Na$ 496.1558; Found 496.1553.

1-(2-(adamantan-1-ylamino)-1-((4-chlorobenzyl)amino)-2-oxoethyl)-_N_-(4-chlorobenzyl)cyclopropanecarboxamide (16c)

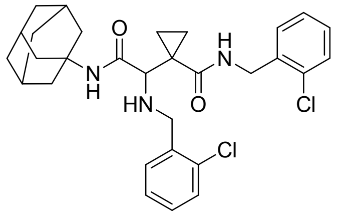

63 mg, 40% yield, amorphous white solid. ^{1}H NMR (500 MHz, $CDCl_3$): δ 9.40 (t, *J* = 6.0 Hz, 1H), 7.35 – 7.33 (m, 1H), 7.31 – 7.30 (m, 1H), 7.28 – 7.25 (m, 2H), 7.20 – 7.18 (m, 2H), 7.17 – 7.14 (m, 2H), 6.79 (d, *J* = 8.5 Hz, 1H), 4.54 – 4.49 (m, 1H), 4.44 – 4.40 (m, 1H), 4.04 (d, *J* = 13.5 Hz, 1H), 4.00 – 3.98 (m, 1H), 3.90 (d, *J* = 13.5 Hz, 1H), 2.90 (s, 1H), 1.85 – 1.81 (m, 8H), 1.71 – 1.59 (m, 8H), 1.52 – 1.49 (m, 1H), 1.31 – 1.25 (m, 1H), 0.91 – 0.87 (m, 1H), 0.74 – 0.70 (m, 1H); ^{13}C NMR (125 MHz, $CDCl_3$): δ 171.7, 169.2, 136.0, 133.9, 133.5, 129.8, 129.7, 129.5, 129.3, 128.7, 128.4, 126.9, 126.7, 64.9, 53.6, 49.3, 41.3, 37.4, 37.0, 36.9, 32.0, 31.9, 31.8, 31.7, 27.0, 27.0, 23.8, 14.5, 10.2; HRMS (ESI) m/z: $[M + H]^+$ Calcd for $C_{30}H_{35}Cl_2N_3O_2H$ 540.2184; Found 540.2182.

1-(2-(benzylamino)-1-((4-bromobenzyl)amino)-2-oxoethyl)-*N*-(4-bromobenzyl) cyclopropane-1-carboxamide (16d)

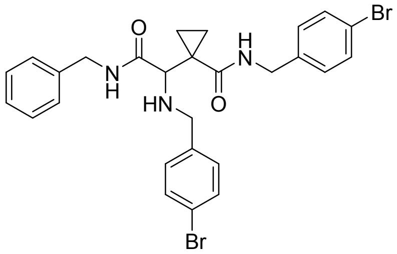

102 mg, 62% yield, amorphous white solid. ^{1}H NMR (500 MHz, $CDCl_3$): δ 9.25 – 9.22 (m, 1H), 7.41 (d, *J* = 8.0 Hz, 2H), 7.36 (d, *J* = 8.0 Hz, 2H), 7.33 – 7.27 (m, 3H), 7.22 – 7.19 (m, 2H), 7.06 (d, *J* = 8.5 Hz, 2H), 6.96 (d, *J* = 8.0 Hz, 2H), 6.75 (t, *J* = 6.0 Hz, 1H), 4.47 – 4.34 (m, 3H), 4.23 – 4.19 (m, 1H), 3.85 (d, *J* = 13.0 Hz, 1H), 3.68 (d, *J* = 13.0 Hz, 1H), 3.02 (br, 1H), 2.89 (s, 1H), 1.50 - 1.46 (m, 1H), 1.26 – 1.22 (m, 1H), 0.79 – 0.75 (m, 1H), 0.72 – 0.69 (m, 1H); ^{13}C NMR (125 MHz, $CDCl_3$): δ 171.7, 170.5, 137.6, 137.6, 137.4, 131.7, 131.7, 129.6, 129.3, 128.8, 127.7, 127.4, 121.3, 121.1, 64.4, 50.8, 43.8, 42.9, 23.5, 14.5, 10.5; HRMS (ESI) m/z: $[M + H]^+$ Calcd for $C_{27}H_{27}Br_2N_3O_2H$ 584.0548; Found 584.0550.

1-(2-(benzylamino)-1-((2-chlorobenzyl)amino)-2-oxoethyl)-*N*-(3-chlorobenzyl) cyclopropane-1-carboxamide (16e)

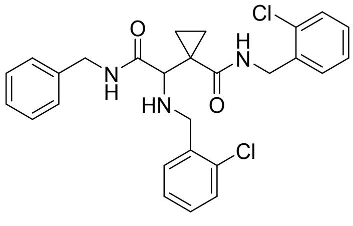

101 mg, 58% yield, amorphous white solid. ^{1}H NMR (500 MHz, $CDCl_3$): δ 9.20 (t, *J* = 5.8 Hz, 1H), 7.35 – 7.28 (m, 5H), 7.25 – 7.16 (m, 8H), 6.72 (t, *J* = 5.8 Hz, 1H), 4.48 – 4.43 (m, 3H), 4.39 – 4.35 (m, 1H), 4.00 (d, *J* = 13.0 Hz, 1H), 3.90 (d, *J* = 13.0 Hz, 1H), 2.94 (s, 1H), 1.55 – 1.51 (m, 1H), 1.28 -1.24 (m, 1H), 0.87 – 0.83 (m, 1H), 0.76 – 0.72 (m, 1H); ^{13}C NMR (126 MHz, $CDCl_3$): δ 172.0, 170.4, 137.7, 136.0, 135.8, 133.8, 133.3, 129.9, 129.6, 129.3, 129.3, 128.8, 128.6, 128.4, 127.4, 126.9, 126.8, 64.6, 49.3, 43.6, 41.3, 24.2, 14.0, 10.6; HRMS (ESI) m/z: $[M + H]^+$ Calcd for $C_{27}H_{27}Cl_2N_3O_2H$ 496.1558; Found 496.1545.

N-(2-chlorobenzyl)-1-(1-((2-chlorobenzyl)amino)-2-((2-methoxy-6-methylbenzyl) amino)-2-oxoethyl)cyclopropane-1-carboxamide (16f)

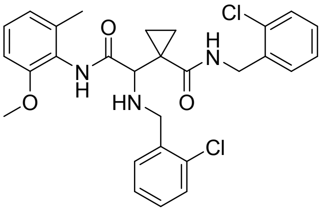

140 mg, 60% yield, amorphous white solid. ^{1}H NMR (500 MHz, $CDCl_3$): δ 9.33 – 9.31 (m, 1H), 8.02 (d, *J* = 6.0 Hz, 1H), 7.35 – 7.31 (m, 2H), 7.27 – 7.26 (m, 2H), 7.21 – 7.18 (m, 2H), 7.14 – 7.07 (m, 3H), 6.81 (d, *J* = 7.5 Hz, 1H), 6.71 (d, *J* = 8.0 Hz, 1H), 4.51 (dd, J_1 = 6 Hz, J_2 = 3.5 Hz, 2H), 4.03 – 3.96 (m, 2H), 3.73 (s, 3H), 3.37 (br, 1H), 2.88 (s, 1H), 2.16 (s, 3H), 1.40 – 1.39 (m, 2H), 1.02 – 1.00 (m, 2H); ^{13}C NMR (125 MHz, $CDCl_3$): δ 172.3, 169.1, 153.7, 136.4, 136.1, 135.8, 133.7, 133.1, 129.9, 129.5, 129.2, 129.1, 128.6, 128.2, 127.3, 126.8, 126.7, 123.5, 122.4, 108.1, 64.4, 55.2, 49.2, 41.2, 24.7, 18.4, 13.5, 10.8; HRMS (ESI) m/z: $[M + H]^+$ Calcd for $C_{28}H_{29}Cl_2N_3O_3H$ 526.1664; Found 526.1662.

N-(2-chlorobenzyl)-1-(1-((2-chlorobenzyl)amino)-2-oxo-2-((2,4,6-trimethylbenzyl) amino)ethyl)cyclopropane-1-carboxamide (16g)

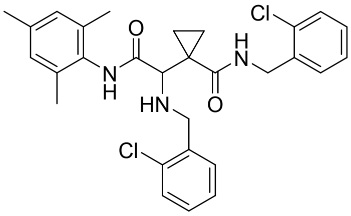

79 mg, 43% yield, amorphous white solid. ^{1}H NMR (500 MHz, $CDCl_3$): δ 9.39 – 9.36 (m,1H), 7.73 (s, 1H), 7.36 – 7.28 (m, 4H), 7.23 – 7.14 (m, 4H), 6.86 (s, 2H), 4.62 - 4.58 (m, 1H), 4.45 – 4.41 (m, 1H), 4.11 – 4.09 (m, 1H), 4.01 - 3.98 (m, 1H), 3.18 (br, 1H), 3.07 (s, 1H), 2.26 (s, 3H), 2.07 (s, 6H), 1.72 – 1.68 (m, 1H), 1.34 – 1.30 (m, 1H), 1.02 – 0.98 (m, 1H), 0.88 – 0.34 (m, 1H); ^{13}C NMR (125 MHz, $CDCl_3$): δ 171.8, 169.1, 137.1, 136.1, 135.8, 134.8, 133.8, 133.6, 130.5, 129.9, 129.8, 129.7, 129.4, 128.9, 128.8, 128.6, 127.0, 126.9, 65.6, 49.5, 41.5, 24.1, 20.9, 18.3, 15.3, 10.7; HRMS (ESI) m/z: $[M + H]^+$ Calcd for $C_{29}H_{31}Cl_2N_3O_3H$ 524.1872; Found 524.1872.

1-(2-(tert-butylamino)-1-((4-isopropylphenyl)amino)-2-oxoethyl)-N-(4-isopropylphenyl)cyclopropane-1-carboxamide (16h)

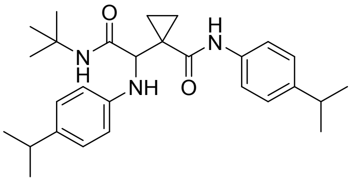

47 mg, 44% yield, amorphous white solid. ^{1}H NMR (500 MHz, $CDCl_3$): δ 8.39 (br, 1H), 7.38 (d, *J* = 8.5 Hz, 2H), 7.16 (d, *J* = 8.5 Hz, 2H), 7.06 (d, *J* = 8.5 Hz, 2H), 6.89 (br, 1H), 6.62 (d, *J* = 8.5 Hz, 2H), 4.83 (d, *J* = 5.5 Hz, 1H), 3.68 – 3.67 (m, 1H), 2.84 (pd, J_1 = 24.0 Hz, J_2 = 7.0 Hz, 2H), 1.29 (s, 9H), 1.22 (dd, J_1 = 7.0 Hz, J_2 = 5.0 Hz, 14H), 1.19 – 1.16 (m, 1H), 0.91 – 0.84 (m, 2H); ^{13}C NMR (125 MHz, $CDCl_3$): δ 171.0, 170.4, 145.1, 144.8, 140.0, 135.3, 127.2, 126.8, 120.2, 114.6, 63.1, 51.3, 33.6, 33.2, 29.3,

28.5, 24.1, 24.0, 12.7, 11.5; HRMS (ESI) m/z: $[M + H]^+$ Calcd for $C_{28}H_{39}N_3O_2H$ 450.3120; Found 450.3110.

N-(2-chlorophenyl)-1-(1-((2-chlorophenyl)amino)-2-(cyclooctylamino)-2-oxoethyl)cyclopropane-1-carboxamide (16i)

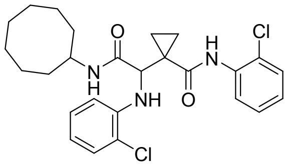

38 mg, 39% yield, amorphous white solid. ^1H NMR (500 MHz, $CDCl_3$): δ 8.31 (dd, J_1 = 8.5 Hz, J_2 = 1.5 Hz, 1H), 8.04 – 8.03 (m, 1H), 7.36 (dd, J_1 = 8.0 Hz, J_2 = 1.5 Hz, 1H), 7.31 – 7.28 (m, 2H), 7.12 (td, J_1 = 8.0 Hz, J_2 = 1.5 Hz, 1H), 7.07 (td, J_1 = 7.5 Hz, J_2 = 1.5 Hz, 1H), 6.89 (d, J = 8.0 Hz, 1H), 6.71 (td, J_1 = 7.5 Hz, J_2 = 1.5 Hz, 1H), 6.57 (dd, J_1 = 8.0 Hz, J_2 = 1.5 Hz, 1H), 5.67 (d, J = 5.0 Hz, 1H), 4.25 (d, J = 5.5 Hz, 1H), 4.01 - 3.96 (m, 1H), 1.77 – 1.69 (m, 2H), 1.59 – 1.45 (m, 12H), 1.31 – 1.25 (m, 3H), 1.11 – 1.07 (m, 1H); ^{13}C NMR (126 MHz, $CDCl_3$): δ 171.7, 169.9, 66.0, 48.2, 44.3, 44.0, 43.2, 36.0, 32.74, 32.67, 29.8, 29.6, 29.3, 25.4, 24.5, 24.4, 23.5, 14.2, 10.1; HRMS (ESI) m/z: $[M + H]^+$ Calcd for $C_{26}H_{31}Cl_2N_3O_2H$ 488.1871; Found 488.1856.

1-(2-(benzylamino)-2-oxo-1-(propylamino)ethyl)-N-propylcyclopropane-1-carboxamide (16j)

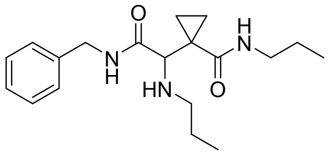

52 mg, 52% yield, amorphous white solid. ^1H NMR (500 MHz, $CDCl_3$): δ 9.05 (t, J = 5.5 Hz, 1H), 7.32 – 7.29 (m, 2H), 7.27 – 7.21 (m, 3H), 6.80 (br, 1H), 4.44 (d, J = 6.0 Hz, 2H), 3.15 – 3.10 (m, 2H), 2.78 – 2.75 (m, 2H), 2.54 – 2.49 (m, 1H), 1.57 – 1.51 (m, 3H), 1.46 – 1.39 (m, 2H), 1.19 – 1.14 (m, 1H), 0.96 (t, J = 7.5 Hz, 3H), 0.86 (t, J = 7.5 Hz, 3H), 0.82 – 0.78 (m, 1H), 0.65 – 0.61 (m, 1H); ^{13}C NMR (125 MHz, $CDCl_3$): δ 171.7, 171.0, 137.8, 128.7, 127.5, 127.4, 66.2, 66.2, 49.9, 43.6, 41.1, 23.2, 23.2, 22.6, 14.6, 14.6, 11.8, 11.4, 10.1; HRMS (ESI) m/z: $[M + H]^+$ Calcd for $C_{19}H_{29}N_3O_2H$ 332.2338; Found 332.2335.

1-(2-(cyclohexylamino)-1-(neopentylamino)-2-oxoethyl)-N-neopentyl cyclopropane-1-carboxamide (16k)

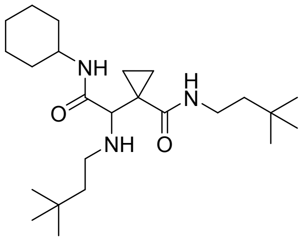

40.4 mg, 50% yield, amorphous white solid, mp 110-112°. ^1H NMR (500 MHz, $CDCl_3$): δ 8.87 (t, J = 5.0 Hz, 1H), 6.29 (d, J = 8.5 Hz, 1H), 3.79 – 3.72 (m, 1H), 3.20 – 3.15 (m, 2H), 2.72 (s, 1H), 2.51 – 2.46 (m, 1H), 1.84 – 1.80 (m, 2H), 1.67 – 1.63 (m, 2H), 1.56 (td, J_1 = 12.5 Hz, J_2 = 4.0 Hz, 1H), 1.50 – 1.47 (m, 1H), 1.43 – 1.40 (m, 2H), 1.37 – 1.33 (m, 3H), 1.20 – 1.14 (m, 3H), 0.89 (d, J = 4.0 Hz, 18H), 0.79 – 0.75 (m, 1H),

0.62 (ddd, J_1 = 10.0 Hz, J_2 = 6.5 Hz, J_3 = 3.5 Hz, 1H); ^{13}C NMR (126 MHz, $CDCl_3$): δ 171.7, 169.9, 66.0, 48.2, 44.3, 44.0, 43.2, 36.0, 32.74, 32.67, 29.8, 29.6, 29.3, 25.4, 24.5, 24.4, 23.5, 14.2, 10.1; HRMS (ESI) m/z: $[M + H]^+$ Calcd for $C_{24}H_{45}N_3O_2H$ 408.3590; Found 408.3580.

_N_-(4-bromobenzyl)-1-(1-((4-bromobenzyl)amino)-2-(tert-butylamino)-2-oxoethyl) cyclopropane-1-carboxamide (16l)

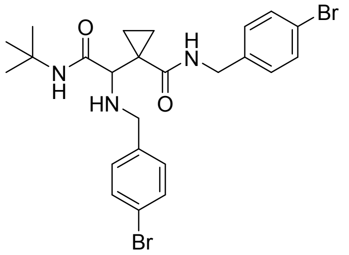

60 mg, 43% yield, amorphous white solid. ^{1}H NMR (500 MHz, $CDCl_3$): δ 9.24 (br, 1H), 7.43 (d, J = 8.5 Hz, 2H), 7.38 (d, J = 8.0 Hz, 2H), 7.12 (d, J = 8.5 Hz, 2H), 7.00 (d, J = 8.5 Hz, 2H), 6.10 (br, 1H), 4.34 (dd, J_1 = 15 Hz, J_2 = 5.5 Hz, 2H), 3.83 (d, J = 13.0 Hz, 1H), 3.66 (d, J = 13.5 Hz, 1H), 2.78 (s, 1H), 1.50 – 1.46 (m, 1H), 1.30 (s, 9H), 1.27 – 1.25 (m, 1H), 0.79 (ddd, J_1 = 10.0 Hz, J_2 = 6.5 Hz, J_3 = 4.0 Hz, 1H), 0.69 (ddd, J_1 = 10.0 Hz, J_2 = 6.5 Hz, J_3 = 3.7 Hz, 1H); ^{13}C NMR (125 MHz, $CDCl_3$): δ 171.9, 169.4, 137.7, 137.6, 131.74, 131.69, 129.7, 129.4, 121.3, 121.1, 64.4, 51.7, 50.9, 42.9, 28.6, 23.9, 14.3, 10.4; HRMS (ESI) m/z: $[M + H]^+$ Calcd for $C_{24}H_{29}Br_2N_3O_2H$ 550.0704; Found 550.0697.

1-(2-((2-chloro-6-methylphenyl)amino)-1-((4-chlorobenzyl)amino)-2-oxoethyl)-_N_-(4-chlorobenzyl)cyclopropane-1-carboxamide (16m)

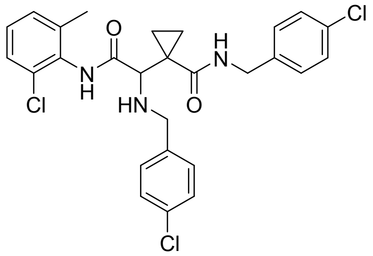

151 mg, 72% yield, amorphous yellow solid. ^{1}H NMR (500 MHz, $CDCl_3$): δ 9.17 (t, J = 5.6 Hz, 1H), 8.28 – 8.19 (m, 1H), 7.25 – 7.19 (m, 5H), 7.14 (d, J = 8.5 Hz, 2H), 7.11 (d, J = 5.5 Hz, 2H), 7.06 (d, J = 8.4 Hz, 2H), 4.44 (dd, J_1 = 15.0 Hz, J_2 = 6.0 Hz, 1H), 4.21 (dd, J_1 = 15.0 Hz, J_2 = 5.0 Hz, 1H), 3.88 (d, J = 13.1 Hz, 1H), 3.76 (d, J = 13.1 Hz, 1H), 3.15 (br, 1H), 2.91 (s, 1H), 2.14 (s, 3H), 1.54 – 1.48 (m, 1H), 1.33 – 1.28 (m, 1H), 0.92 (d, J = 3.0 Hz, 2H); ^{13}C NMR (125 MHz, $CDCl_3$): δ 171.8, 169.4, 137.7, 136.9, 136.8, 133.1, 132.9, 131.9, 131.1, 129.2, 129.1, 128.9, 128.6, 128.1, 127.0, 64.5, 50.8, 42.8, 24.1, 24.1, 18.7, 14.7, 10.9; HRMS (ESI) m/z: $[M + H]^+$ Calcd for $C_{27}H_{26}Cl_3N_3O_2H$ 530.1168; Found 530.1165.

_N_-benzyl-1-(1-(benzylamino)-2-(tert-butylamino)-2-oxoethyl)cyclopropane-1-carboxamide (16n)

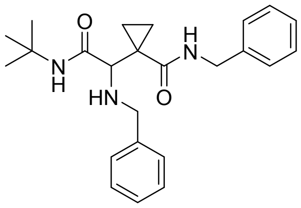

33 mg, 41% yield, white crystalline needles, mp 113-115°. ^{1}H NMR (500 MHz, $CDCl_3$): δ 9.29 (t, J = 5.6 Hz, 1H), 7.33 – 7.28 (m, 2H), 7.28 – 7.21 (m, 6H), 7.12 (dd, J_1 = 7.5 Hz, J_2 = 2.0 Hz, 2H), 6.21 (s, 1H), 4.39 (d, J = 5.5 Hz, 2H), 3.86 (d, J = 13.0 Hz, 1H), 3.70 (d, J = 13.0 Hz, 1H), 2.84 (s, 1H), 1.46 – 1.41 (m, 1H), 1.30 (s, 9H), 0.89 – 0.83 (m, 1H), 0.81 - 0.77 (m, 1H), 0.75 – 0.71 (m, 1H); ^{13}C NMR (125 MHz, $CDCl_3$): δ 172.0, 169.6, 138.7, 138.6, 128.5, 128.1, 127.6, 127.3, 127.1, 64.4, 51.6, 51.5, 43.5, 28.5, 23.9, 14.0, 10.3; HRMS (ESI) m/z: $[M + H]^+$ Calcd for $C_{24}H_{31}N_3O_2H$ 394.2494; Found 394.2489.

2,2,2-trifluoroethyl 1-(2-(benzylamino)-1-hydroxy-2-oxoethyl)cyclopropane-1-carboxylate (17a)

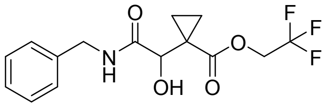

To a stirred solution of 1-formylcyclopropane-1-carboxylic acid (0.114 g, 1 mmol, 1 equiv.) in TFE (1 mL, 1 M), benzyl isocyanide (0.117 g, 1 mmol, 1 equiv.) was added. The reaction mixture was stirred at rt overnight. The solvent was removed under reduced pressure and the reaction mixture was purified with column chromatography (PE - EtOAc 7:1-3:1) to yield compounds **17a** (278 mg, 84% yield) as amorphous white solid. ^{1}H NMR (500 MHz, $CDCl_3$): δ 8.26 (s, 1H), 7.35 - 7.33 (m, 2H), 7.32 – 7.28 (m, 3H), 4.50 – 4.47 (m, 2H), 4.46 – 4.33 (m, 3H), 1.44 (ddd, J_1 = 11.0 Hz, J_2 = 7.5 Hz, J_3 = 3.0 Hz, 2H), 1.36 – 1.32 (m, 1H), 1.12 (td, J_1 = 8.5 Hz, J_2 = 4.5 Hz Hz, 1H); ^{13}C NMR (126 MHz, $CDCl_3$) δ 173.7, 171.7, 137.7, 128.8, 128.7, 127.6, 127.6, 121.5, 70.3, 60.6 (d, J_{C-F} = 37.5 Hz), 43.6, 26.9, 14.7, 12.9; HRMS (ESI) m/z: $[M + H]^+$ Calcd for $C_{15}H_{16}F_3NO_4H$ 332.1110; Found 332.1220.

2,2,2-trifluoroethyl 1-(1-hydroxy-2-((2-nitrobenzyl)amino)-2-oxoethyl) cyclopropane-1-carboxylate (17b)

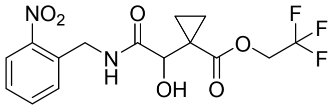

102 mg, 80% yield, amorphous white solid. ^{1}H NMR (500 MHz, $CDCl_3$): δ 8.05 (d, J = 8.0 Hz, 1H), 7.62 – 7.60 (m, 2H), 7.47 (dt, J_1 = 9.0 Hz, J_2 = 4.5 Hz, 1H), 4.73 (dd, J_1 = 6.0 Hz, J_2 = 3.5 Hz, 2H), 4.47 – 4.38 (m, 2H), 4.16 (s, 1H), 1.45 – 1.37 (m, 2H), 1.29 (ddd, J_1 = 10.0 Hz, J_2 = 7.2 Hz, J_3 = 4.5 Hz, 1H), 1.02 (ddd, J_1 = 9.0 Hz, J_2 = 7.5 Hz, J_3 = 4.5 Hz, 1H); ^{13}C NMR (125 MHz, $CDCl_3$): δ 173.2, 172.1, 134.0, 133.7, 133.1, 131.9, 128.8, 125.1, 70.9, 69.8, 60.4 (q, J_{C-F} = 36.0 Hz), 41.4, 26.9, 14.7, 12.9; HRMS (ESI) m/z: $[M + H]^+$ Calcd for $C_{15}H_{15}F_3N_2O_6H$ 377.0960; Found 377.0959.

2,2,2-trifluoroethyl 1-(2-((4-fluorophenyl)amino)-1-hydroxy-2-oxoethyl) cyclopropane-1-carboxylate (17c)

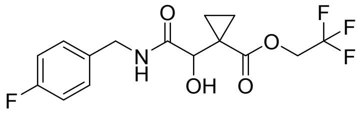

180 mg, 90% yield, amorphous white solid. ^{1}H NMR (500 MHz, $CDCl_3$): δ 7.23 (dd, J_1 = 8.5 Hz, J_2 = 5.5 Hz, 2H), 7.02 (t, J = 8.8 Hz, 2H), 4.54 – 4.34 (m, 4H), 4.21 (d, J = 6.5 Hz, 1H), 3.47 (d, J = 7.0 Hz, 1H), 1.49 – 1.43 (m, 2H), 1.33 (ddd, J_1 = 10.0 Hz, J_2 = 7.0 Hz, J_3 = 4.5 Hz, 1H), 1.12 (ddd, J_1 = 9.0 Hz, J_2 = 7.5 Hz, J_3 = 4.5 Hz, 1H); ^{13}C NMR (125 MHz, $CDCl_3$): δ 173.7, 171.9, 163.3, 161.4, 133.8, 133.7, 129.5, 129.4, 123.9, 121.7, 115.8, 115.6, 71.0, 70.9, 60.7 (q, $J_{C\text{-}F}$ = 36.6 Hz), 43.0, 27.1, 14.9, 13.2; HRMS (ESI) m/z: [M + H]$^+$ Calcd for $C_{15}H_{15}F_4NO_4H$ 350.1015; Found 350.1011.

2,2,2-trifluoroethyl 1-(1-hydroxy-2-oxo-2-(phenylamino)ethyl)cyclopropane-1-carboxylate (17d)

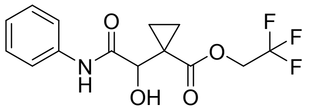

245 mg, 89% yield, amorphous white solid. ^{1}H NMR (500 MHz, $CDCl_3$): δ 8.70 (s, 1H), 7.55 – 7.53 (m, 1H), 7.36 – 7.32 (m, 3H), 7.15 – 7.12 (m, 1H), 4.54 – 4.40 (m, 2H), 4.31 (s, 1H), 1.53 – 1.48 (m, 2H), 1.37 (ddd, J_1 = 10.0 Hz, J_2 = 7.5 Hz, J_3 = 4.5 Hz, 1H), 1.19 – 1.13 (m, 1H); ^{13}C NMR (125 MHz, $CDCl_3$): δ 169.8, 137.2, 129.5, 129.1, 124.7, 123.6, 119.8, 118.9, 71.1, 60.7 (q, $J_{C\text{-}F}$ = 25.0 Hz), 26.9, 14.9, 13.1; HRMS (ESI) m/z: [M + H]$^+$ Calcd for $C_{14}H_{14}F_3NO_4H$ 318.0953; Found 318.0948.

2,2,2-trifluoroethyl 1-(1-hydroxy-2-(mesitylamino)-2-oxoethyl)cyclopropane-1-carboxylate (17e)

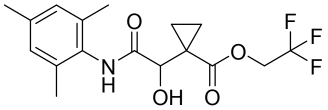

103 mg, 97% yield, amorphous white solid, mp 106-108°. ^{1}H NMR (500 MHz, $CDCl_3$): δ 8.03 (s, 1H), 6.90 (s, 2H), 4.61 (s, 1H), 4.59 – 4.53 (m, 1H), 4.47 – 4.40 (m, 1H), 2.27 (s, 3H), 2.15 (s, 6H), 1.61 – 1.57 (m, 1H), 1.49 – 1.45 (m, 1H), 1.40 – 1.36 (m, 1H), 1.18 – 1.13 (m, 1H); ^{13}C NMR (126 MHz, $CDCl_3$): δ 174.4, 170.6, 137.2, 134.8, 130.3, 129.0, 123.7, 121.5, 68.8, 60.6 (q, $J_{C\text{-}F}$ = 36.5 Hz), 26.9, 20.9, 18.2, 14.5, 12.5; HRMS (ESI) m/z: [M + H]$^+$ Calcd for $C_{17}H_{20}F_3NO_4H$ 360.1422; Found 360.1420.

isopropyl 1-(1-hydroxy-2-(mesitylamino)-2-oxoethyl)cyclopropane-1-carboxylate (17f)

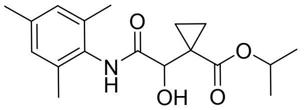

89 mg, 88% yield, white crystalline needles, mp 108-110°. ^{1}H NMR (500 MHz, $CDCl_3$): δ 8.33 (s, 1H), 6.89 (s, 2H), 5.08 – 5.03 (sept, J = 6.2 Hz, 1H), 4.65 (s, 1H), 3.90 (br, 1H), 2.26 (s, 3H), 2.16 (s, 6H), 1.48 (ddd, J_1 = 9.5 Hz, J_2 = 7.5 Hz, J_3 = 4.0 Hz, 1H), 1.32 (ddd, J_1 = 10.0 Hz, J_2 = 7.5 Hz, J_3 = 4.5 Hz, 1H), 1.22 (dd, J_1 = 11.5 Hz, J_2 = 5.8 Hz, 7H), 0.99 (ddd, J_1 = 9.5 Hz, J_2 = 7.5 Hz, J_3 = 4.0 Hz, 1H); ^{13}C NMR (125 MHz, $CDCl_3$): δ 175.8, 171.3, 137.0, 134.9, 130.6, 128.9, 68.8, 68.5, 27.0, 21.67, 21.65,

20.9, 18.2, 13.5, 11.6; HRMS (ESI) m/z: $[M + H]^+$ Calcd for $C_{18}H_{25}NO_4H$ 320.1861; Found 320.1856.

S-phenyl 1-(2-(tert-butylamino)-1-hydroxy-2-oxoethyl)cyclopropane-1-carbothioate (17g)

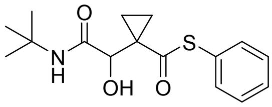

61 mg, 86% yield, amorphous white solid. ^1H NMR (500 MHz, $CDCl_3$): δ 7.43 – 7.42 (m, 3H), 7.37 – 7.35 (m, 2H), 6.55 (br, 1H), 4.30 (d, J = 6.5 Hz, 1H), 3.73 – 3.70 (m, 1H), 1.63 – 1.58 (m, 1H), 1.57 – 1.52 (m, 1H), 1.43 (ddd, J_1 = 9.7 Hz, J_2 = 7.2 Hz, J_3 = 5.2 Hz, 1H), 1.33 (s, 9H), 1.22 (ddd, J_1 = 9.5 Hz, J_2 = 7.5 Hz, J_3 = 5.0 Hz, 1H); ^{13}C NMR (125 MHz, $CDCl_3$): δ 202.5, 170.8, 135.1, 129.7, 129.3, 126.3, 69.7, 51.3, 37.0, 28.6, 15.2, 13.8; HRMS (ESI) m/z: $[M + H]^+$ Calcd for $C_{16}H_{21}NO_3SH$ 308.1320; Found 308.1317.

2,2,2-trifluoroethyl 1-(2-(hexylamino)-1-hydroxy-2-oxoethyl)cyclopropane-1-carboxylate (17h)

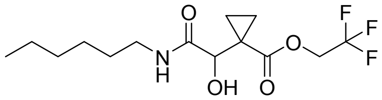

75 mg, 81% yield, amorphous yellow oil. ^1H NMR (500 MHz, $CDCl_3$): δ 6.73 (s, 1H), 4.52 (dq, J_1 = 13.0 Hz, J_2 = 8.5 Hz, 1H), 4.41 (dq, J_1 = 13.0 Hz, J_2 = 8.5 Hz, 1H), 4.25 (d, J = 6.5 Hz, 1H), 3.60 – 3.57 (m, 1H), 3.33 – 3.21 (m, 2H), 1.66 – 1.61 (m, 2H), 1.51 (dd, J_1 = 14.0 Hz, J_2 = 6.5 Hz, 2H), 1.47 – 1.44 (m, 1H), 1.41 (ddd, J_1 = 10.0 Hz, J_2 = 7.5 Hz, J_3 = 4.0 Hz, 1H), 1.32 – 1.28 (m, 7H), 1.09 (ddd, J_1 = 10.0 Hz, J_2 = 7.5 Hz, J_3 = 4.0 Hz, 1H), 0.89 – 0.87 (m, 3H); ^{13}C NMR (125 MHz, $CDCl_3$) δ 173.8, 171.6, 122.7 (d, J_{C-F} = 275.0 Hz) 69.6, 60.6 (q, J_{C-F} = 37.5 Hz), 39.6, 31.4, 29.3, 26.8, 26.4, 22.5, 14.6, 13.9, 12.7; HRMS (ESI) m/z: $[M + H]^+$ Calcd for $C_{14}H_{22}F_3NO_4H$ 326.15792; Found 326.1573.

2,2,2-trifluoroethyl 1-(2-((cyclooctylmethyl)amino)-1-hydroxy-2-oxoethyl) cyclopropane-1-carboxylate (17i)

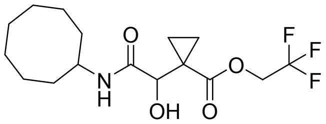

247 mg, 96% yield, amorphous white solid. ^1H NMR (500 MHz, $CDCl_3$): δ 6.71 (d, J = 8.0 Hz, 1H), 4.58 – 4.51 (m, 1H), 4.43 – 4.45 (m, 1H), 4.24 (s, 1H), 4.00 – 3.94 (m, 1H), 1.86 – 1.74 (m, 2H), 1.68 – 1.51 (m, 13H), 1.46 – 1.37 (m, 2H), 1.32 – 1.28 (m, 1H), 1.09 – 1.05 (m, 1H); ^{13}C NMR (125 MHz, $CDCl_3$): δ 173.7, 170.4, 123.8, 121.6, 69.7, 60.4 (q, J_{C-F} = 36.5 Hz), 49.6, 32.0, 31.8, 27.1, 27.1, 26.9, 25.3, 23.8, 23.5, 23.4, 20.7, 17.4, 17.2, 14.5, 12.6; HRMS (ESI) m/z: $[M + Na]^+$ Calcd for $C_{16}H_{25}F_3NO_4Na$ 374.1555; Found 374.1542.

2,2,2-trifluoroethyl 1-(2-(cyclohexylamino)-1-hydroxy-2-oxoethyl)cyclopropane-1-carboxylate (17j)

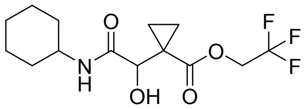

187 mg, 97% yield, yellow oil. ^{1}H NMR (500 MHz, $CDCl_3$): δ 6.63 (d, *J* = 8 Hz, 1H), 4.58-4.51 (m, 1H), 4.43-4.36 (m, 1H), 4.26 (dd, J_1 = 6.5 Hz, J_2 = 1.5 Hz, 1H), 3.79-3.72 (m, 1H), 3.63-3.60 (m, 1H), 1.92-1.88 (m, 1H), 1.85-1.82 (m, 1H), 1.72-1.65 (m, 3H), 1.62-1.57 (m, 1H), 1.45-1.28 (m, 6H), 1.23-1.13 (m, 4H), 1.10-1.05 (m, 1H); ^{13}C NMR (125 MHz, $CDCl_3$): δ 173.8, 170.7, 123.8, 121.6, 69.5 (d, $J_{C\text{-}F}$ = 9.5 Hz), 60.4 (q, $J_{C\text{-}F}$ = 36.5 Hz), 48.3, 32.8, 32.7, 26.9, 25.4, 24.6, 14.5, 12.6; HRMS (ESI) m/z: $[M + H]^+$ Calcd for $C_{14}H_{20}F_3NO_4H$ 324.1473; Found 324.1468.

methyl 1-(2-(tert-butylamino)-1-hydroxy-2-oxoethyl)cyclopropane-1-carboxylate (17k)

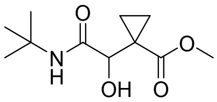

traces, amorphous white solid. ^{1}H NMR (500 MHz, $CDCl_3$): δ 4.13 (d, *J* = 6.5 Hz, 1H), 3.68 (s, 3H), 1.35 (s, 9H), 1.34 – 1.29 (m, 2H), 1.18 (ddd, J_1 = 10.0 Hz, J_2 = 7.5 Hz, J_3 = 4.3 Hz, 1H), 0.95 (ddd, J_1 = 9.6 Hz, J_2 = 7.5 Hz, J_3 = 4.3 Hz, 1H); ^{13}C NMR (125 MHz, $CDCl_3$): δ 175.9, 171.3, 69.9, 52.1, 51.2, 28.7, 26.8, 13.8, 11.9; HRMS (ESI) m/z: $[M + Na]^+$ Calcd for $C_{11}H_{19}NO_4Na$ 252.1211; Found 252.1202.

***N*-(tert-butyl)-9-oxo-5,8-diazaspiro[2.6]nonane-4-carboxamide (18)**

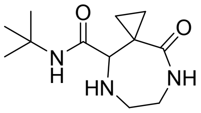

80 mg, 65% yield, yellow oil. ^{1}H NMR (500 MHz, $CDCl_3$): δ 6.37 (br s, 1H), 5.98 (br s, 1H), 3.47-3.42 (m, 1H), 3.37 (br s, 1H), 3.26-3.20 (m, 1H), 2.98-2.96 (m, 2H), 2.81 (s, 1H), 2.02 (br s, 6H), 1.55-1.51 (m, 1H), 1.35 (s, 9H), 1.08-1.04 (m, 1H), 1.03-0.98 (m, 1H); ^{13}C NMR (126 MHz, $CDCl_3$, DMSO): δ 175.5, 168.9, 62.8, 50.4, 45.6, 43.7, 28.0, 27.0, 11.4, 11.0; HRMS (ESI) m/z: $[M + H]^+$ Calcd for $C_{12}H_{21}N_3O_2H$ 240.1712; Found 240.1706.

1-(2-(tert-butylamino)-1-((3-hydroxypropyl)amino)-2-oxoethyl)-*N*-(3-hydroxypropyl)cyclopropane-1-carboxamide (16o)

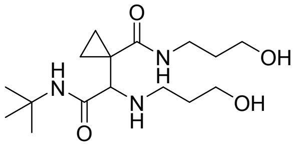

123 mg, 73% yield, yellow oil. ^{1}H NMR (500 MHz, $CDCl_3$): δ 8.87 (s, 1H), 6.20 (s, 1H), 3.78 (td, J_1 = 6.0 Hz, J_2 = 2.0 Hz, 2H), 3.59 (td, J_1 = 6.0 Hz, J_2 = 2.0 Hz, 2H), 3.41 (d, J_1 = 5.0 Hz, 1H), 3.40 – 3.33 (m, 2H), 2.92 – 2.87 (m, 1H), 2.77 (s, 1H), 2.70 – 2.64 (m, 1H), 1.78 - 1.75 (m, 2H), 1.66 – 1.64 (m, 2H), 1.33 (s, 9H), 1.24 – 1.19 (m, 1H), 0.85 – 0.80 (m, 1H), 0.75 – 0.71 (m, 1H); ^{13}C NMR (125 MHz, $CDCl_3$) δ 173.4, 170.1, 65.4, 60.8, 59.3, 55.1, 51.6, 45.2, 36.1, 32.1, 28.5, 24.8, 13.7, 10.7; HRMS (ESI) m/z: $[M + H]^+$ Calcd for $C_{16}H_{31}N_3O_4H$ 330.2392; Found 330.2387.

2,2,2-trifluoroethyl 1-(2-oxo-2-((2,4,6-trimethylbenzyl)amino)acetyl) cyclopropane-1-carboxylate (19a)

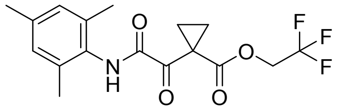

12 mg, 85% yield, amorphous white solid. ^{1}H NMR (500 MHz, $CDCl_3$): δ 8.02 (s, 1H), 6.92 (s, 2H), 4.48 (q, *J* = 8.5 Hz, 2H), 2.28 (s, 3H), 2.18 (s, 6H), 1.72 – 1.70 (m, 2H), 1.65 – 1.62 (m, 2H); ^{13}C NMR (125 MHz, $CDCl_3$): δ 192.7, 168.4, 157.9, 137.7, 134.8, 129.2, 129.1, 61.2 (q, J_{C-F} = 36.5 Hz), 31.8, 30.3, 29.7, 20.9, 18.3, 18.1; HRMS (ESI) m/z: $[M + H]^+$ Calcd for $C_{17}H_{18}F_3NO_4H$ 358.1266; Found 358.1261.

2,2,2-trifluoroethyl 1-(2-((2-chloro-6-methylphenyl)amino)-2-oxoacetyl) cyclopropane-1-carboxylate (19b)

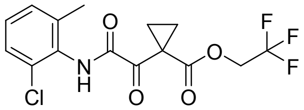

10 mg, 90% yield, amorphous white solid, mp 135-138 °C. ^{1}H NMR (500 MHz, $CDCl_3$): δ 8.31 (br s, 1H), 7.30 (t, *J* = 5 Hz, 1H), 7.19 (d, *J* = 5 Hz, 2H), 4.50 (q, *J* = 8.5 Hz, 2H), 2.28 (s, 3H); ^{13}C NMR (125 MHz, $CDCl_3$): δ 191.9, 168.3, 157.6, 137.7, 131.1, 130.7, 129.4, 128.6, 127.3, 123.8, 121.6, 61.2 (q, *J* = 36.5 Hz), 31.8, 18.8, 18.3; HRMS (ESI) m/z: $[M + H]^+$ Calcd for $C_{15}H_{13}ClF_3NO_4H$ 364.0563; Found 364.0555.

2,2,2-trifluoroethyl 1-(2-(hexylamino)-2-oxoacetyl)cyclopropane-1-carboxylate (19c)

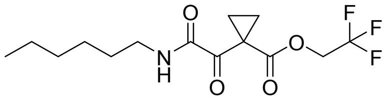

7 mg, 90% yield, amorphous white solid. ^{1}H NMR (500 MHz, $CDCl_3$): δ 6.75 (br s, 1H), 4.48 (q, *J* = 8.5 Hz, 2H), 3.31 (q, *J* = 6.5 Hz, 2H), 1.65-1.63 (m, 2H), 1.57-1.53 (m, 2H), 1.34-1.25 (m, 8H), 0.88 (t, *J* = 6.5 Hz, 3H); ^{13}C NMR (125 MHz, $CDCl_3$): δ 192.7, 168.5, 159.8, 123.9, 121.7, 61.3 (q, *J* = 36.5 Hz), 39.5, 31.7, 31.3, 29.7, 29.1, 26.4, 22.5, 17.9, 13.9; HRMS (ESI) m/z: $[M + H]^+$ Calcd for $C_{14}H_{20}F_3NO_4H$ 326.1579; Found 326.1573.

2,2,2-trifluoroethyl 1-(2-(cyclohexylamino)-2-oxoacetyl)cyclopropane-1-carboxylate (19d)

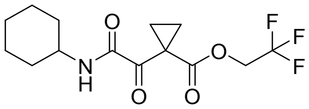

8 mg, 92% yield, amorphous white solid. ^{1}H NMR (500 MHz, $CDCl_3$): δ 6.63 (br s, 1H), 4.47 (q, *J* = 8.5 Hz, 2H), 3.77-3.71 (m, 1H), 1.94-1.90 (m, 2H), 1.76-1.72 (m, 2H), 1.54-1.52 (m, 2H), 1.42-1.33 (m, 2H), 1.27-1.18 (m, 4H); ^{13}C NMR (125 MHz, $CDCl_3$): δ 192.8, 168.5, 158.9, 123.9, 121.7, 61.3 (q, *J* = 36.5 Hz), 48.7, 32.5, 31.7, 29.7, 25.3, 24.6, 17.8; HRMS (ESI) m/z: $[M + H]^+$ Calcd for $C_{14}H_{18}F_3NO_4H$ 306.1317; Found 306.1314.

5-(4-fluorobenzyl)-5-azaspiro[2.4]heptane-4,6,7-trione (21a)

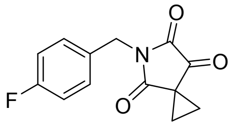

7 mg, 30% yield, amorphous white solid. ^{1}H NMR (500 MHz, $CDCl_3$): δ 7.51 – 7.42 (m, 2H), 7.05 – 6.99 (m, 2H), 4.87 (s, 2H), 2.00 – 1.95 (m, 2H), 1.90 – 1.85 (m, 2H); ^{13}C NMR (125 MHz, $CDCl_3$): δ 192.2, 172.8, 163.8, 161.9, 160.9, 131.4, 131.3, 130.8, 130.8, 116.0, 115.9, 42.4, 31.0, 22.4; HRMS (ESI) m/z: $[M + H]^+$ Calcd for $C_{13}H_{10}FNO_3H$ 248.0723; Found 248.0714.

5-phenyl-5-azaspiro[2.4]heptane-4,6,7-trione (21b)

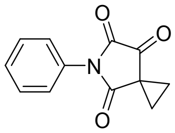

8 mg, 10% yield, orange oil. ^{1}H NMR (500 MHz, $CDCl_3$): δ 7.53 (t, J = 7.7 Hz, 2H), 7.46 (t, J = 7.5 Hz, 1H), 7.40 (d, J = 7.1 Hz, 2H), 2.14 – 2.09 (m, 2H), 2.05 – 2.01 (m, 2H); ^{13}C NMR (126 MHz, $CDCl_3$): δ 206.7, 192.5, 172.1, 130.8, 129.3, 129.2, 125.9, 30.9, 23.0; HRMS (ESI) m/z: $[M + H]^+$ Calcd for $C_{12}H_9NO_3H$ 216.0660; Found 216.0652.

5-(2-nitrobenzyl)-5-azaspiro[2.4]heptane-4,6,7-trione (21c)

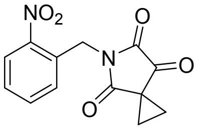

6 mg, 28% yield, white crystalline needles, mp 139-141°. ^{1}H NMR (500 MHz, $CDCl_3$): δ 8.10 (dd, J_1 = 8.5 Hz, J_2 = 1.5 Hz, 1H), 7.62 (td, J_1 = 7.5 Hz, J_2 = 1.5 Hz, 1H), 7.50 (td, J_1 = 7.5 Hz, J_2 = 1.3 Hz, 1H), 7.29 (dd, J_1 = 7.8 Hz, J_3 = 1.3 Hz, 1H), 5.37 (s, 2H), 2.06 – 2.04 (m, 2H), 1.98 – 1.95 (m, 2H); ^{13}C NMR (125 MHz, $CDCl_3$): δ 191.5, 172.5, 160.7, 148.5, 133.8, 129.6, 129.1, 128.8, 125.5, 39.8, 31.1, 29.7, 22.6; HRMS (ESI) m/z: $[M + H]^+$ Calcd for $C_{13}H_{10}N_2O_5H$ 275.0667; Found 275.0661.

4. Plausible mechanisms

- Mechanism for Ugi three center-four component reaction (U3C-4CR)

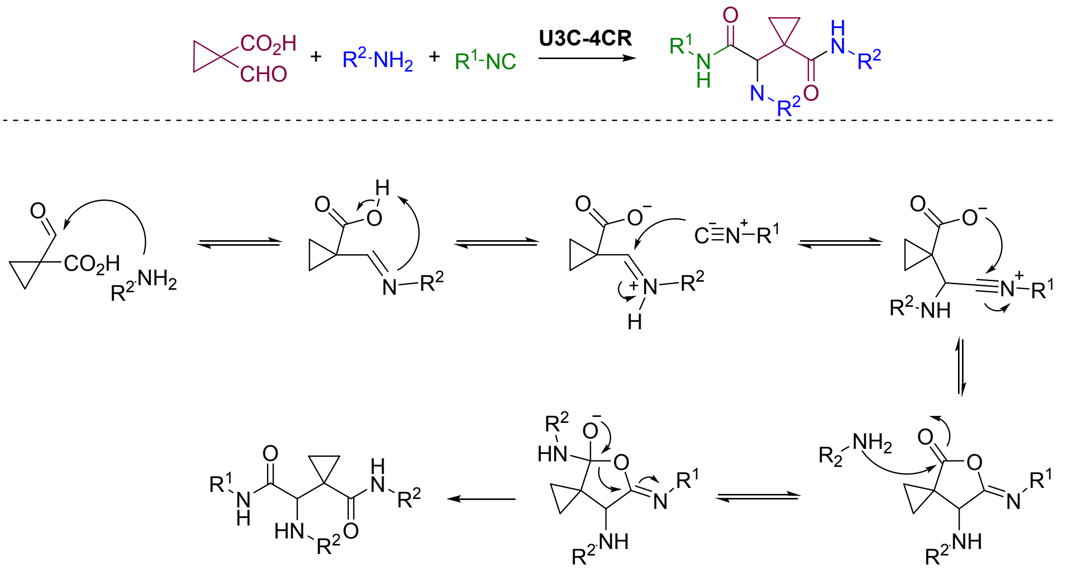

- Mechanism for Passerini two center-three-component reaction (P2C-3CR)

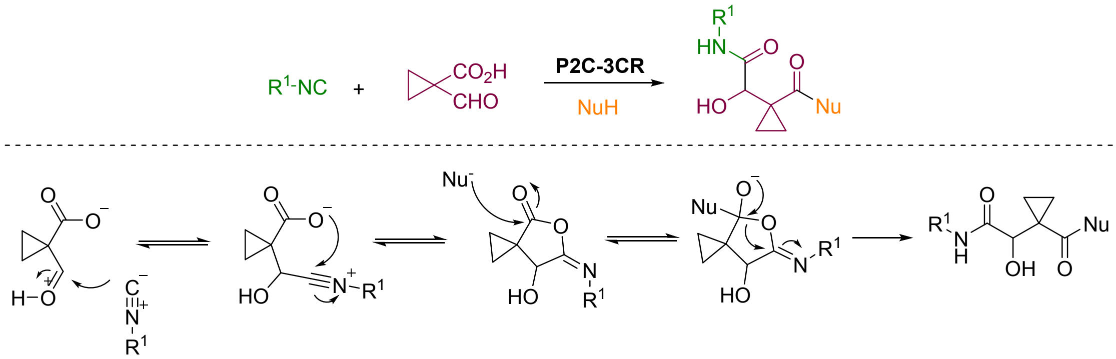

- Mechanism for PCC cyclization reaction

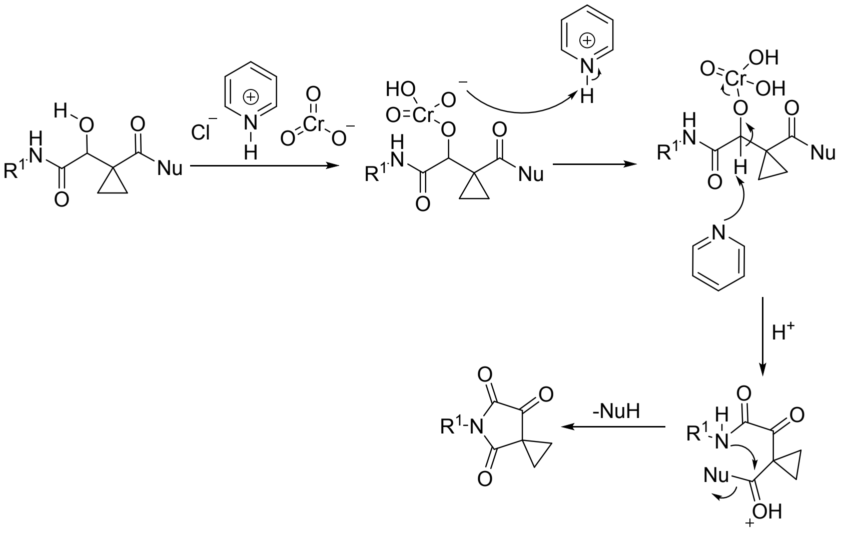

5. Exemplary copies of NMR spectra of novel compounds

^{1}H NMR spectrum (500 MHz, $CDCl_3$) of compound 20

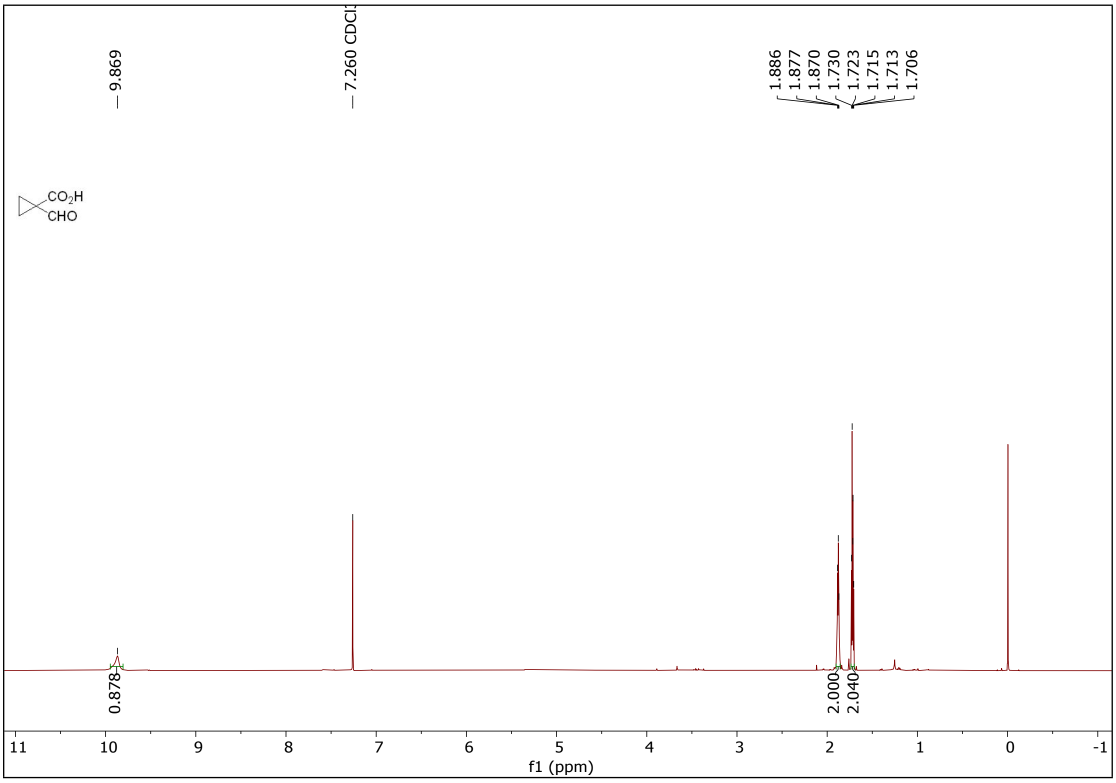

^{1}H NMR spectrum (500 MHz, $CDCl_3$) of compound 16a

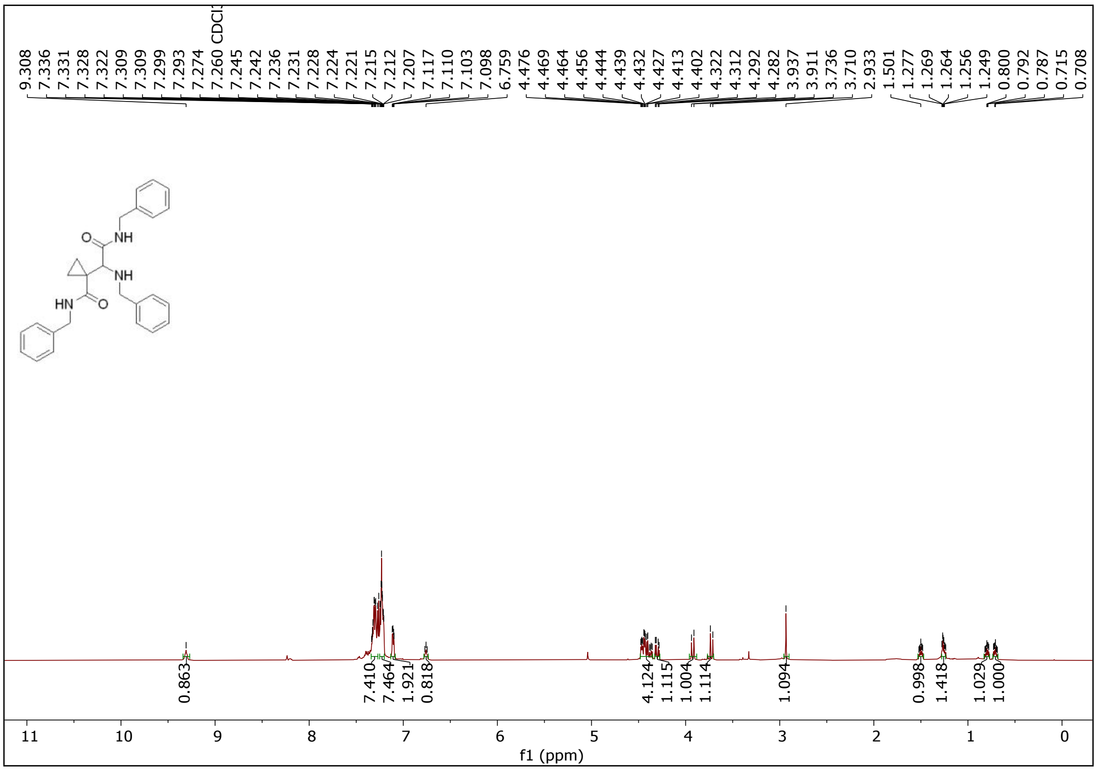

^{13}C NMR spectrum (125 MHz, $CDCl_3$) of compound 16a

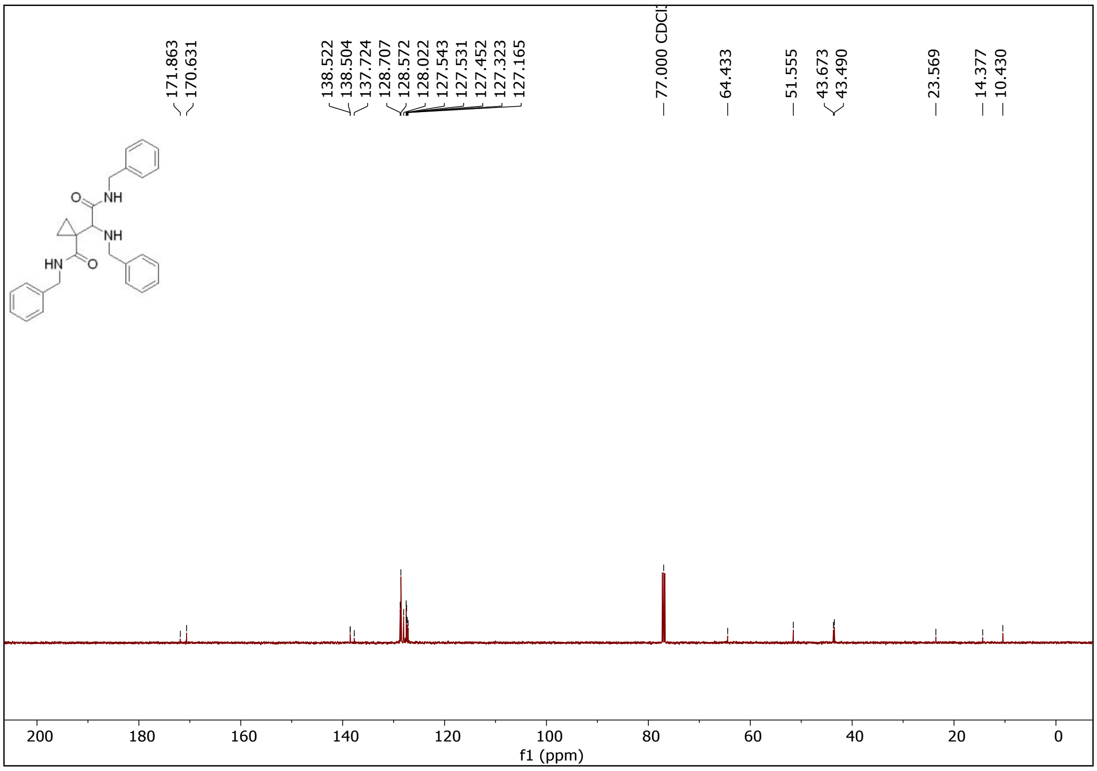

^{1}H NMR spectrum (500 MHz, $CDCl_3$) of compound 16b

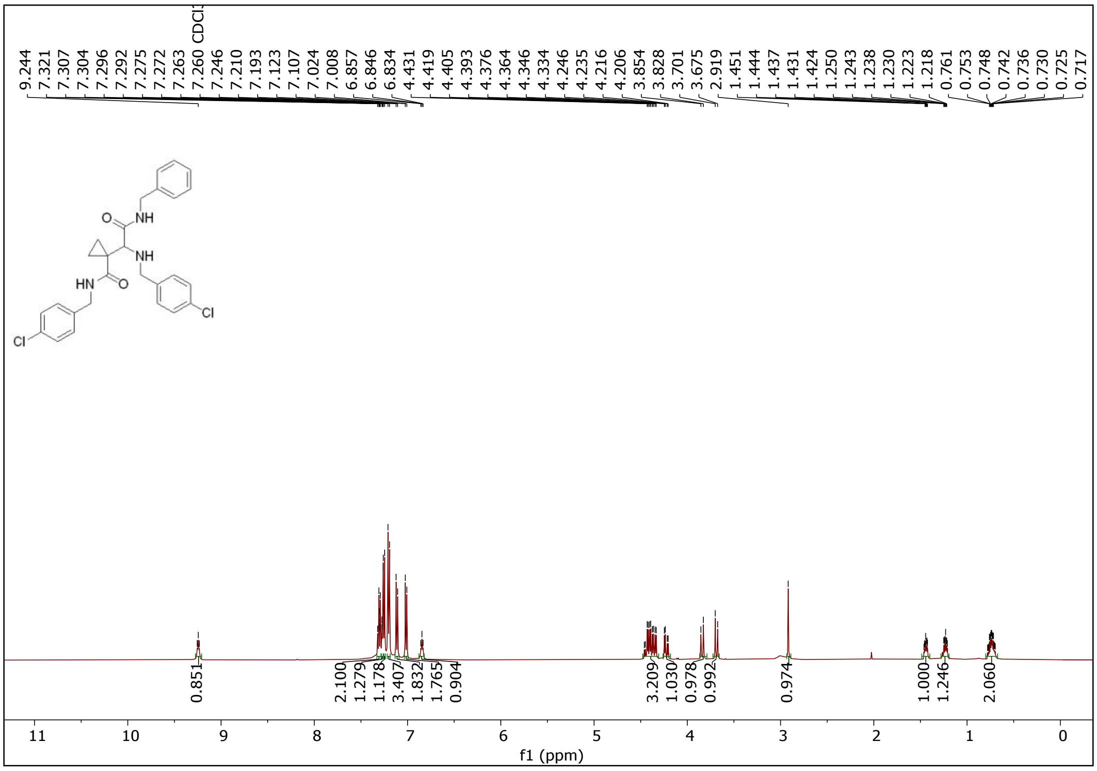

^{13}C NMR spectrum (125 MHz, $CDCl_3$) of compound 16b

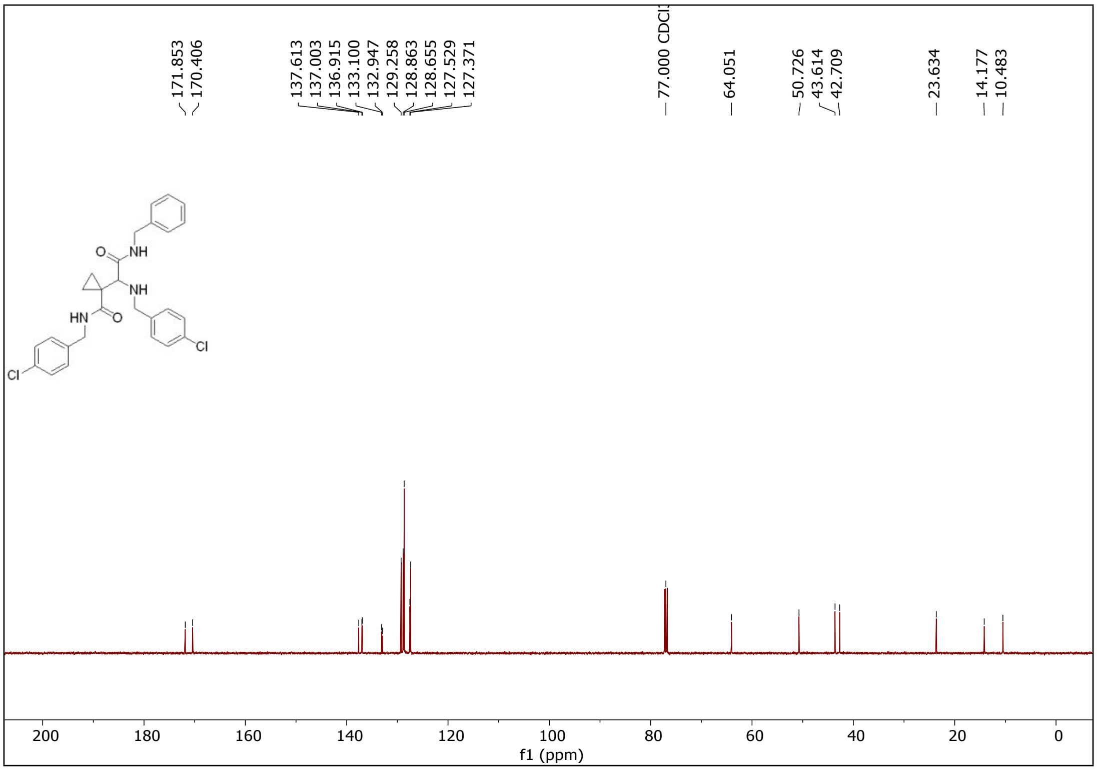

^{1}H NMR spectrum (500 MHz, $CDCl_3$) of compound 16c

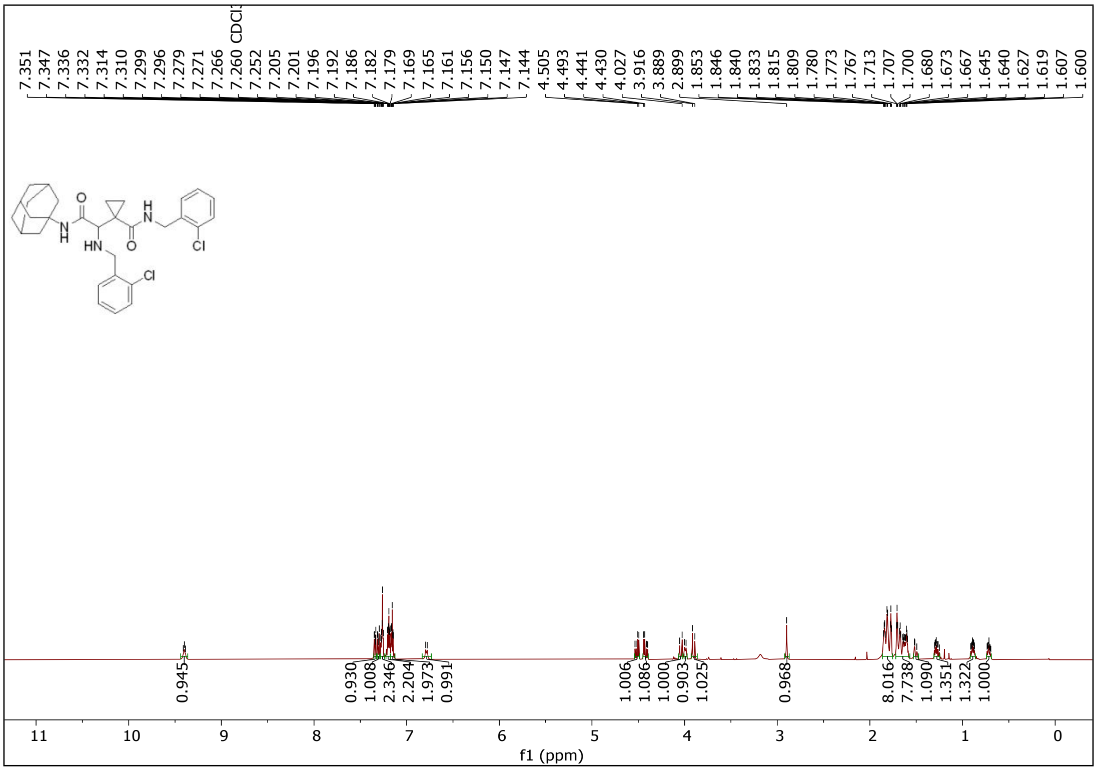

^{13}C NMR spectrum (125 MHz, $CDCl_3$) of compound 16c

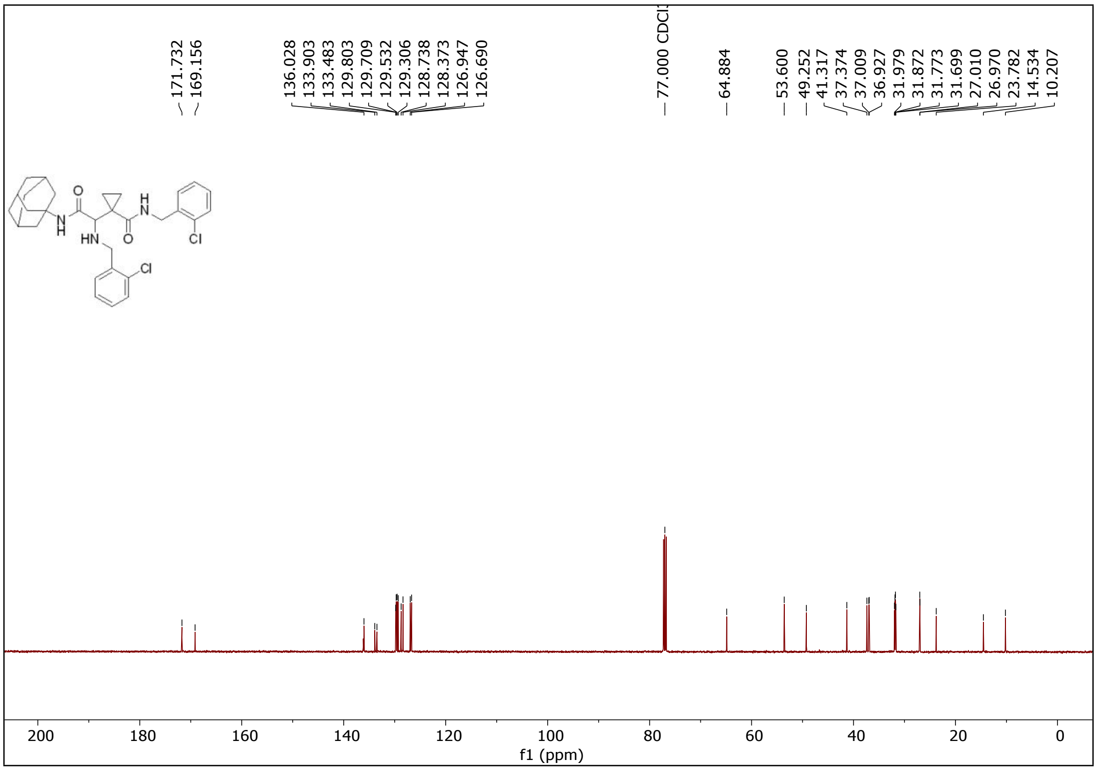

^{1}H NMR spectrum (500 MHz, $CDCl_3$) of compound 16d

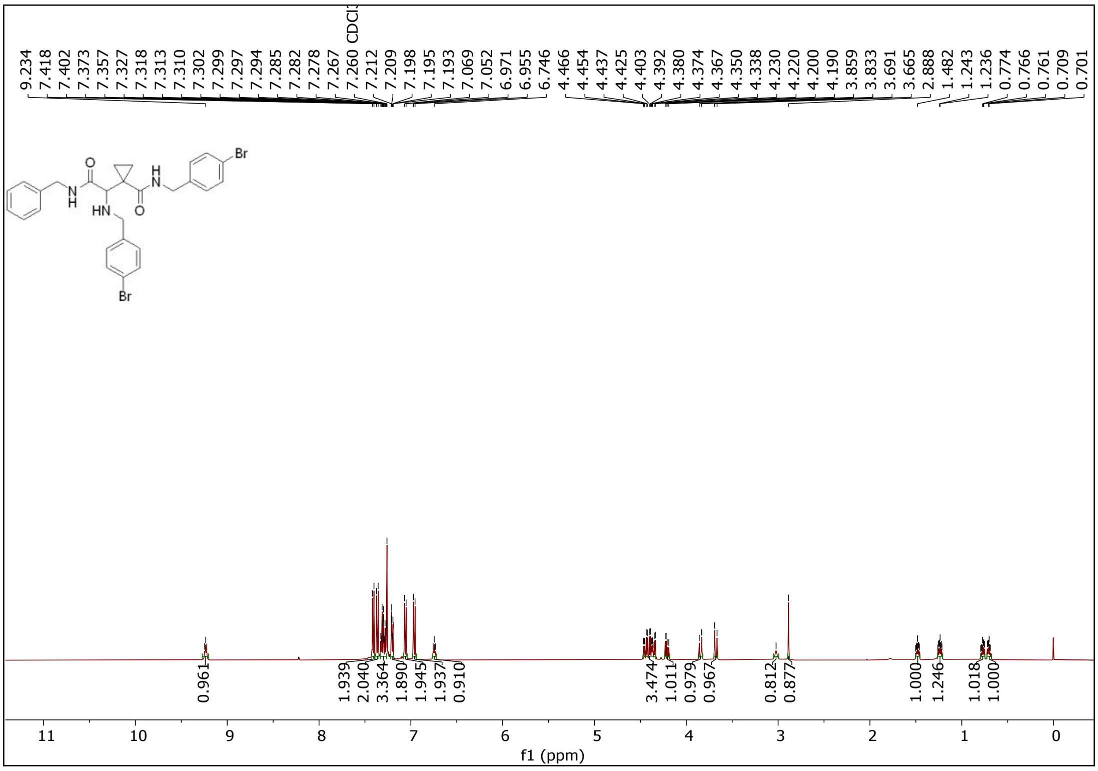

^{13}C NMR spectrum (125 MHz, $CDCl_3$) of compound 16d

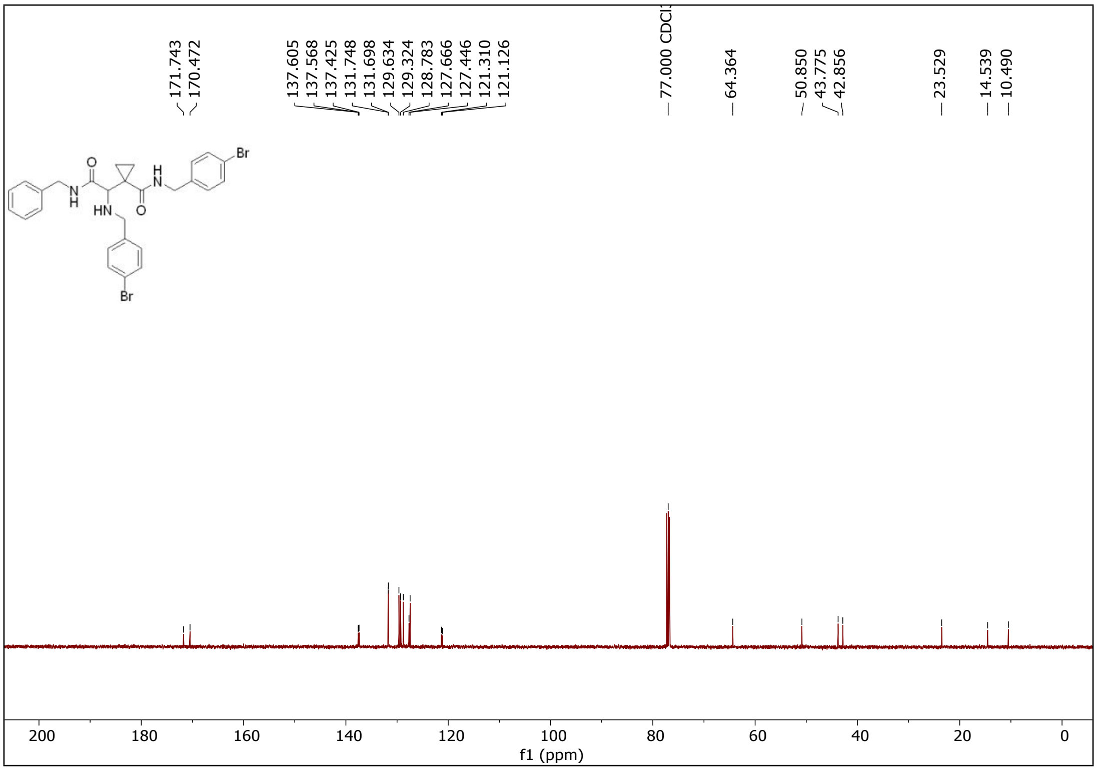

^{1}H NMR spectrum (500 MHz, $CDCl_3$) of compound 16e

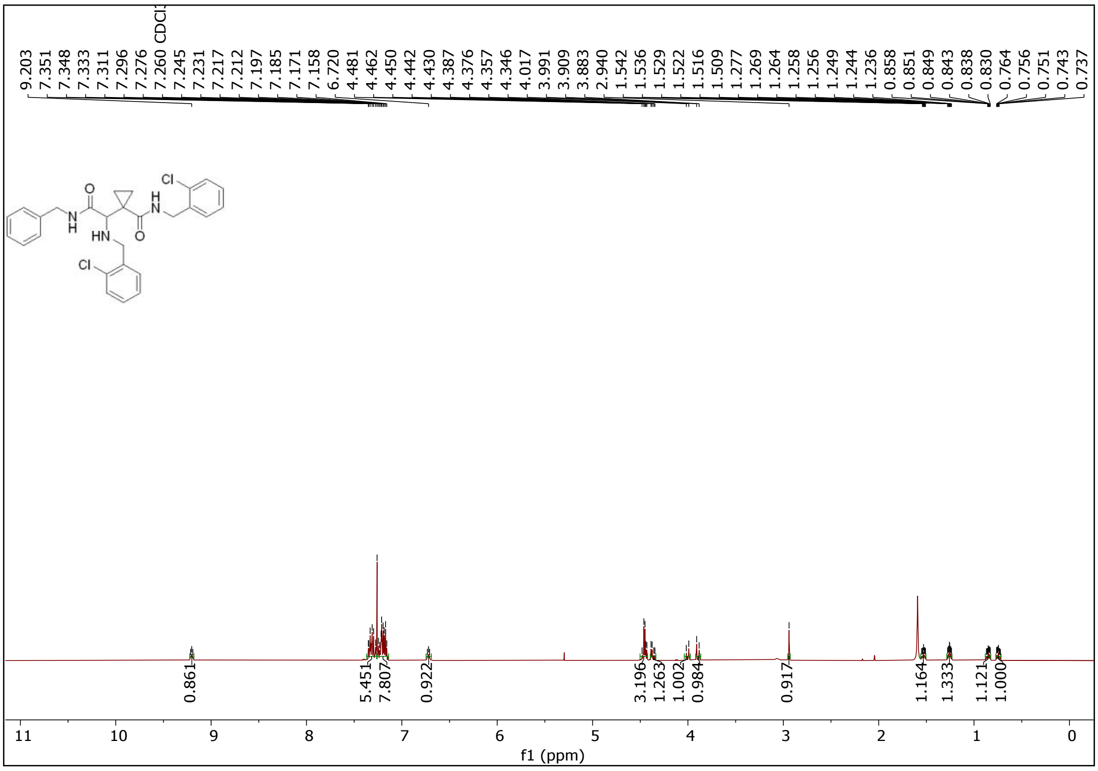

^{13}C NMR spectrum (125 MHz, $CDCl_3$) of compound 16e

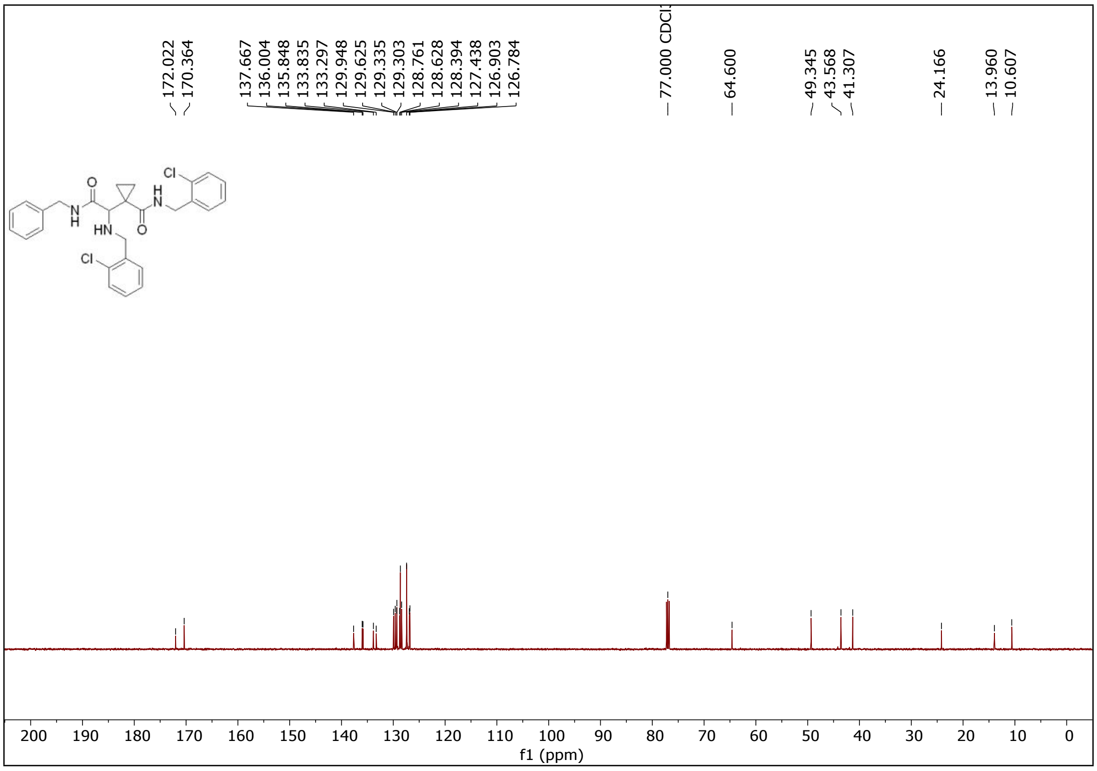

^{1}H NMR spectrum (500 MHz, $CDCl_3$) of compound 16f

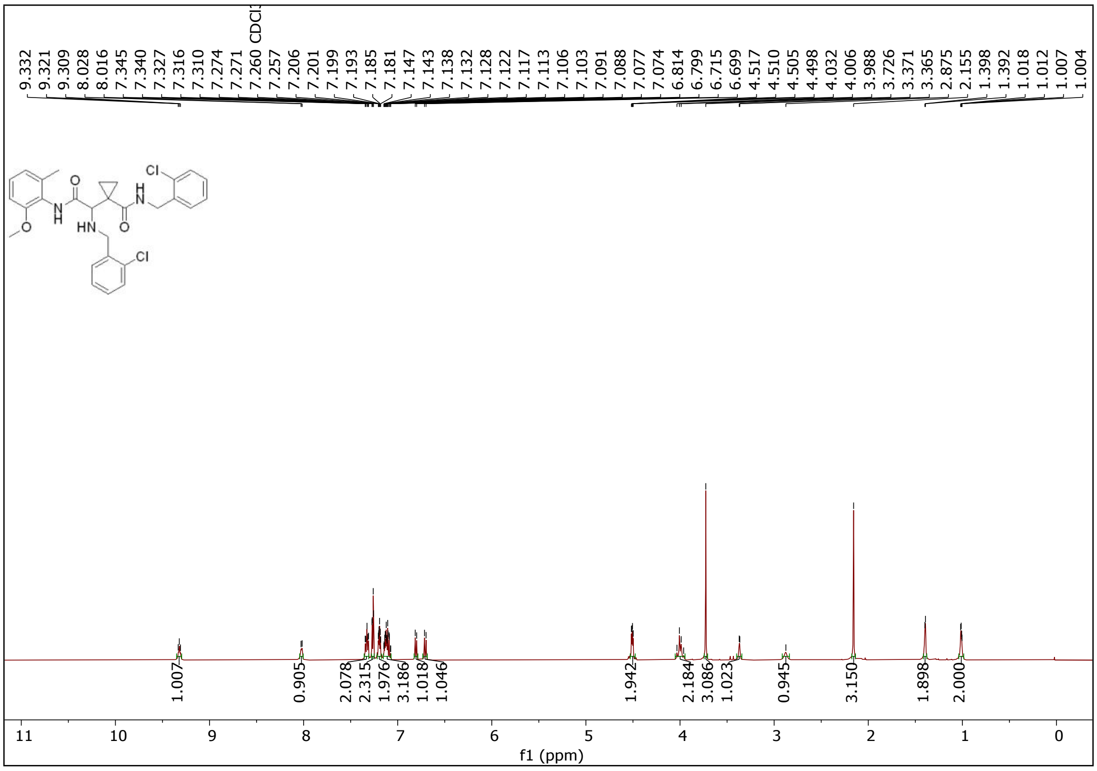

^{13}C NMR spectrum (125 MHz, $CDCl_3$) of compound 16f

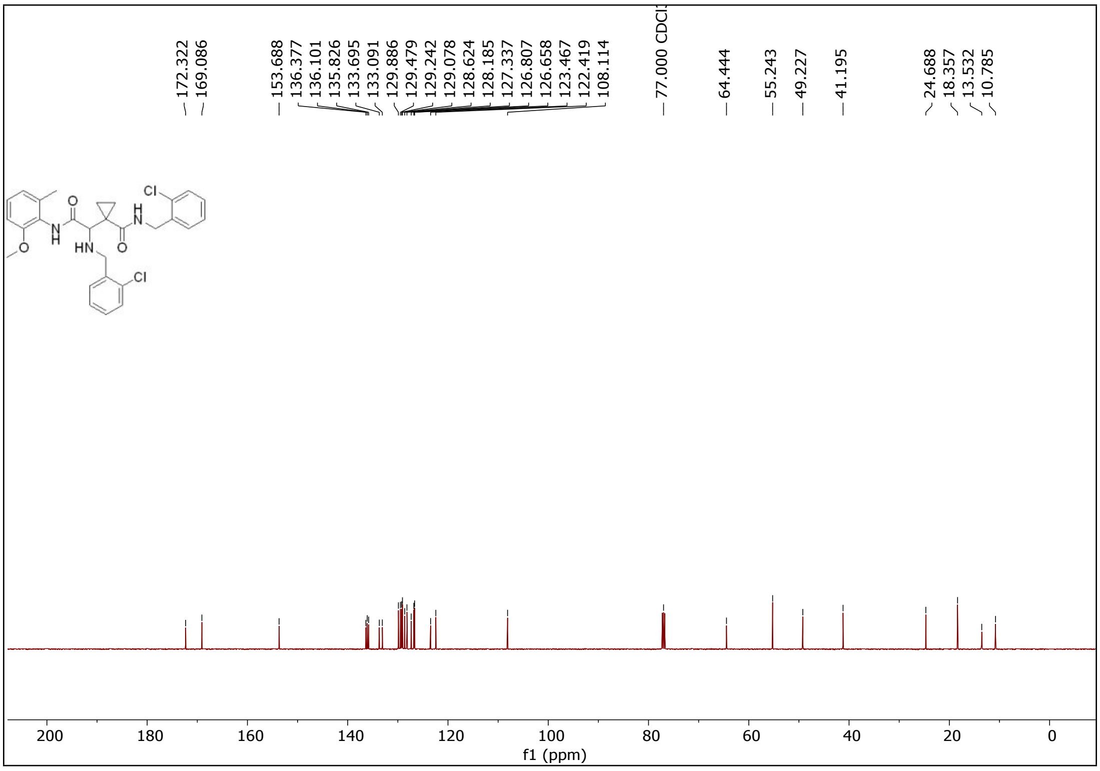

^{1}H NMR spectrum (500 MHz, $CDCl_3$) of compound 16g

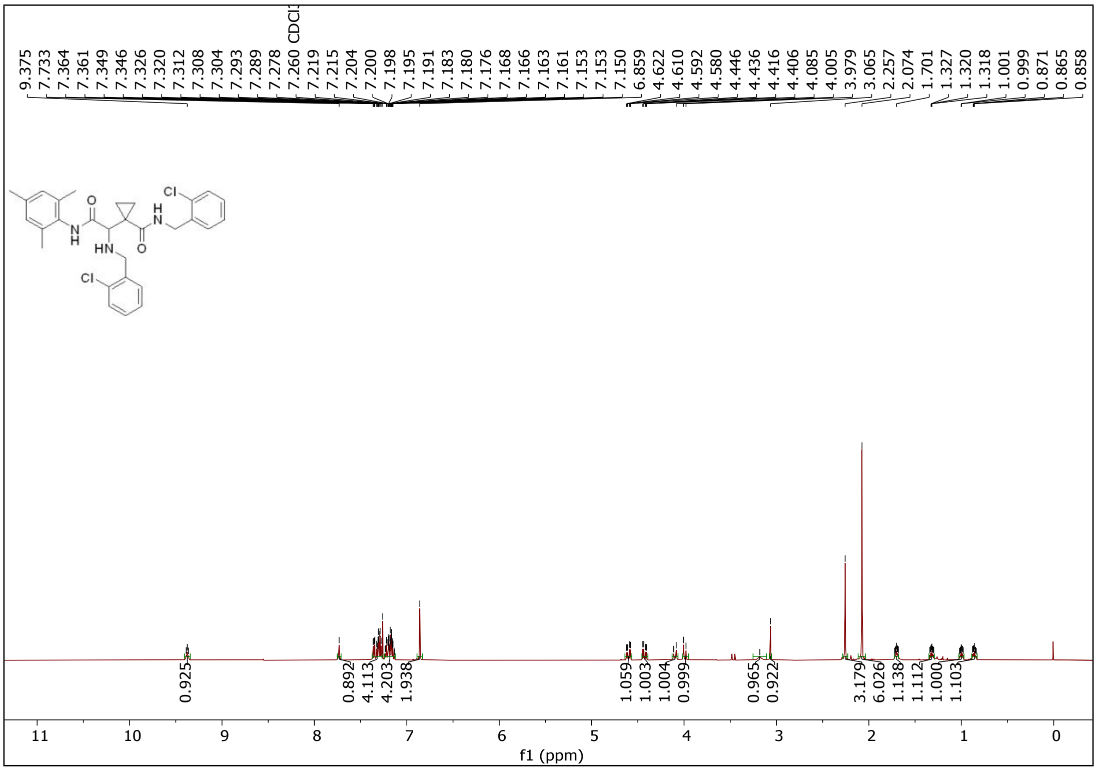

^{13}C NMR spectrum (125 MHz, $CDCl_3$) of compound 16g

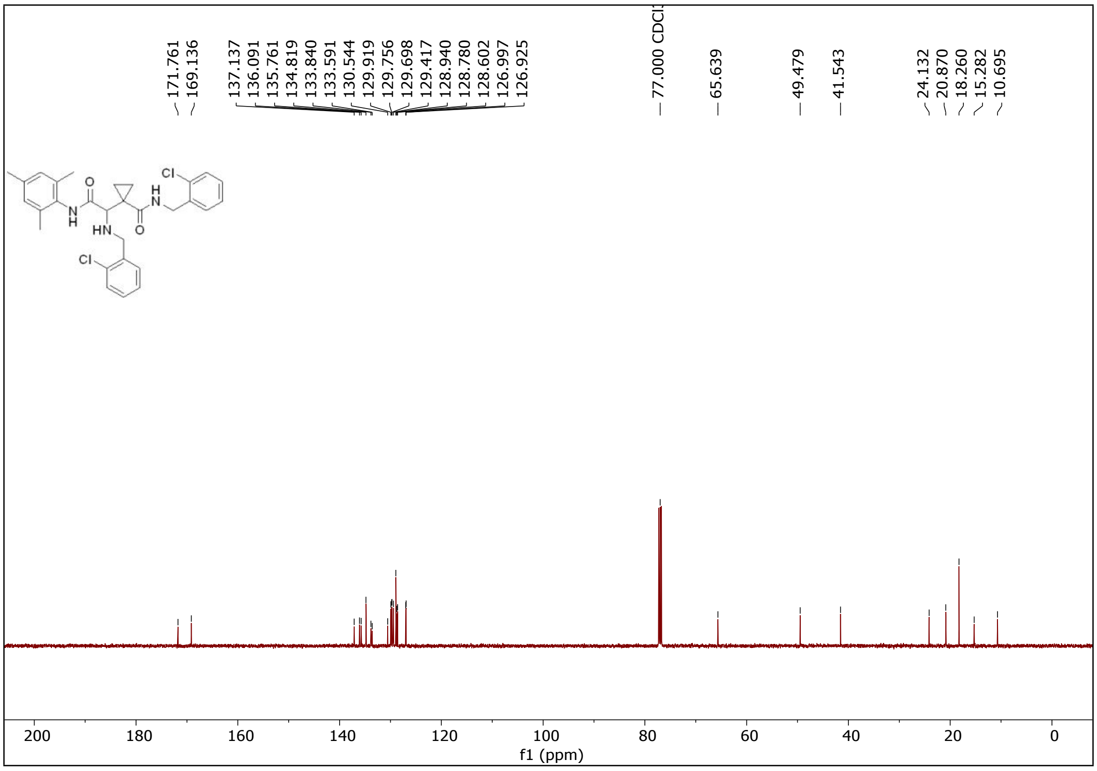

^{1}H NMR spectrum (500 MHz, $CDCl_3$) of compound 16h

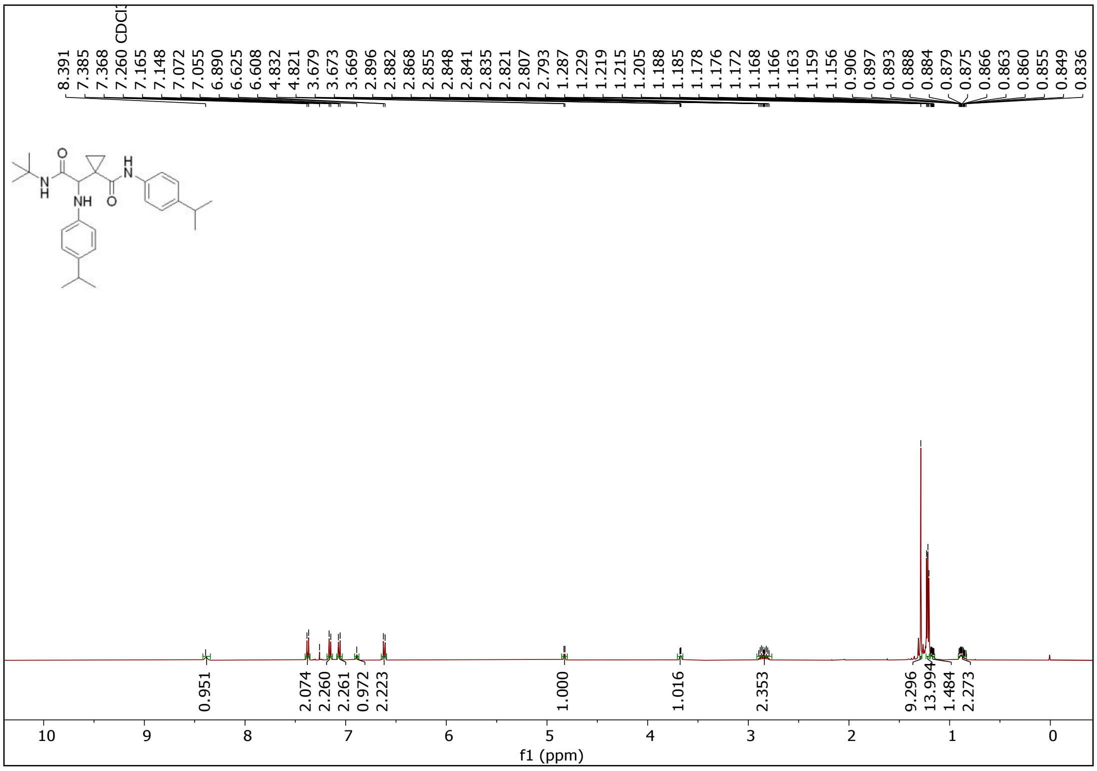

^{13}C NMR spectrum (125 MHz, $CDCl_3$) of compound 16h

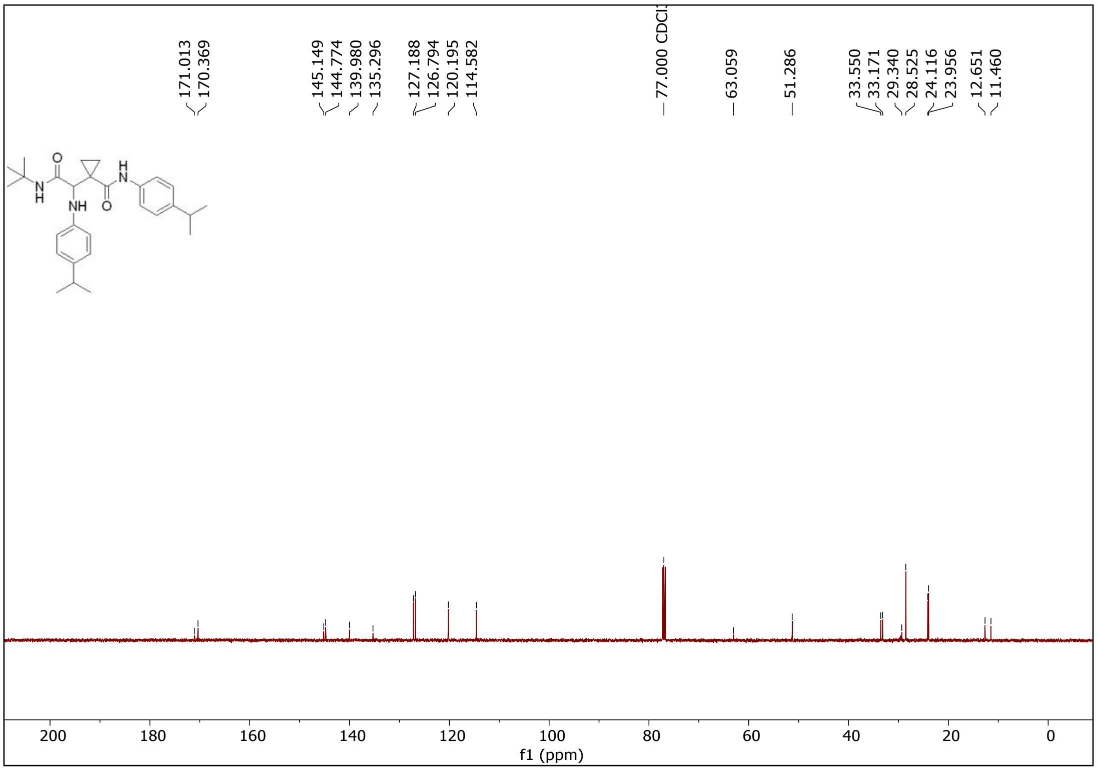

^{1}H NMR spectrum (500 MHz, $CDCl_3$) of compound 16i

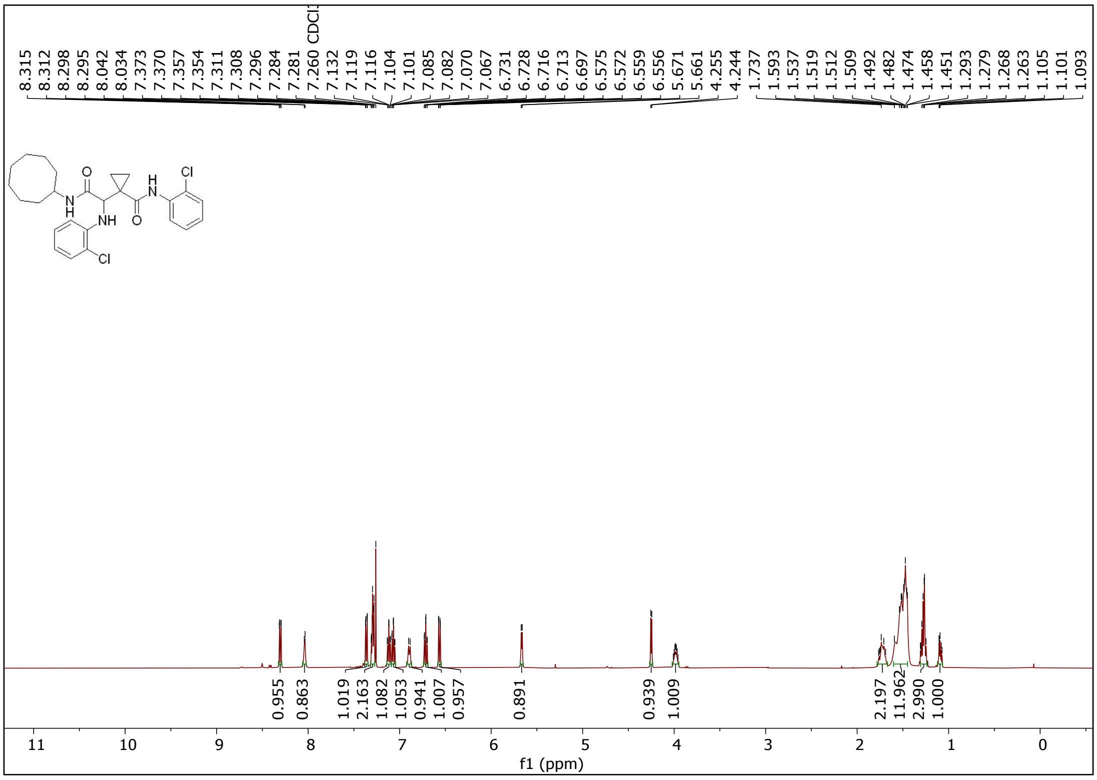

^{13}C NMR spectrum (125 MHz, $CDCl_3$) of compound 16i

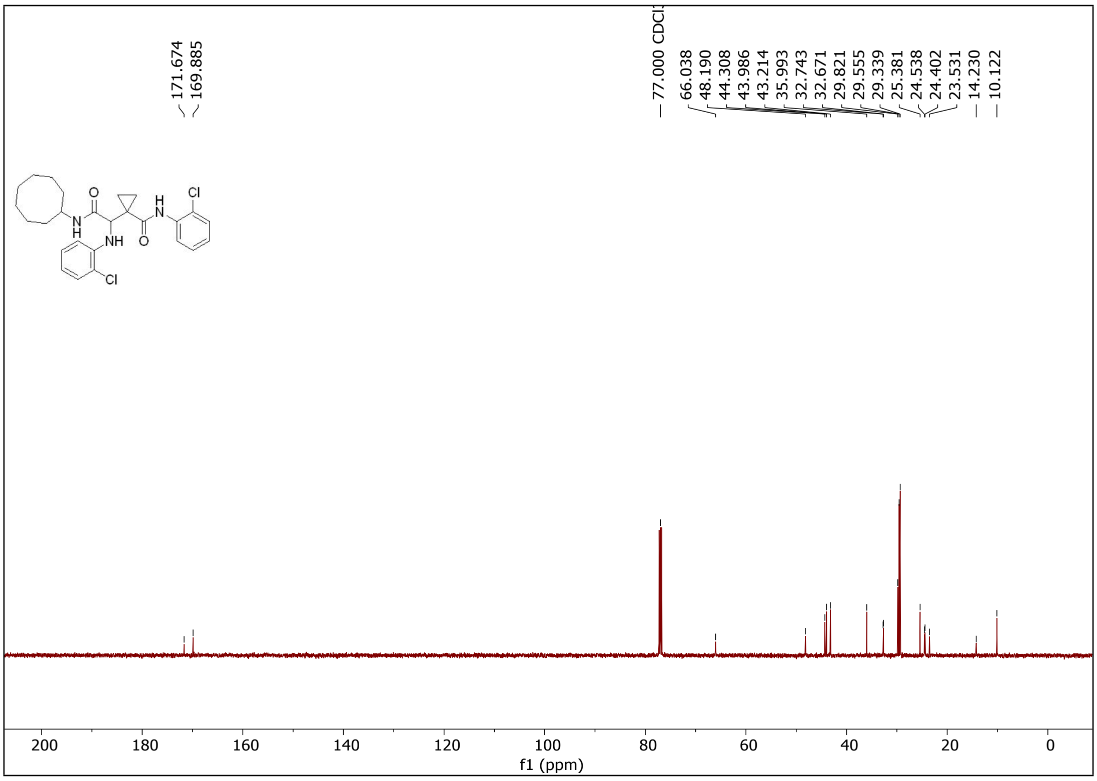

^{1}H NMR spectrum (500 MHz, $CDCl_3$) of compound 16j

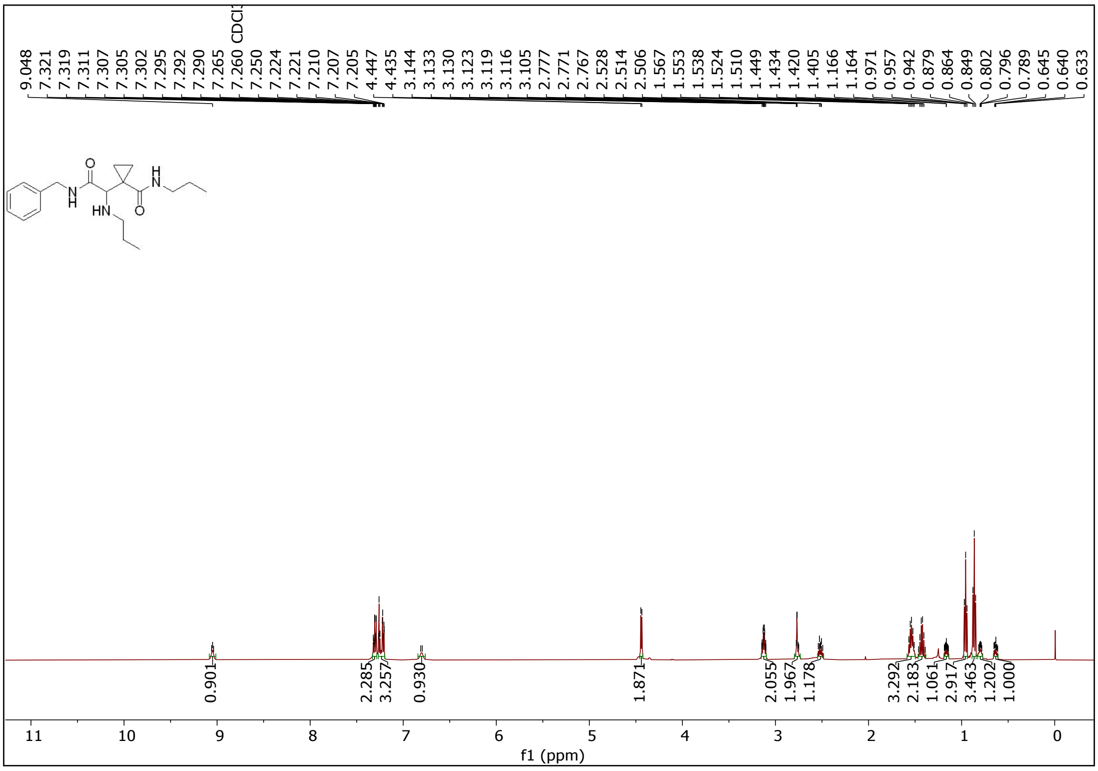

^{13}C NMR spectrum (125 MHz, $CDCl_3$) of compound 16j

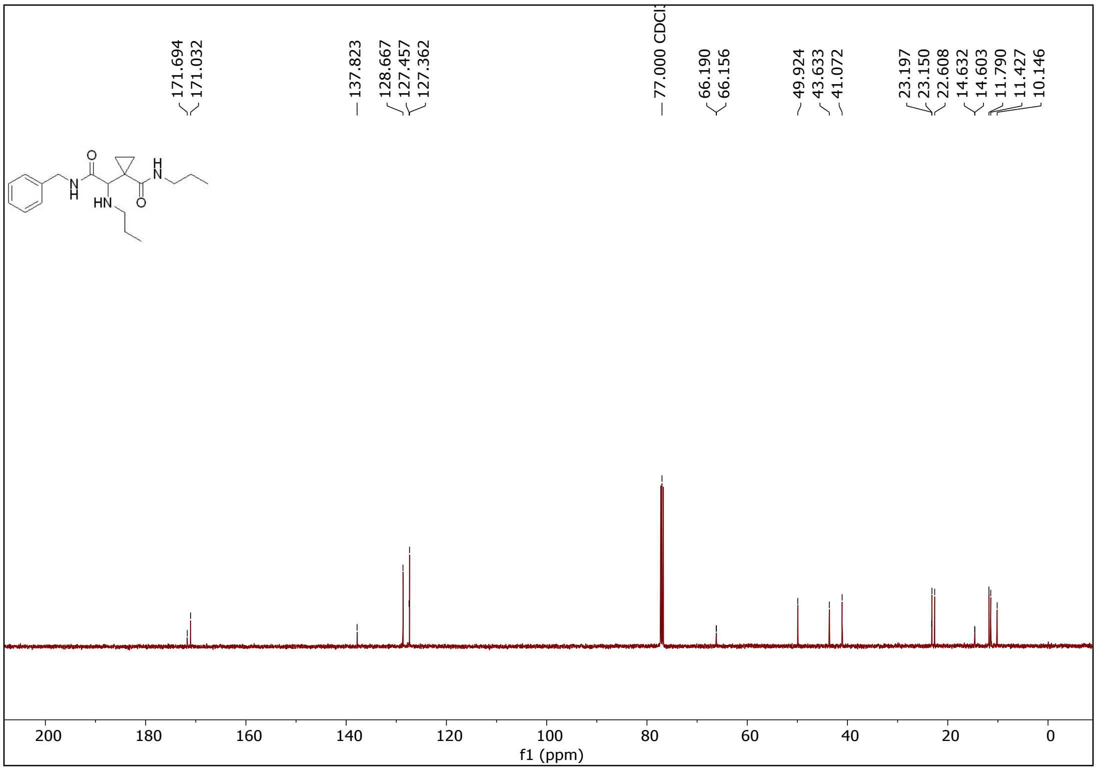

^{1}H NMR spectrum (500 MHz, $CDCl_3$) of compound 16k

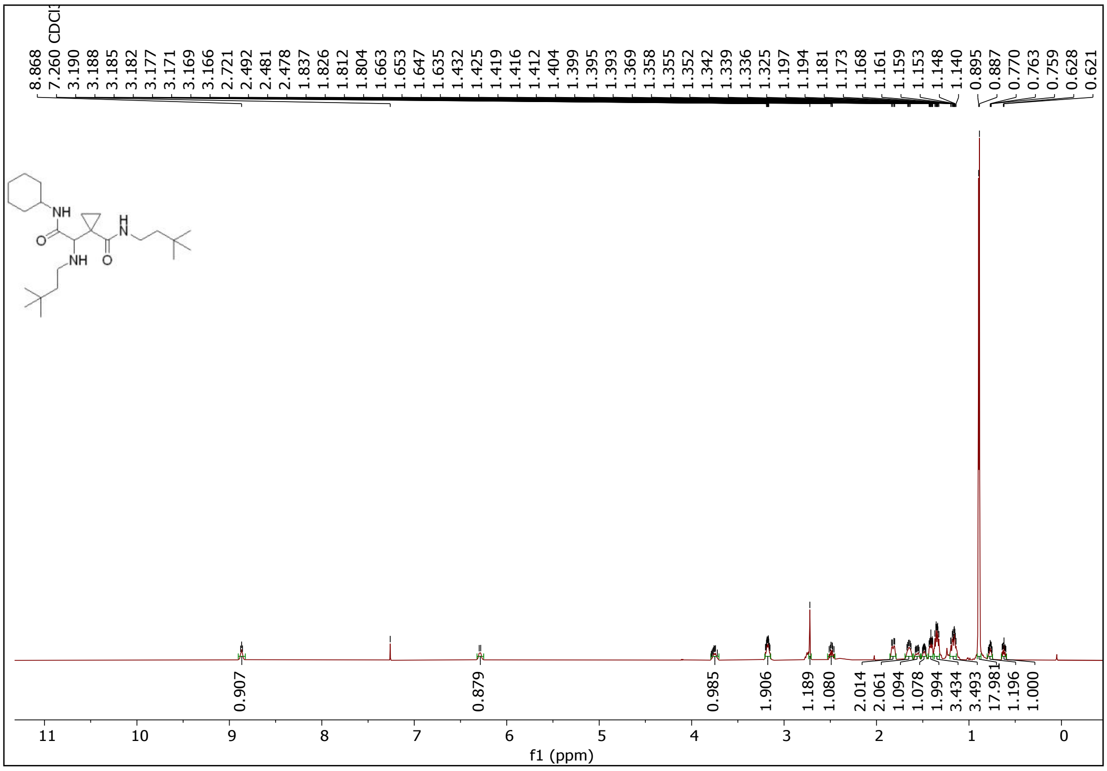

^{13}C NMR spectrum (125 MHz, $CDCl_3$) of compound 16k

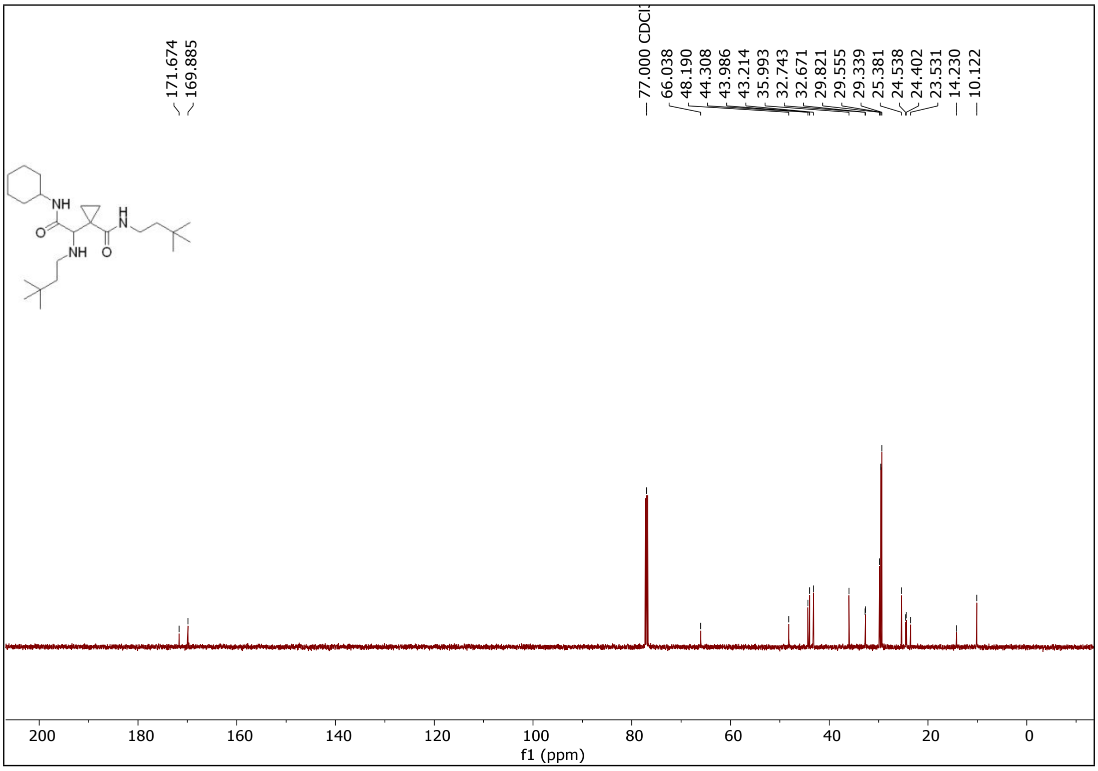

^{1}H NMR spectrum (500 MHz, $CDCl_3$) of compound 16l

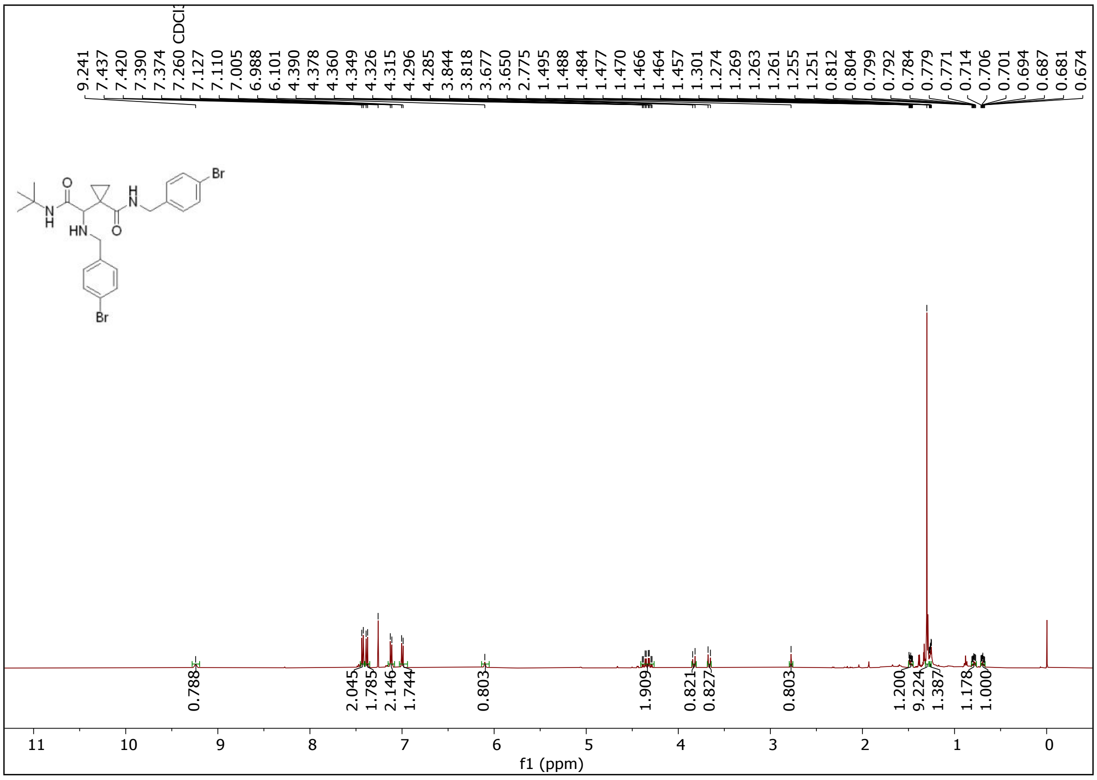

^{13}C NMR spectrum (125 MHz, $CDCl_3$) of compound 16l

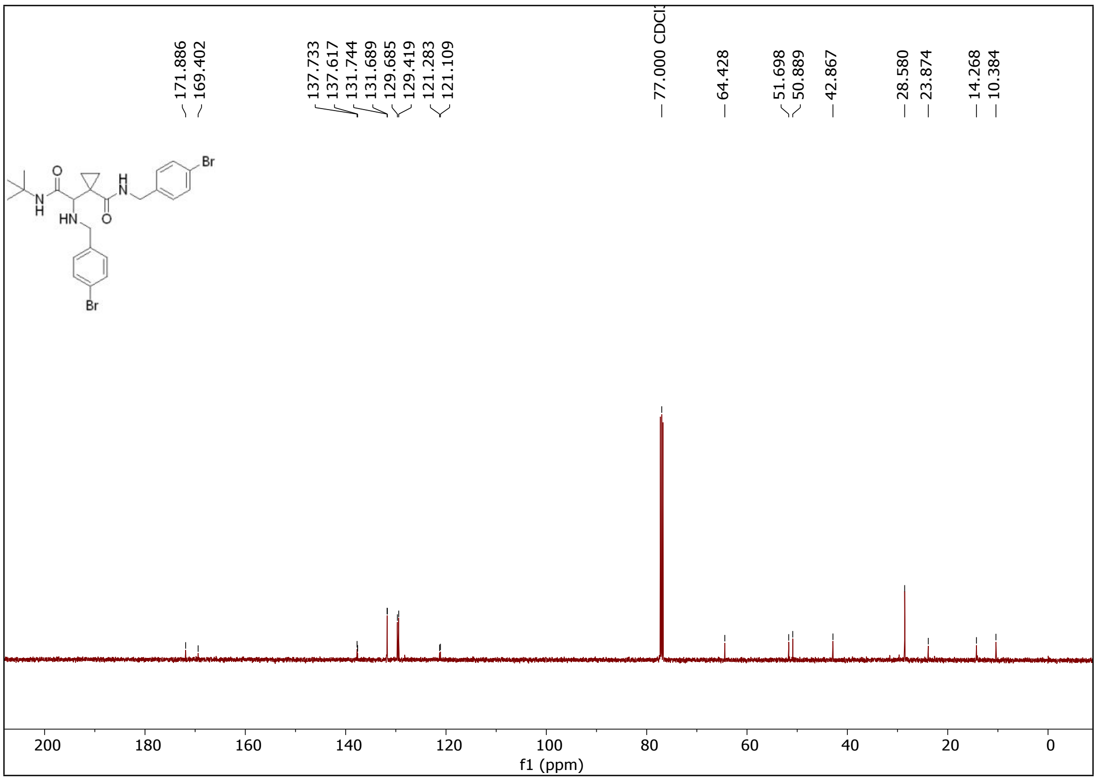

^{1}H NMR spectrum (500 MHz, $CDCl_3$) of compound 16m

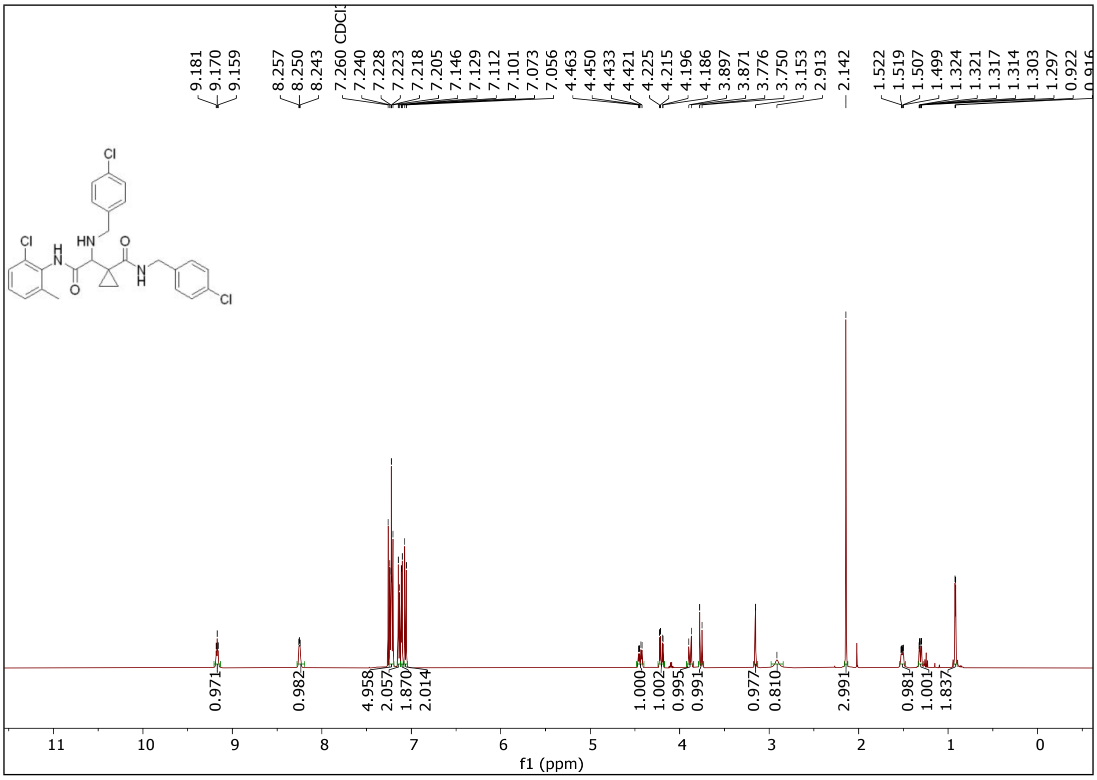

^{13}C NMR spectrum (125 MHz, $CDCl_3$) of compound 16m

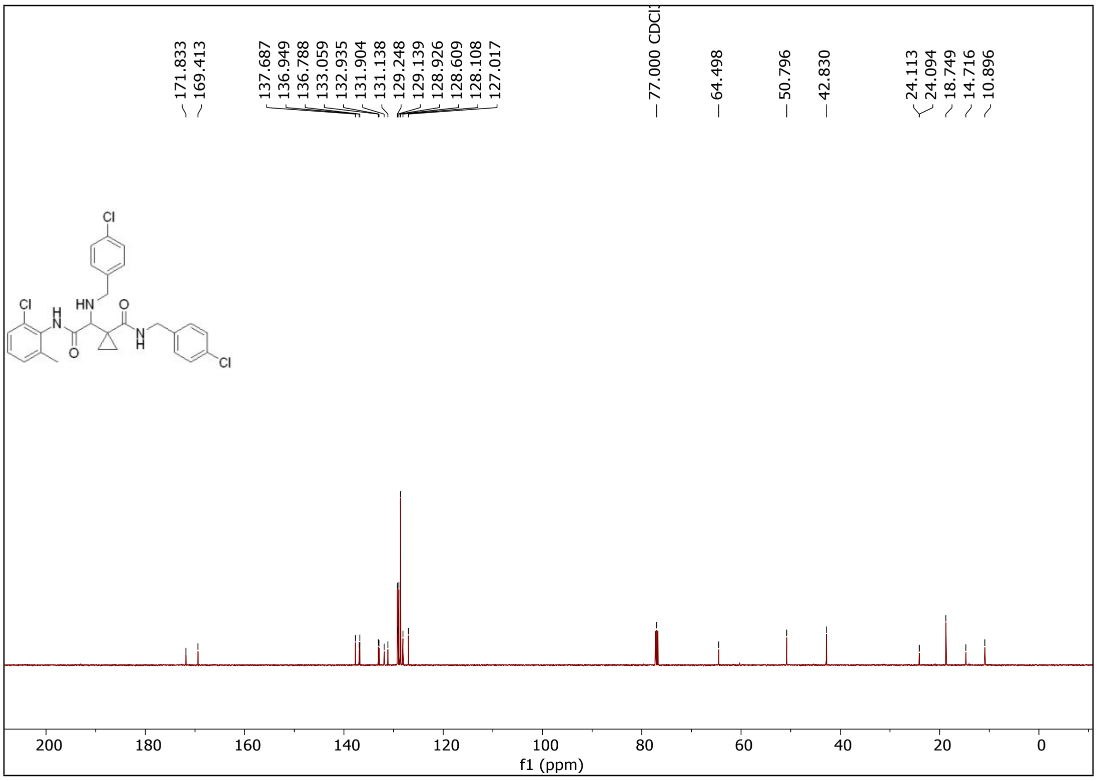

^{1}H NMR spectrum (500 MHz, $CDCl_3$) of compound 16n

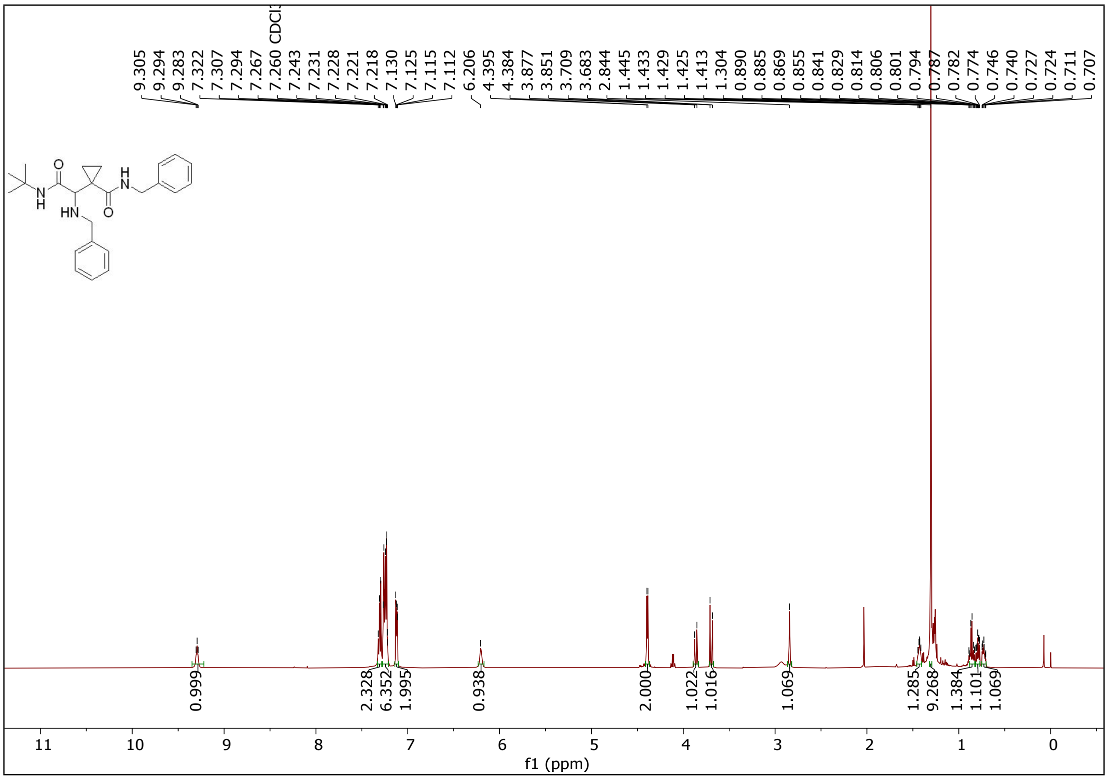

^{13}C NMR spectrum (125 MHz, $CDCl_3$) of compound 16n

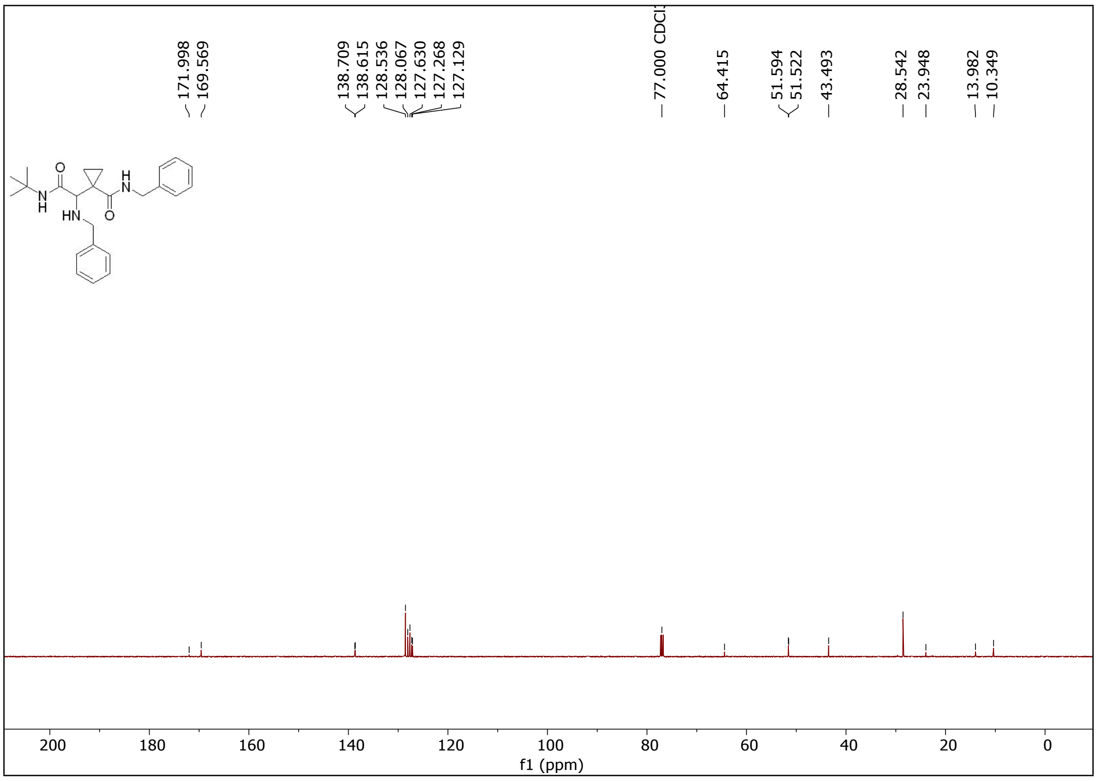

^{1}H NMR spectrum (500 MHz, $CDCl_3$) of compound 17a

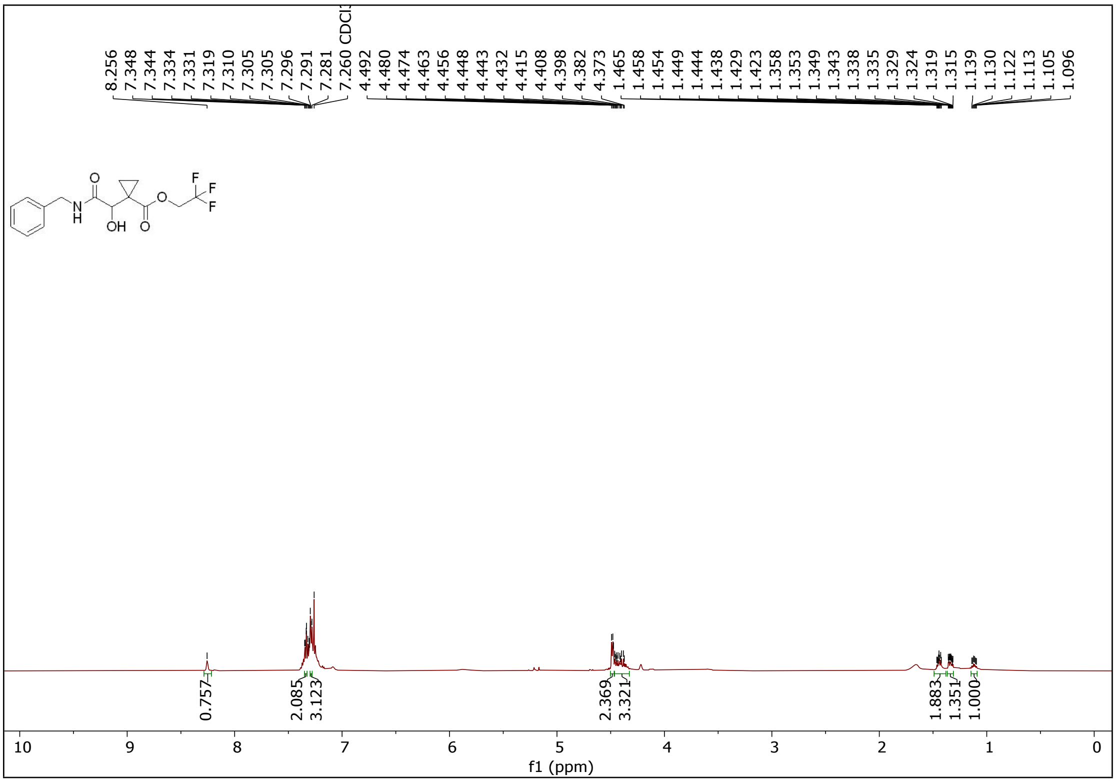

^{13}C NMR spectrum (125 MHz, $CDCl_3$) of compound 17a

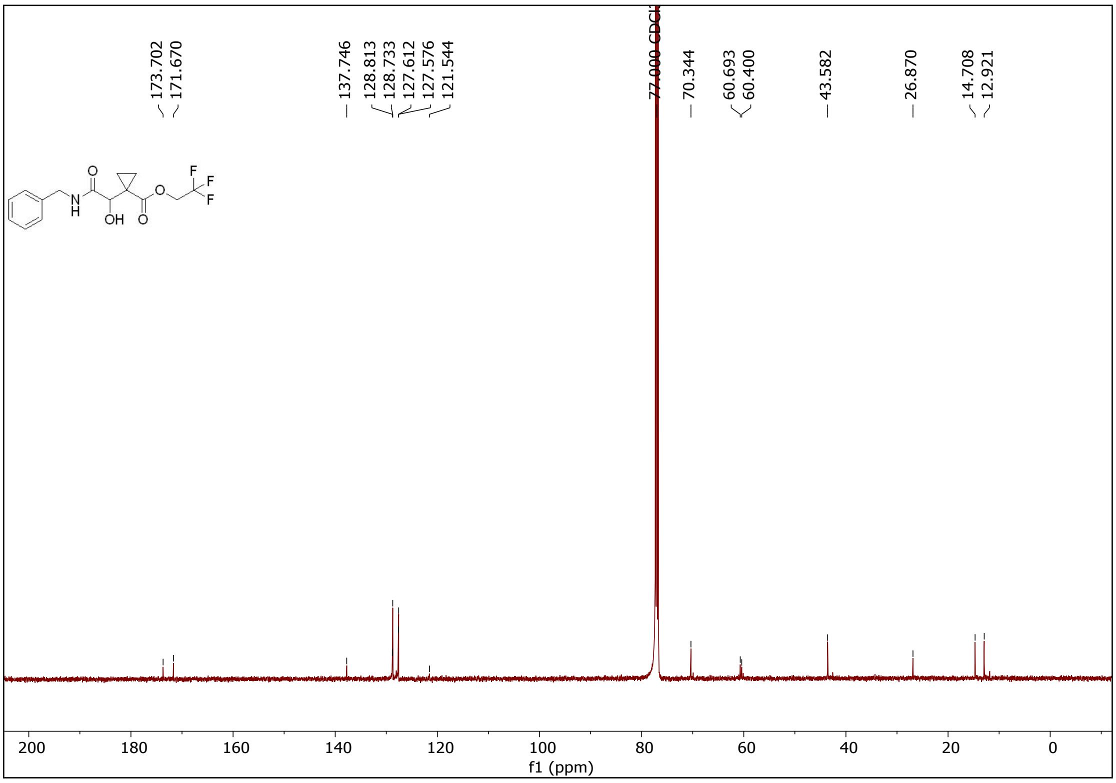

^{1}H NMR spectrum (500 MHz, $CDCl_3$) of compound 17b

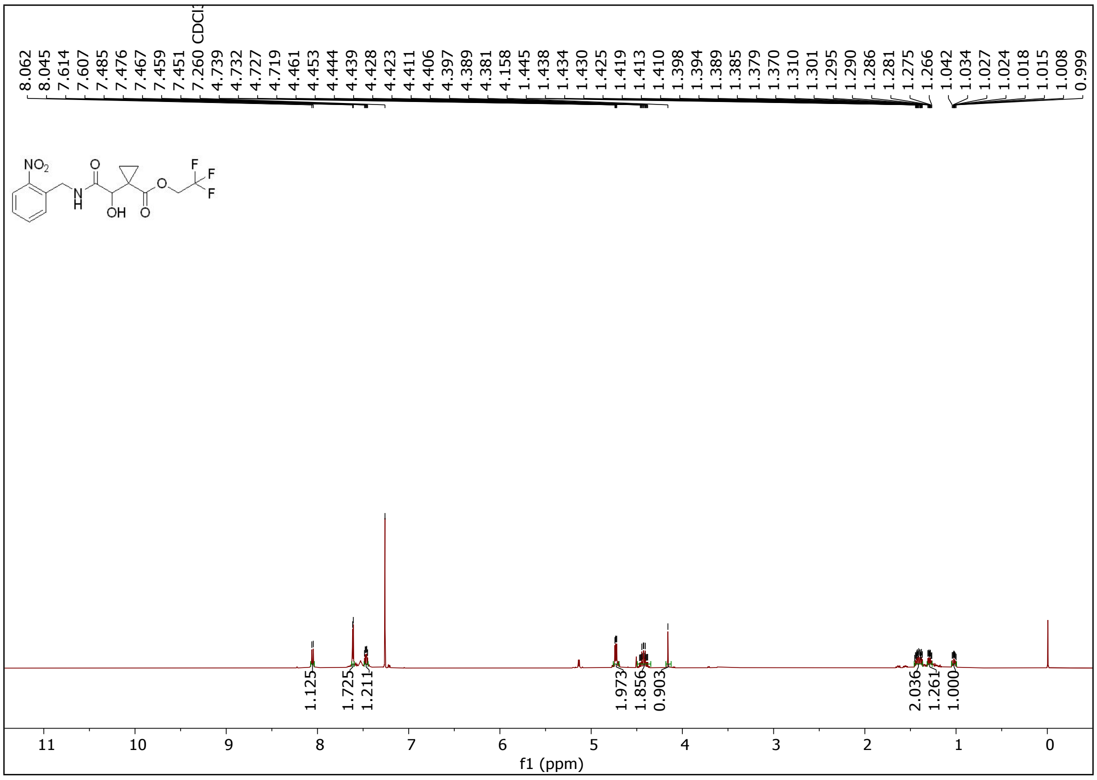

^{13}C NMR spectrum (125 MHz, $CDCl_3$) of compound 17b

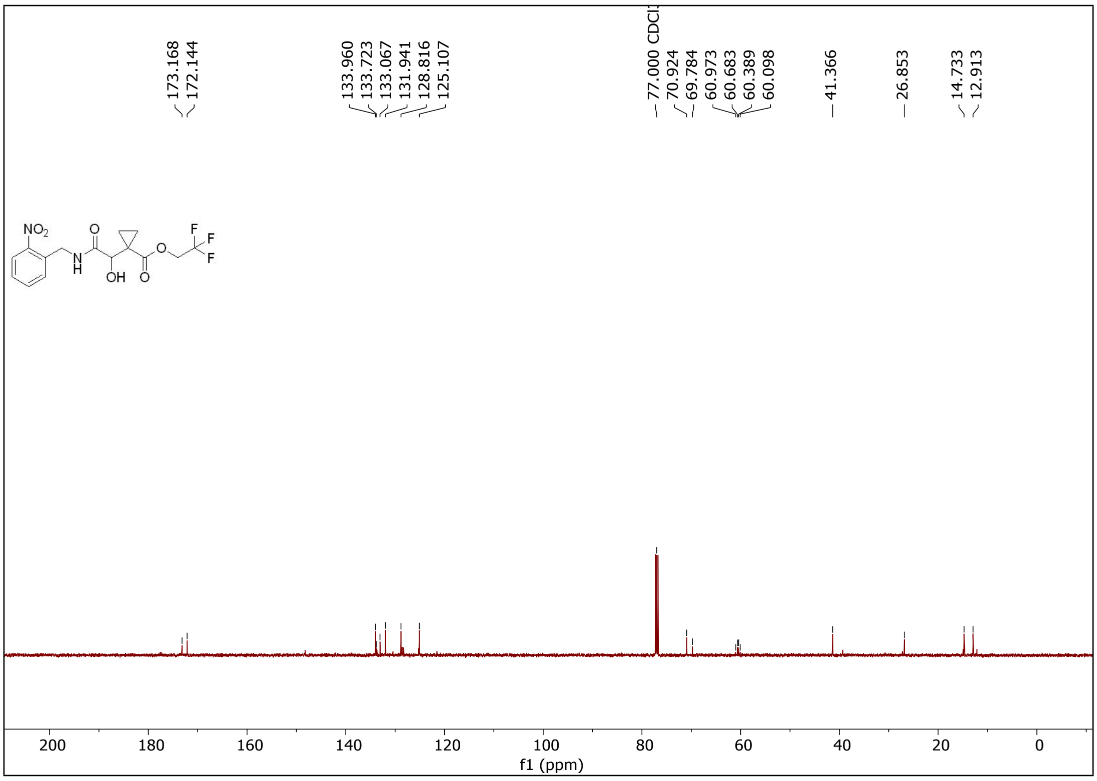

^{1}H NMR spectrum (500 MHz, $CDCl_3$) of compound 17c

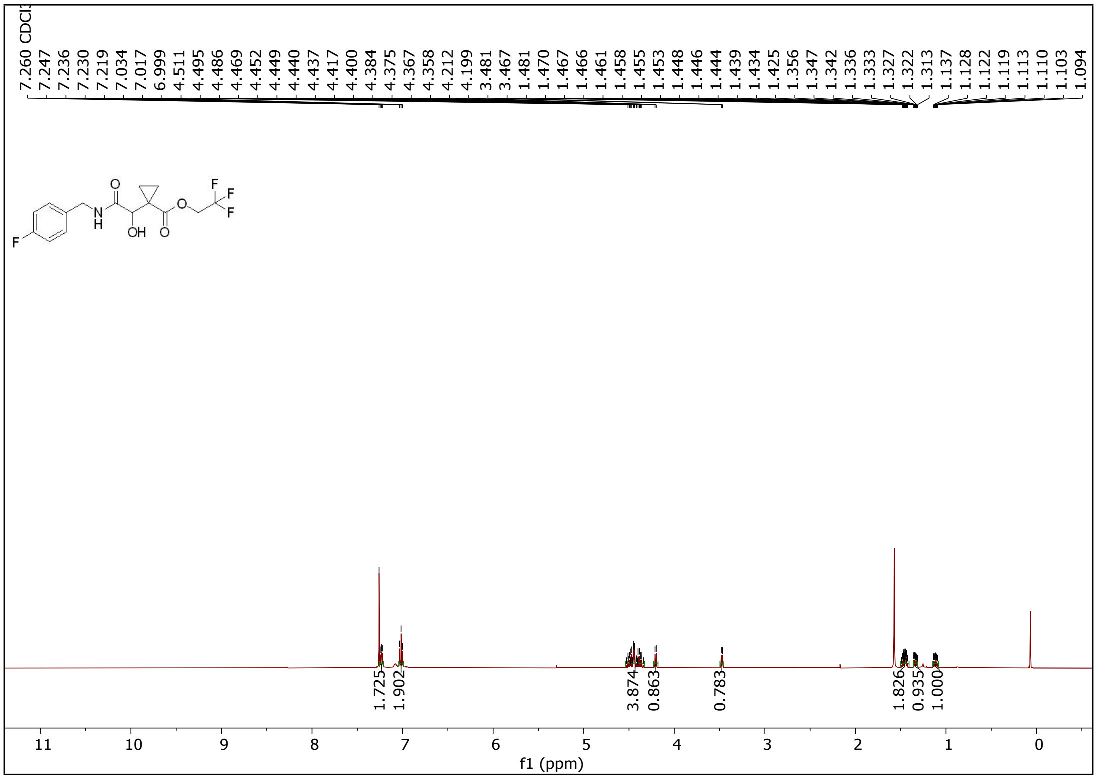

^{13}C NMR spectrum (125 MHz, $CDCl_3$) of compound 17c

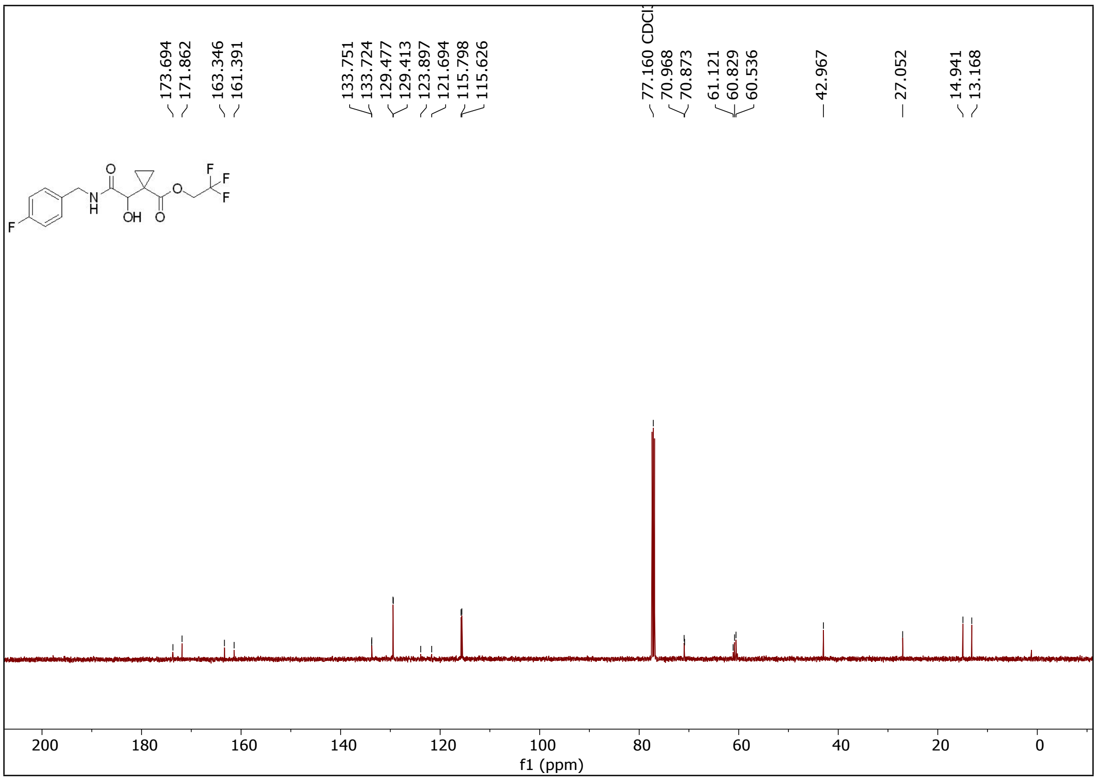

^{1}H NMR spectrum (500 MHz, $CDCl_3$) of compound 17d

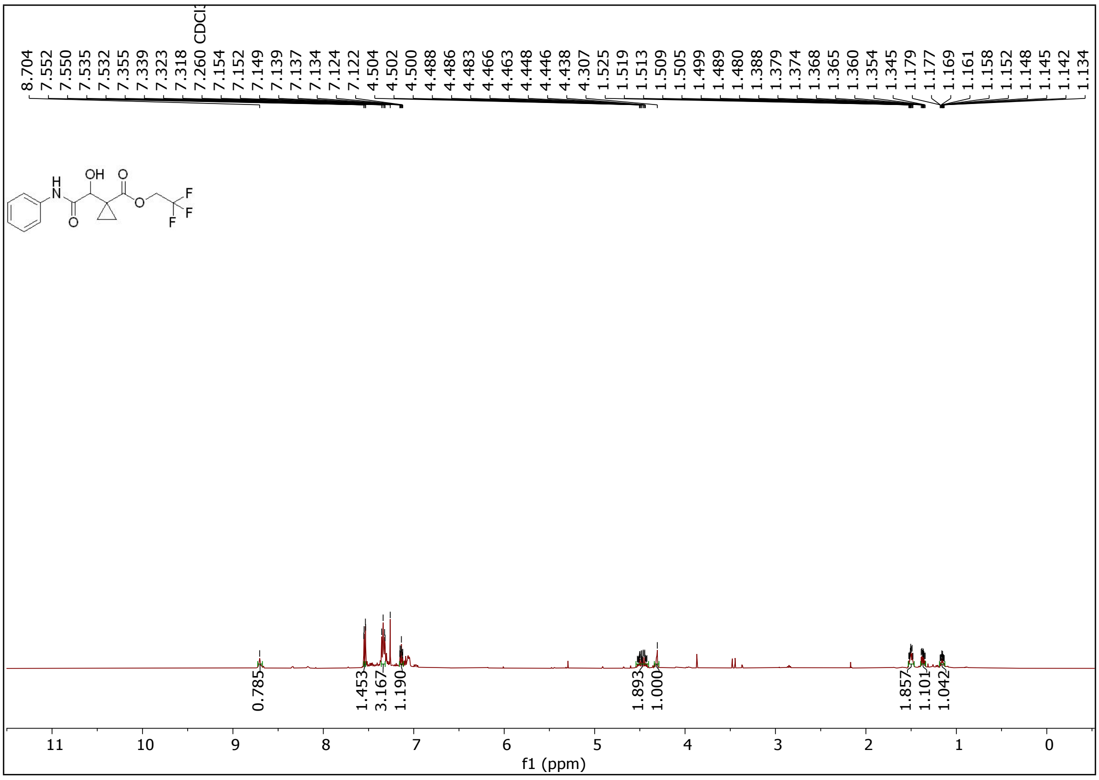

^{13}C NMR spectrum (125 MHz, $CDCl_3$) of compound 17d

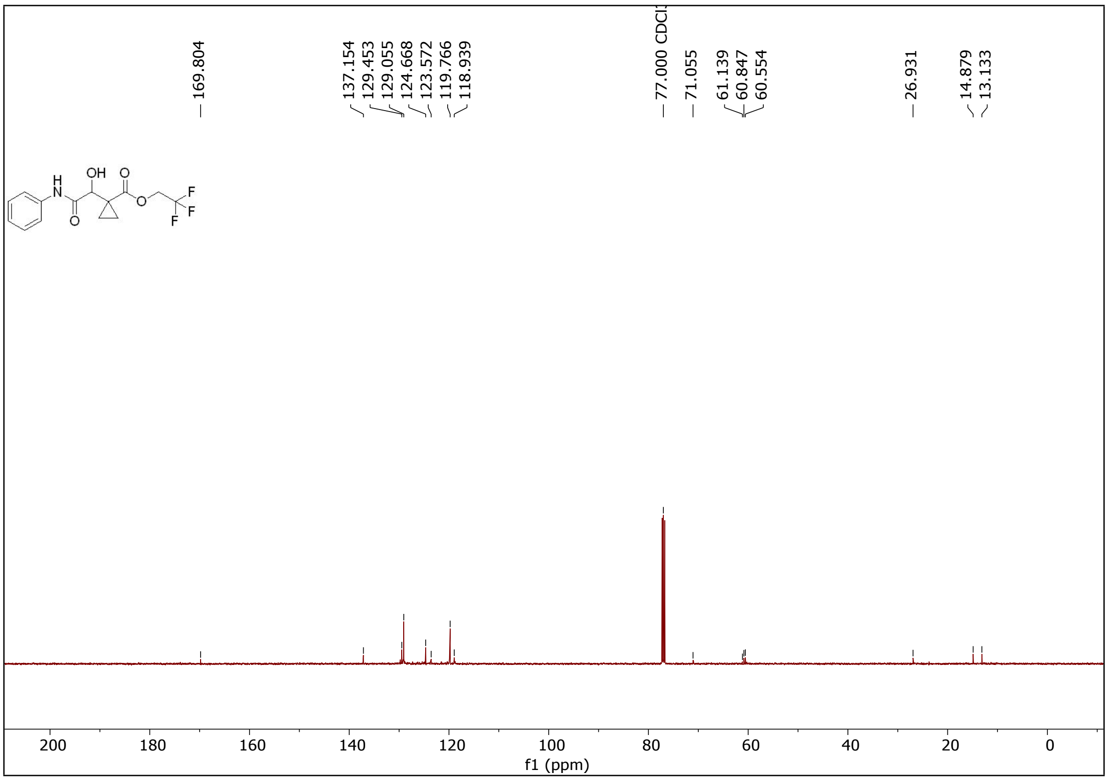

^{1}H NMR spectrum (500 MHz, $CDCl_3$) of compound 17e

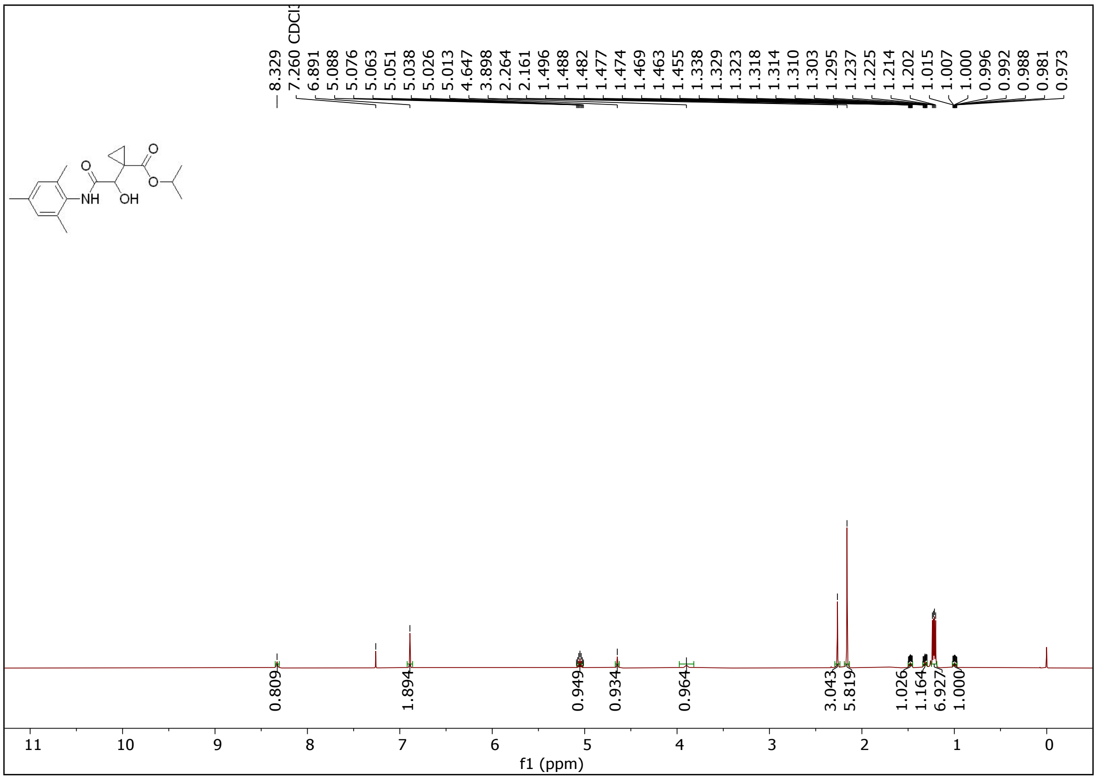

^{13}C NMR spectrum (125 MHz, $CDCl_3$) of compound 17e

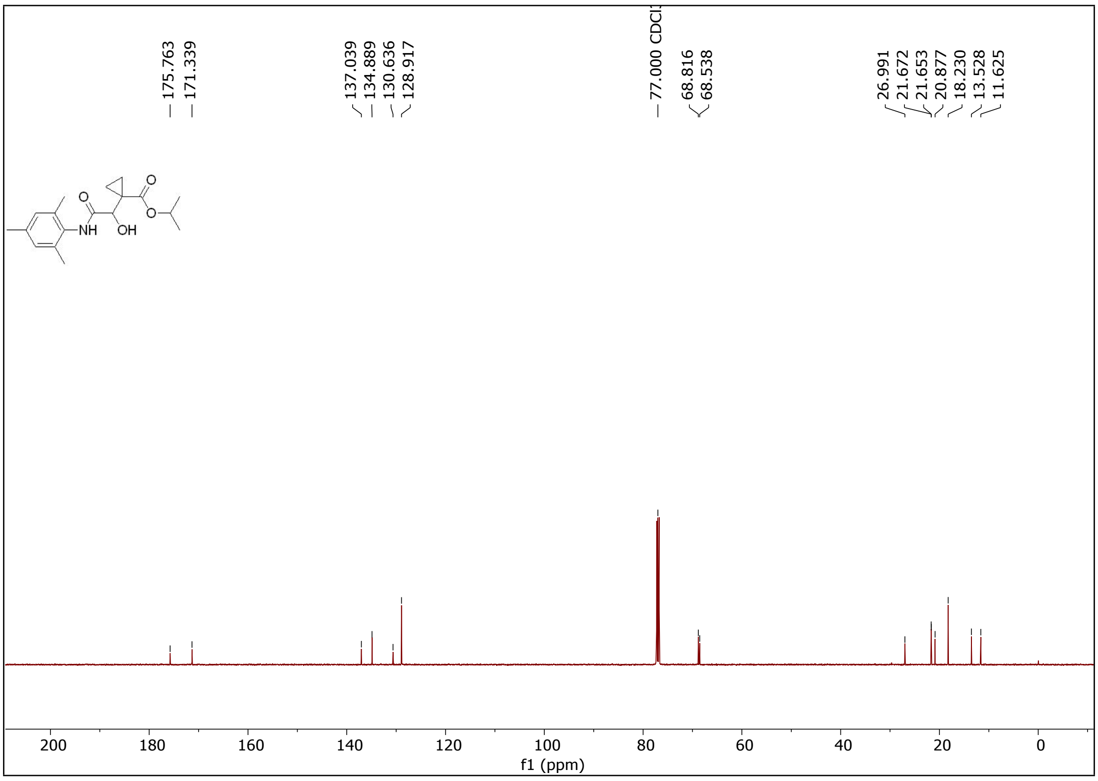

^{1}H NMR spectrum (500 MHz, $CDCl_3$) of compound 17f

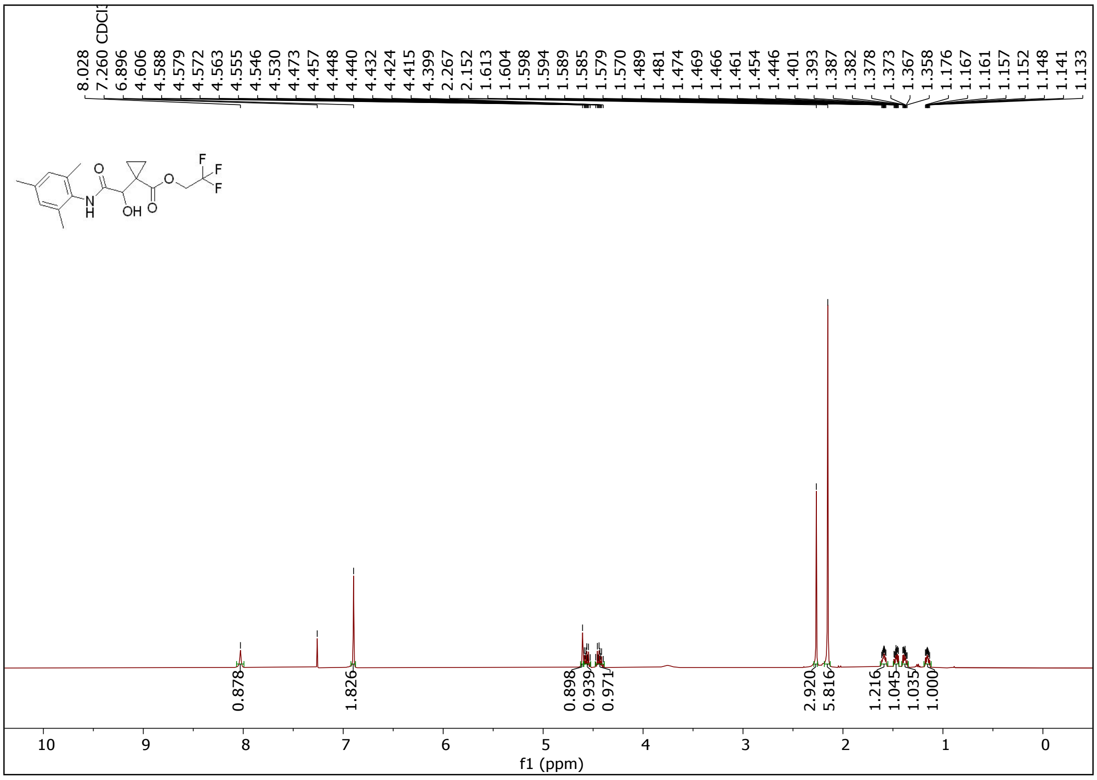

^{13}C NMR spectrum (125 MHz, $CDCl_3$) of compound 17f

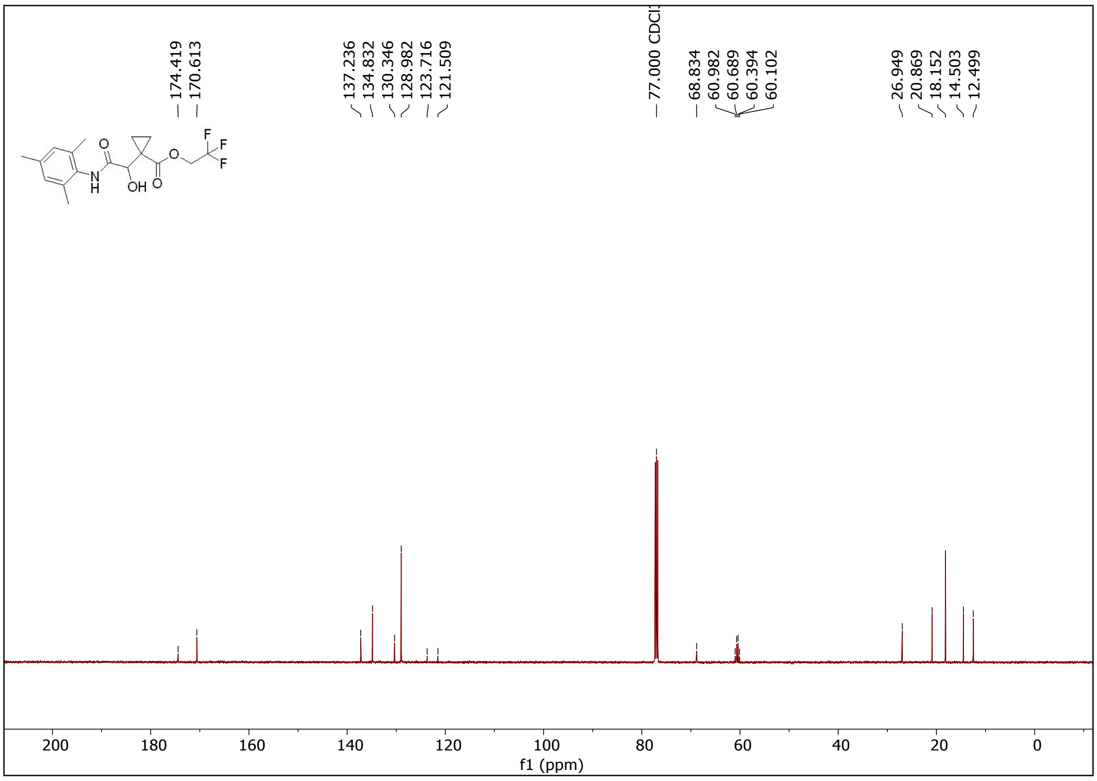

^{1}H NMR spectrum (500 MHz, $CDCl_3$) of compound 17g

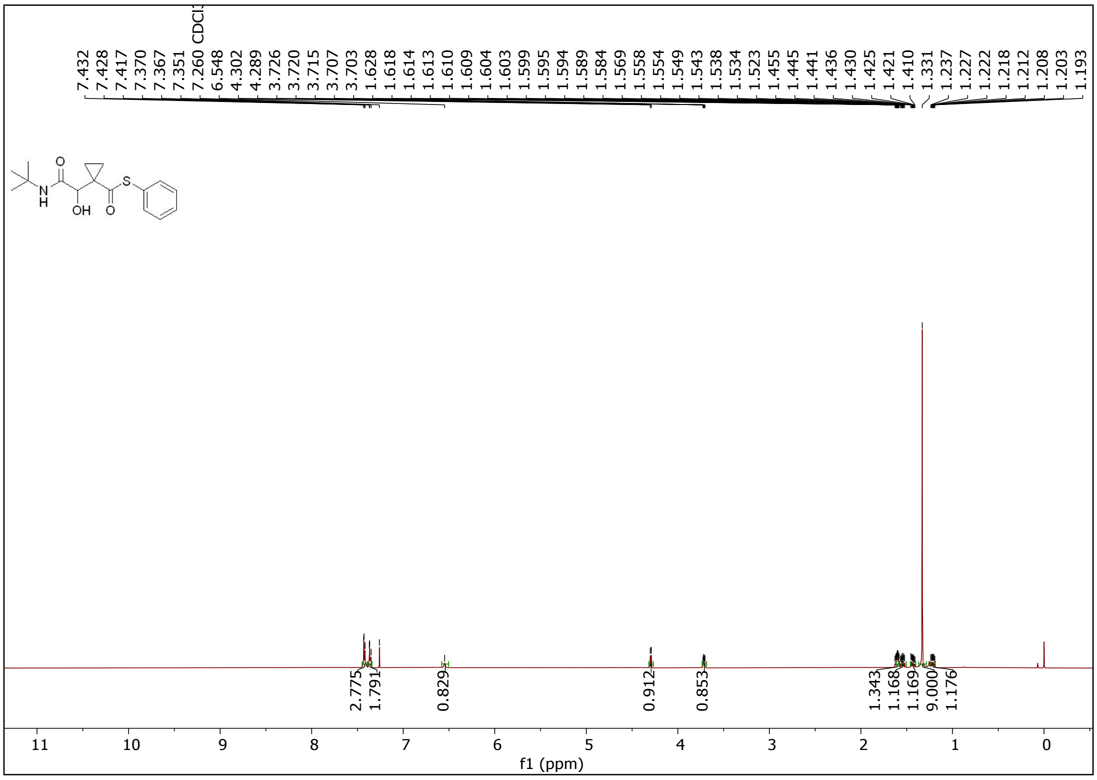

^{13}C NMR spectrum (125 MHz, $CDCl_3$) of compound 17g

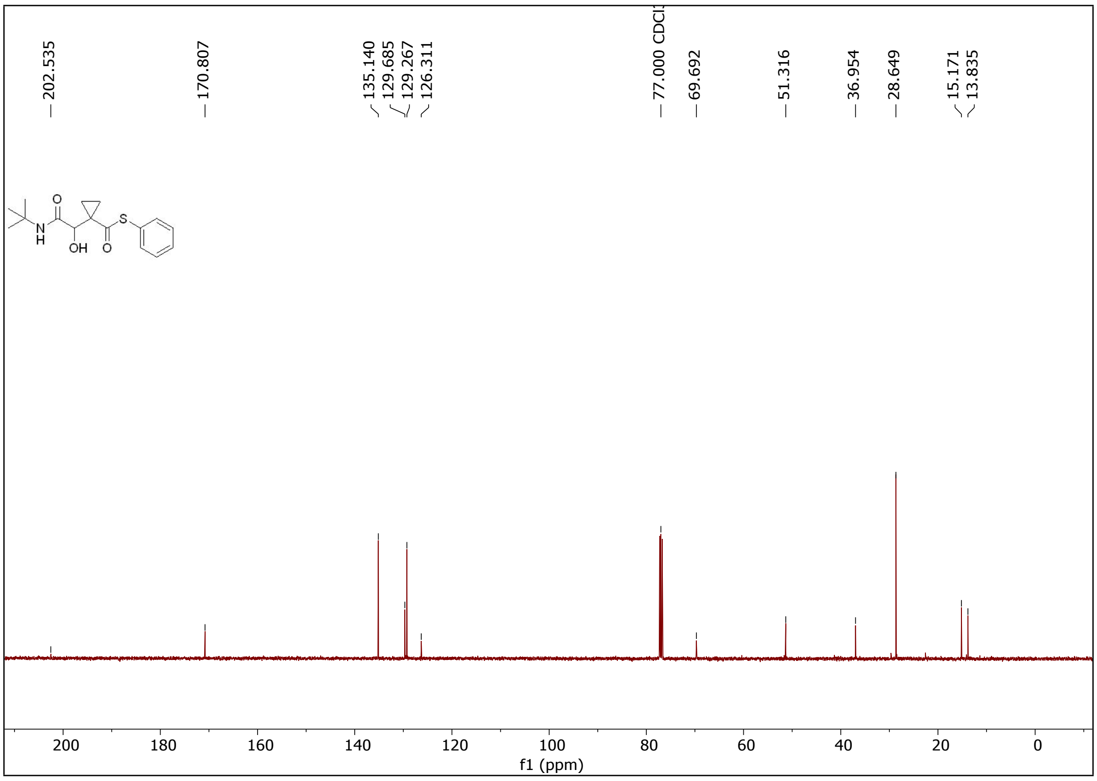

^{1}H NMR spectrum (500 MHz, $CDCl_3$) of compound 17h

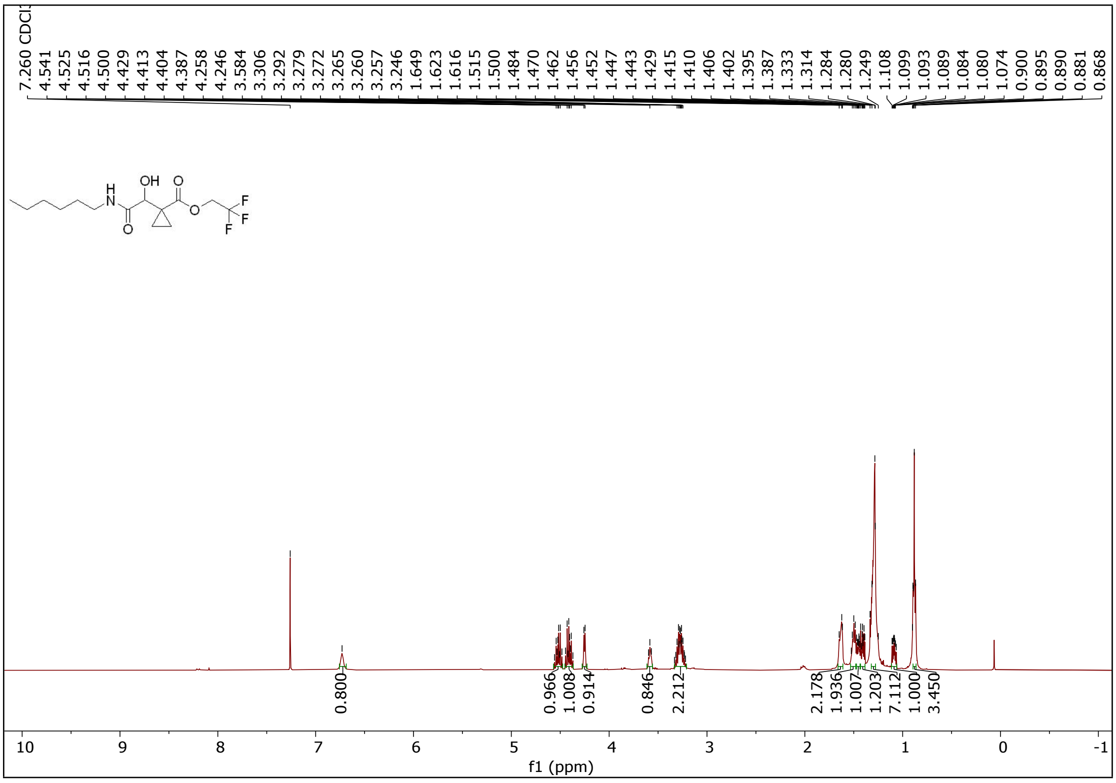

^{13}C NMR spectrum (125 MHz, $CDCl_3$) of compound 17h

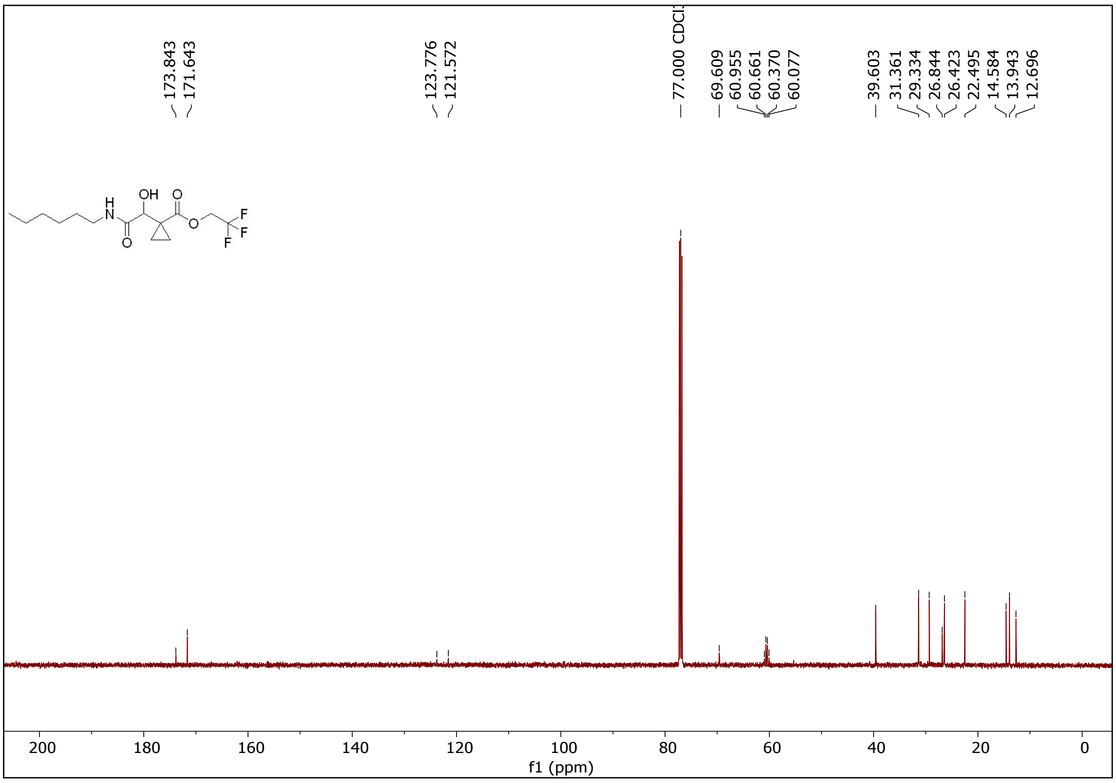

^{1}H NMR spectrum (500 MHz, $CDCl_3$) of compound 17i

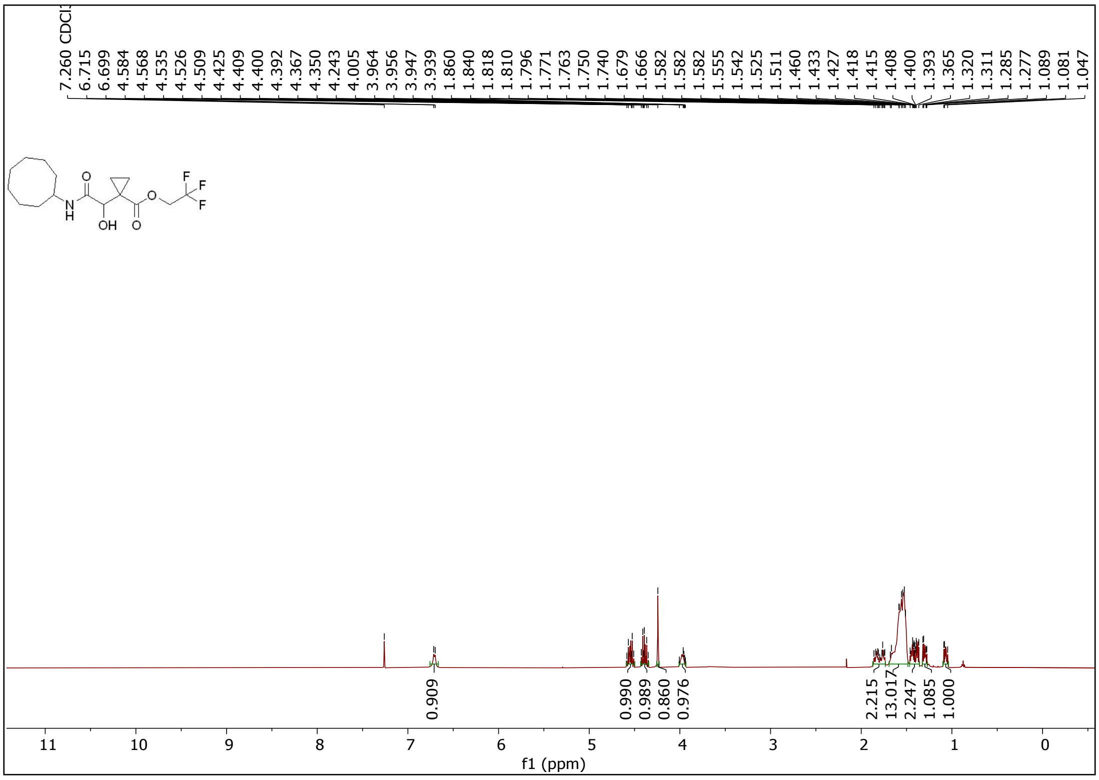

^{13}C NMR spectrum (125 MHz, $CDCl_3$) of compound 17i

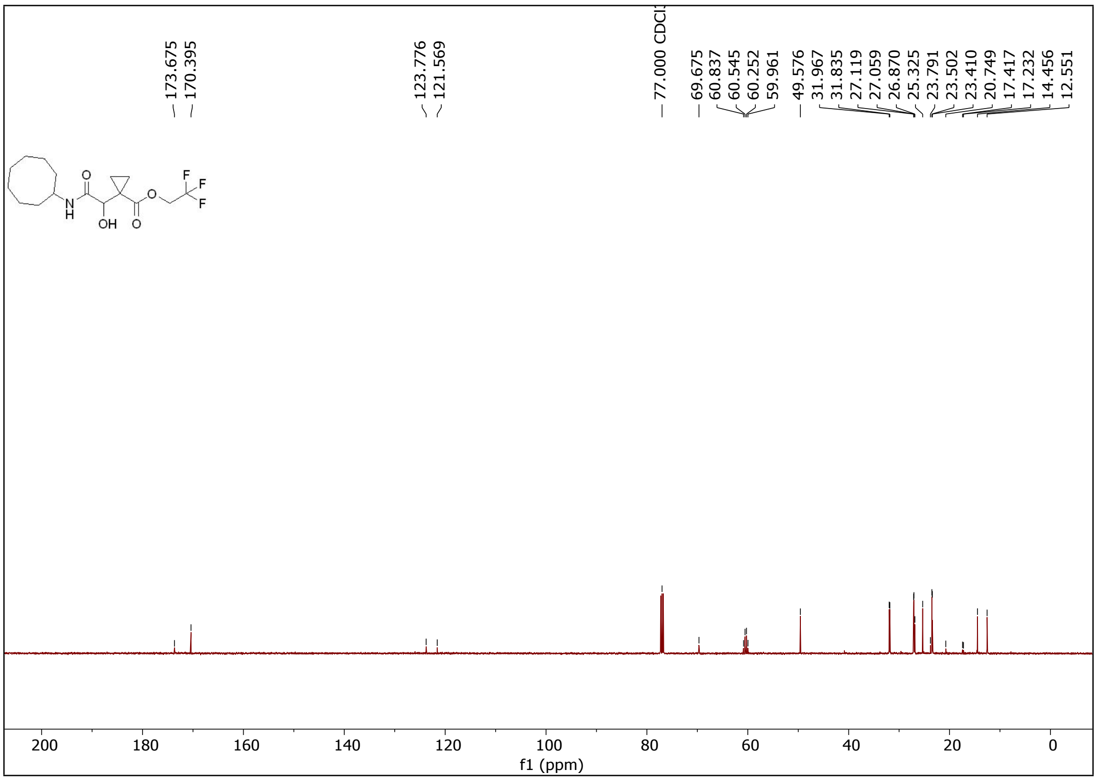

^{1}H NMR spectrum (500 MHz, $CDCl_3$) of compound 17j

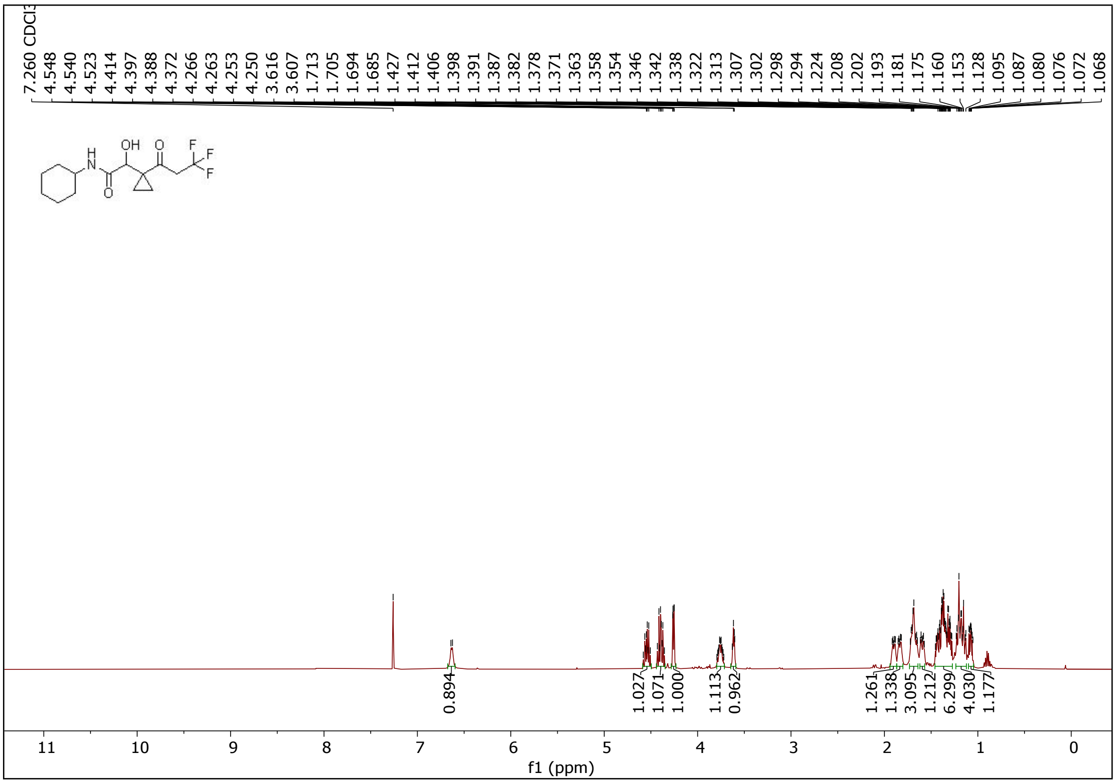

^{13}C NMR spectrum (125 MHz, $CDCl_3$) of compound 17j

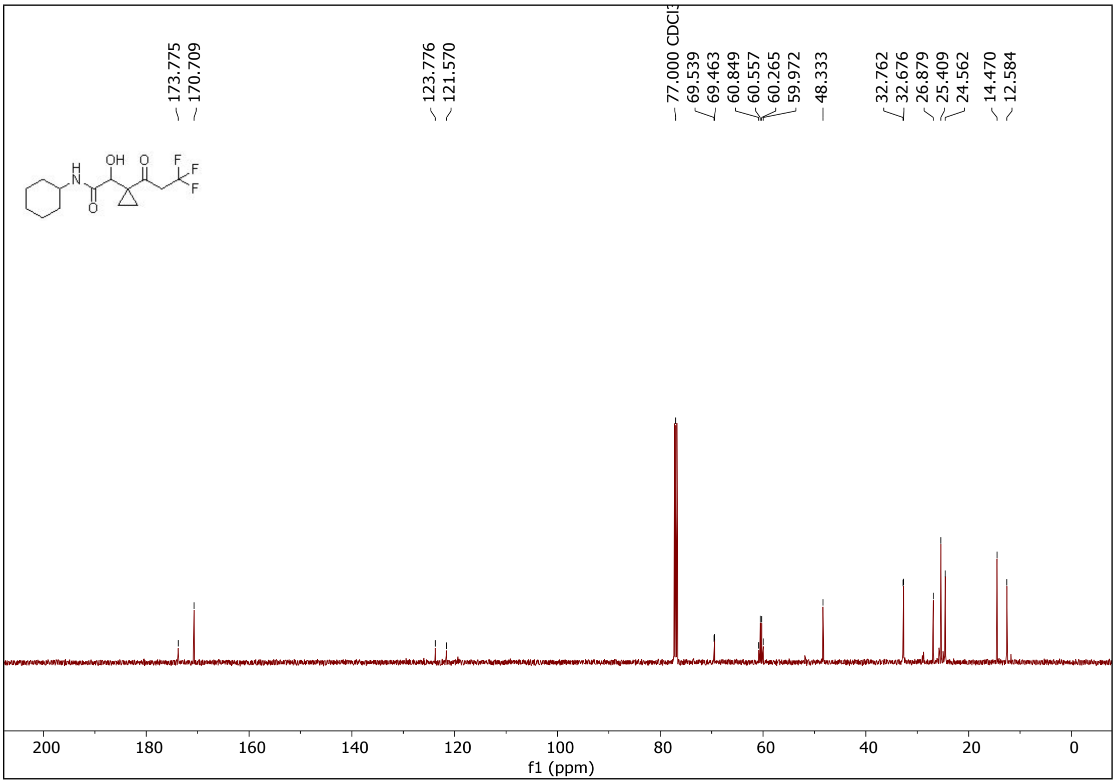

^{1}H NMR spectrum (500 MHz, $CDCl_3$) of compound 17k

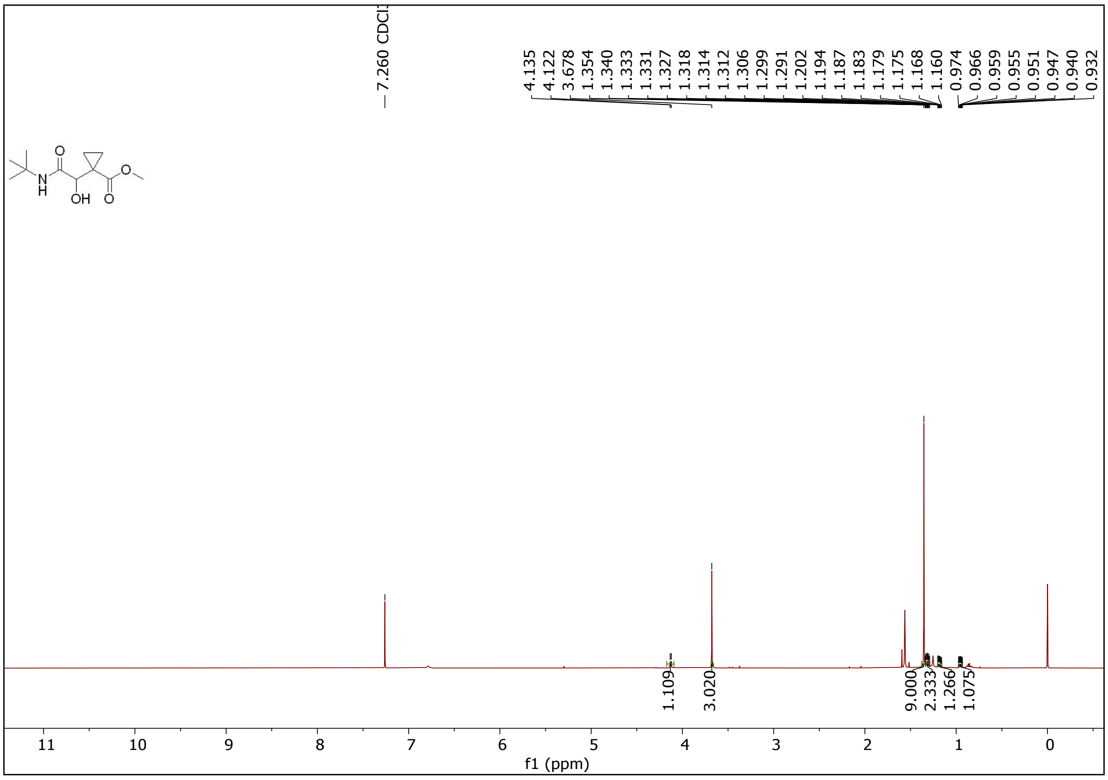

^{13}C NMR spectrum (125 MHz, $CDCl_3$) of compound 17k

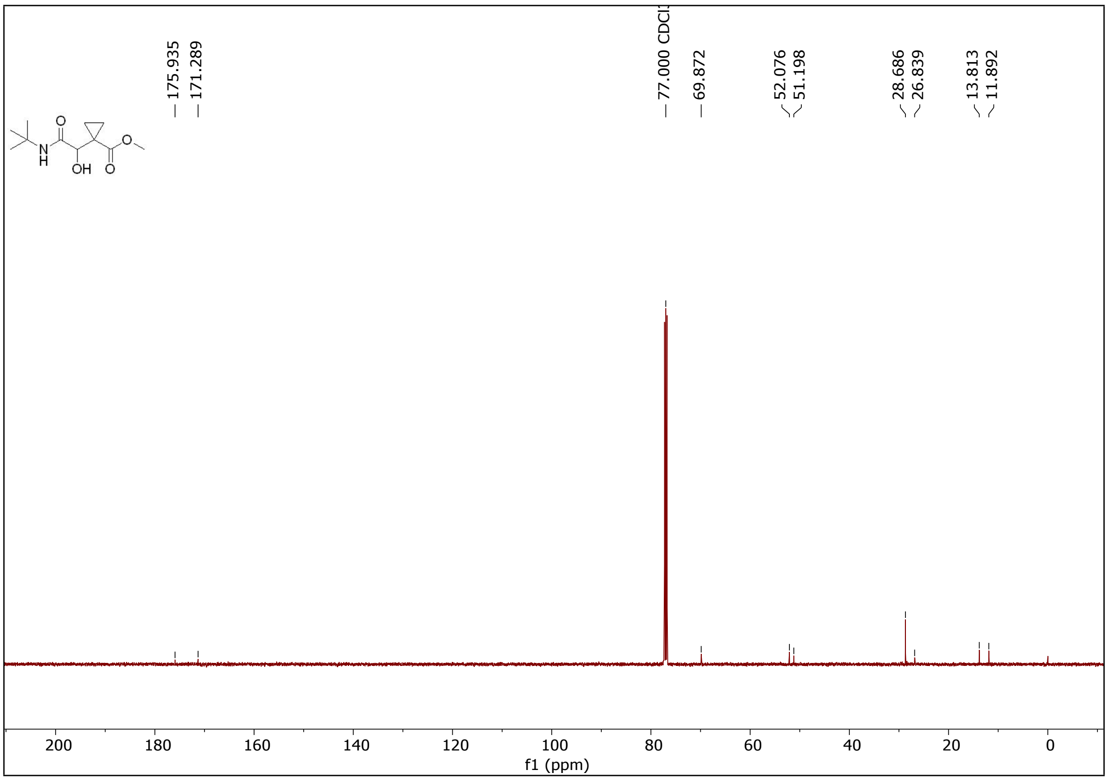

^{1}H NMR spectrum (500 MHz, $CDCl_3$) of compound 18

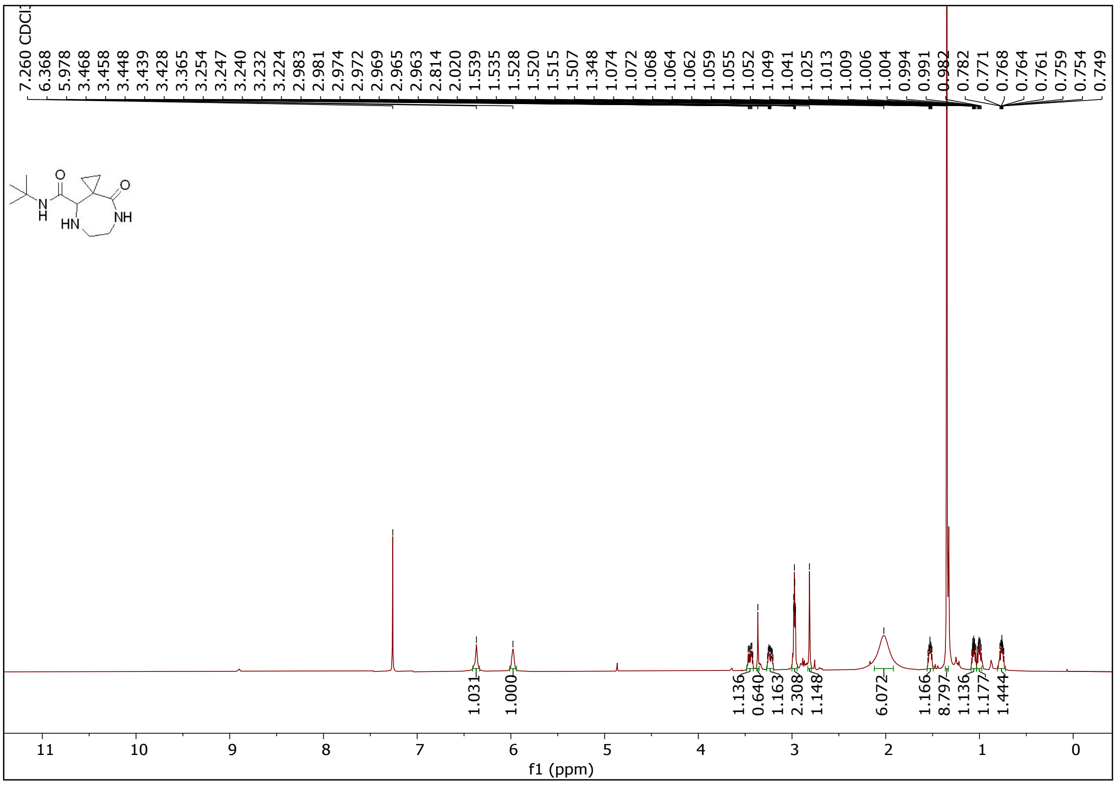

^{13}C NMR spectrum (125 MHz, $CDCl_3$, DMSO) of compound 18

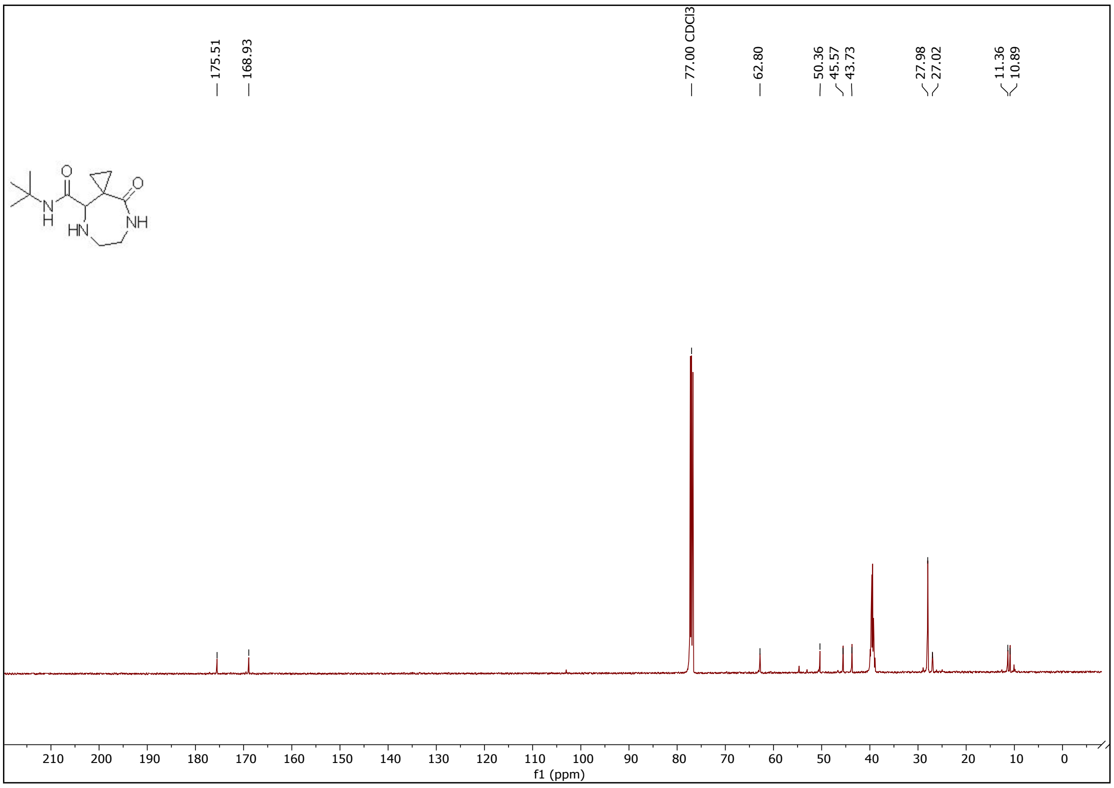

^{1}H NMR spectrum (500 MHz, $CDCl_3$) of compound 16o

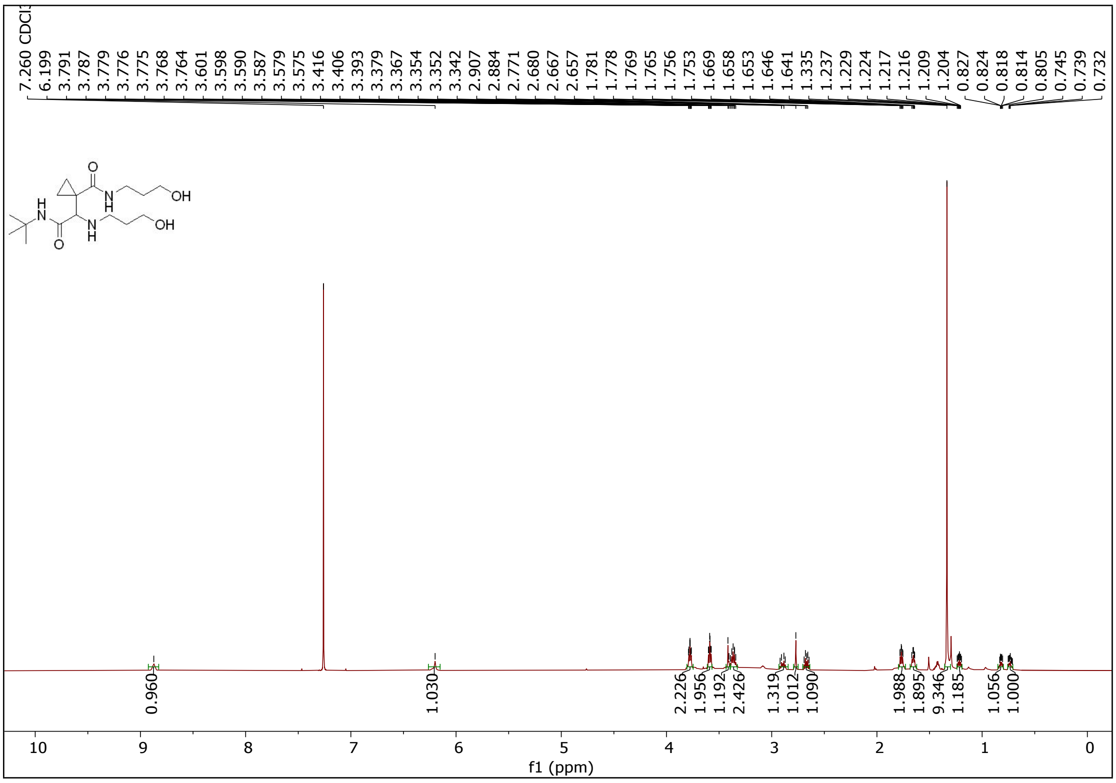

^{13}C NMR spectrum (125 MHz, $CDCl_3$) of compound 16o

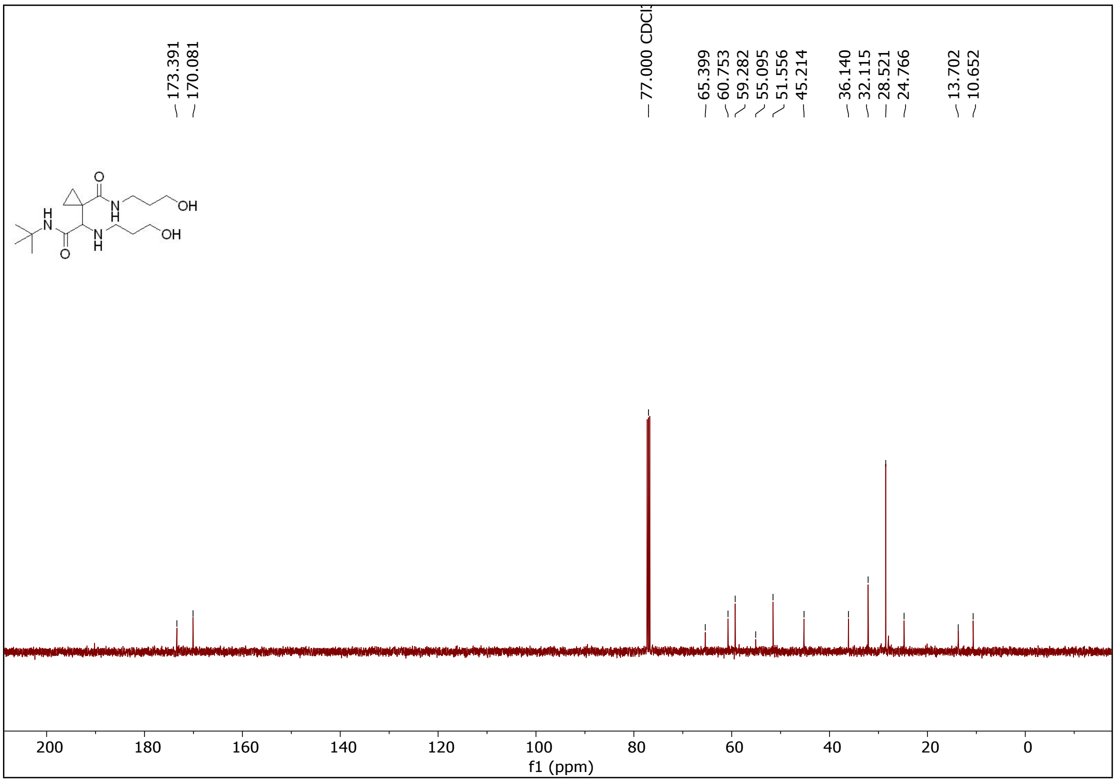

^{1}H NMR spectrum (500 MHz, $CDCl_3$) of compound 19a

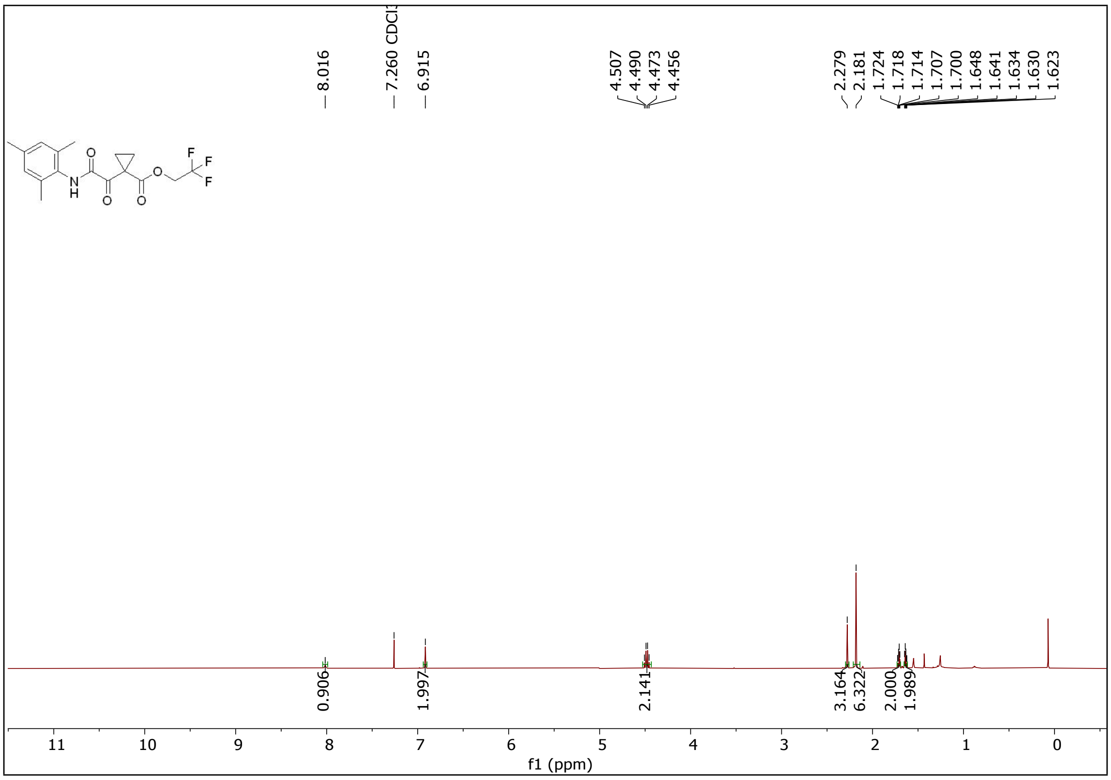

^{13}C NMR spectrum (125 MHz, $CDCl_3$) of compound 19a

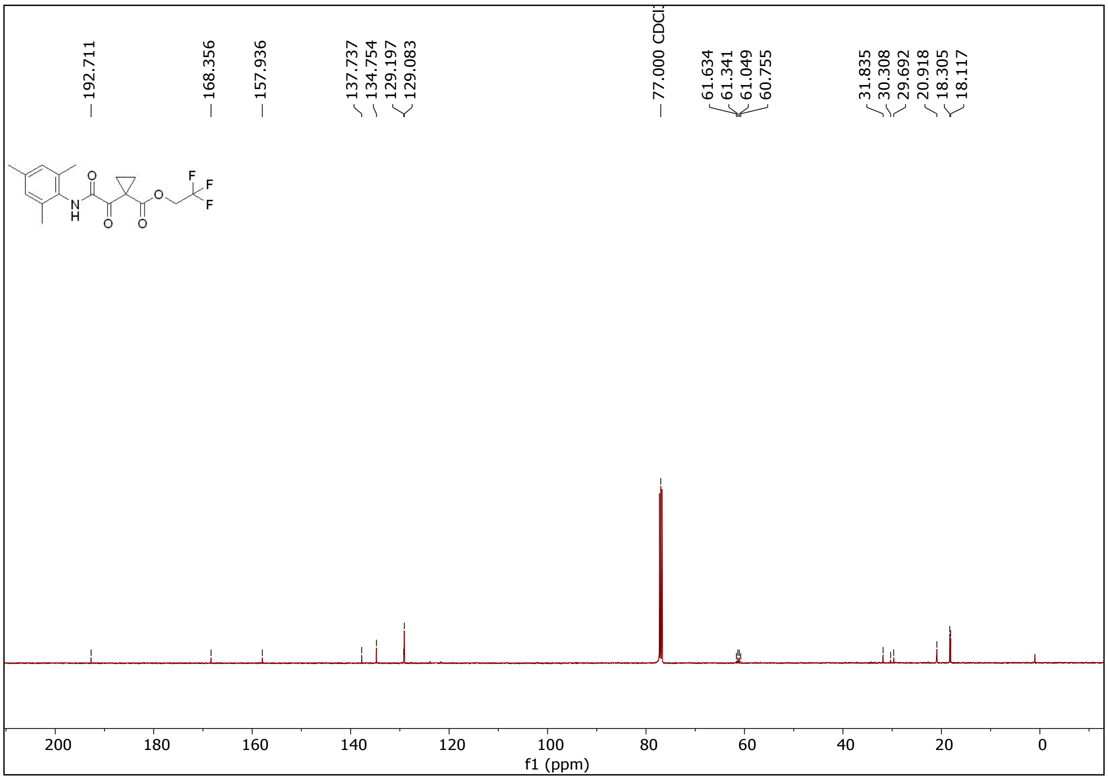

^{1}H NMR spectrum (500 MHz, $CDCl_3$) of compound 19b

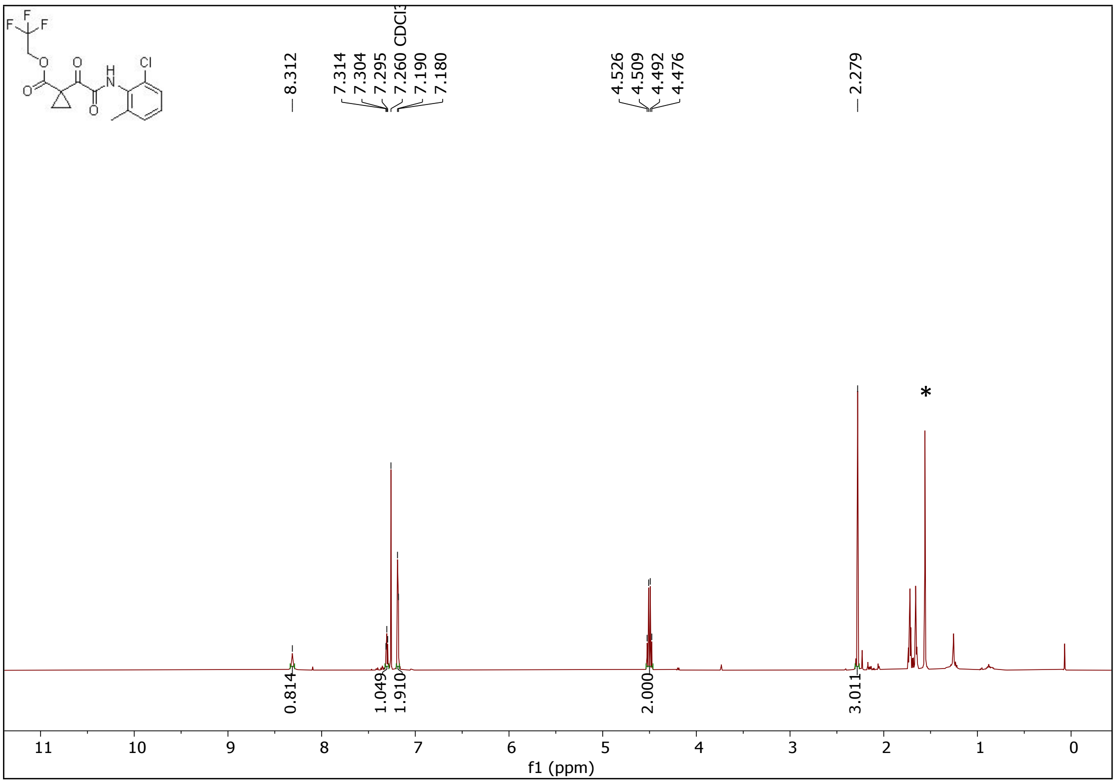

*: water

^{13}C NMR spectrum (125 MHz, $CDCl_3$) of compound 19b

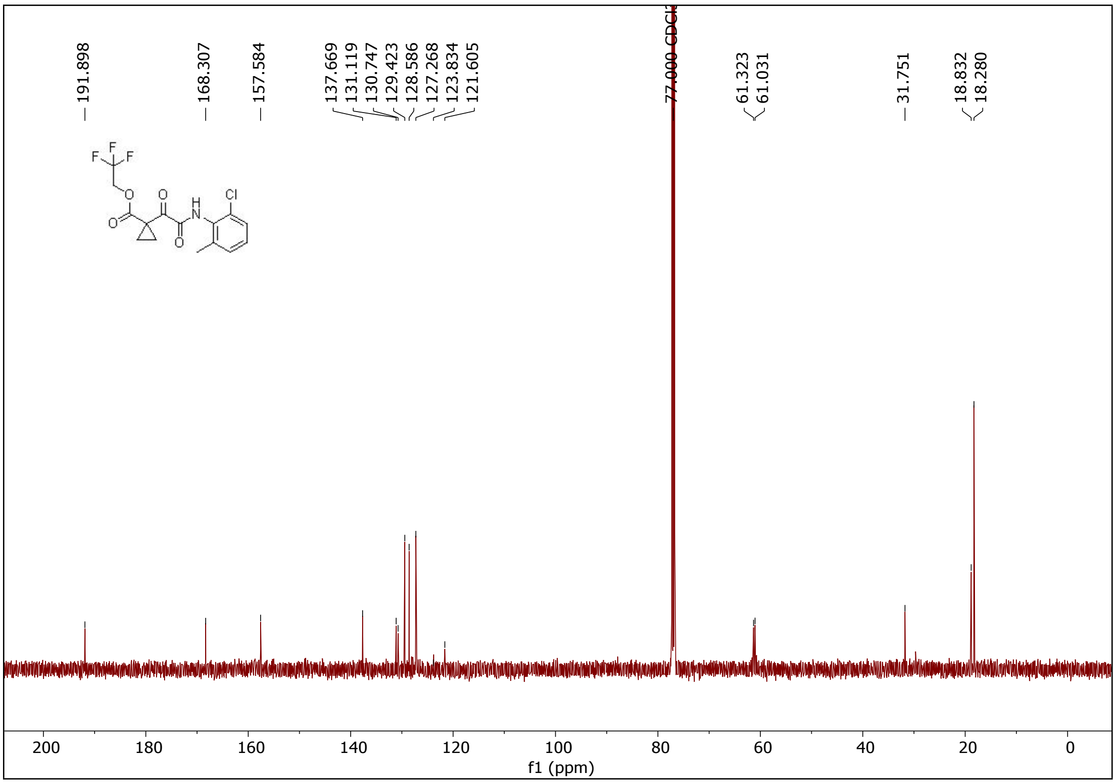

^{1}H NMR spectrum (500 MHz, $CDCl_3$) of compound 19c

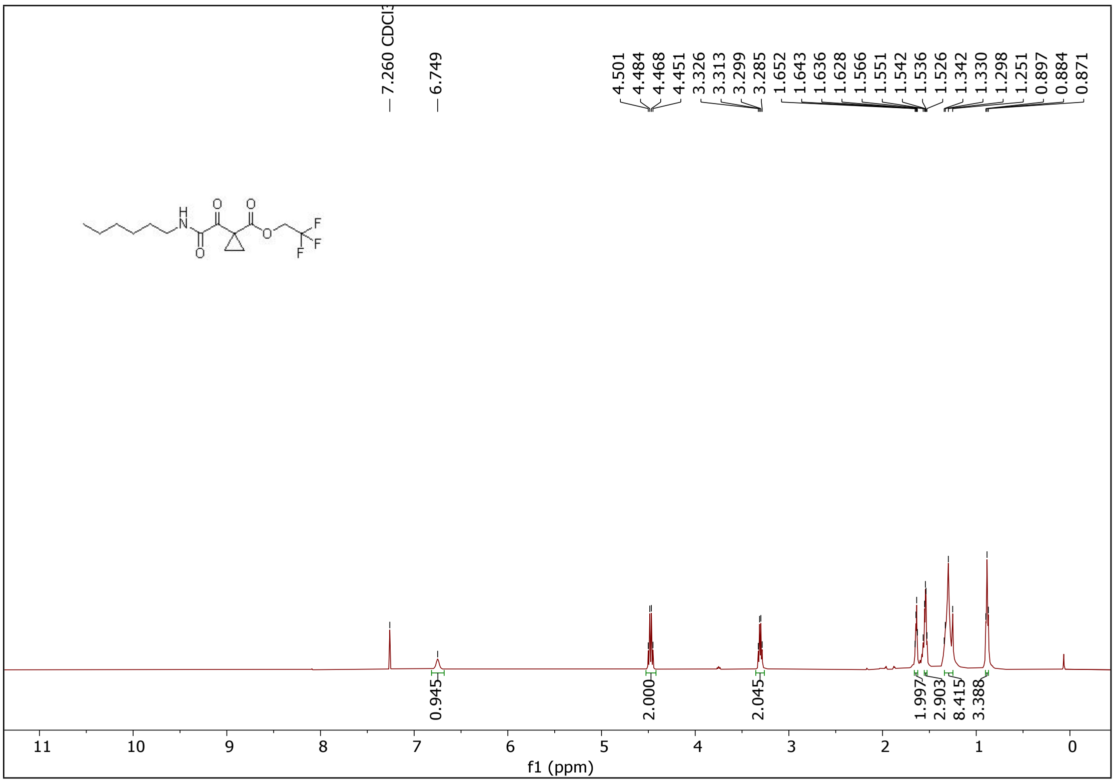

^{13}C NMR spectrum (125 MHz, $CDCl_3$) of compound 19c

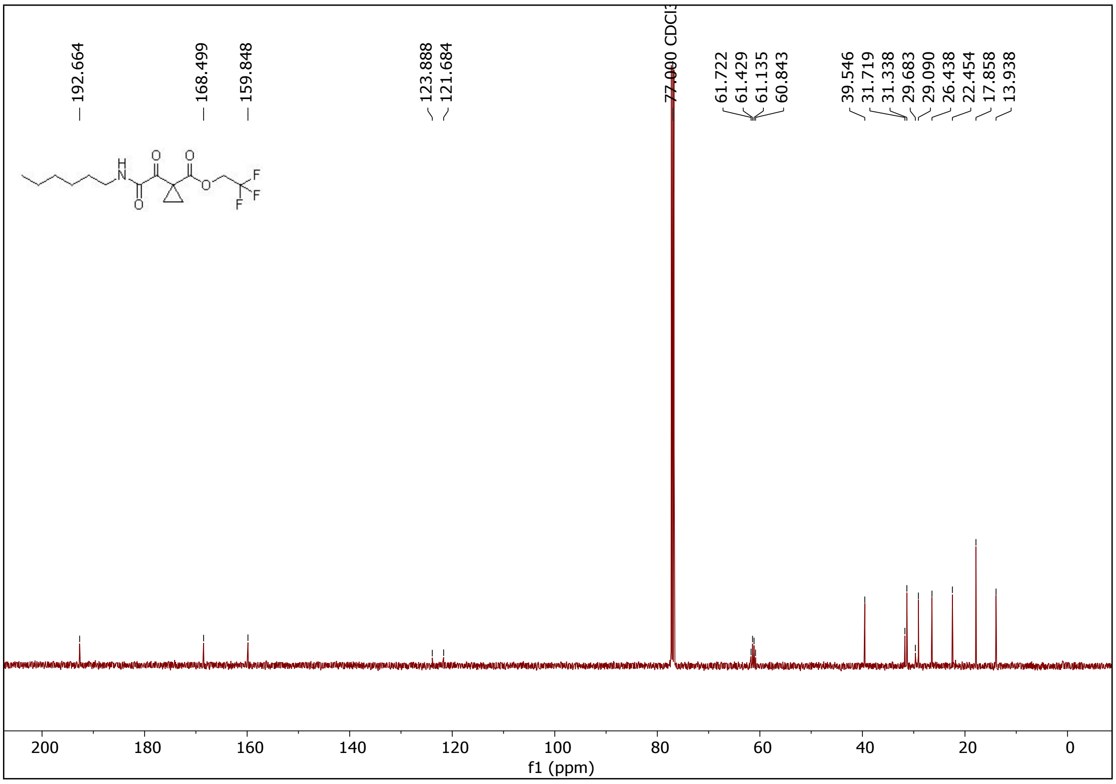

^{1}H NMR spectrum (500 MHz, $CDCl_3$) of compound 19d

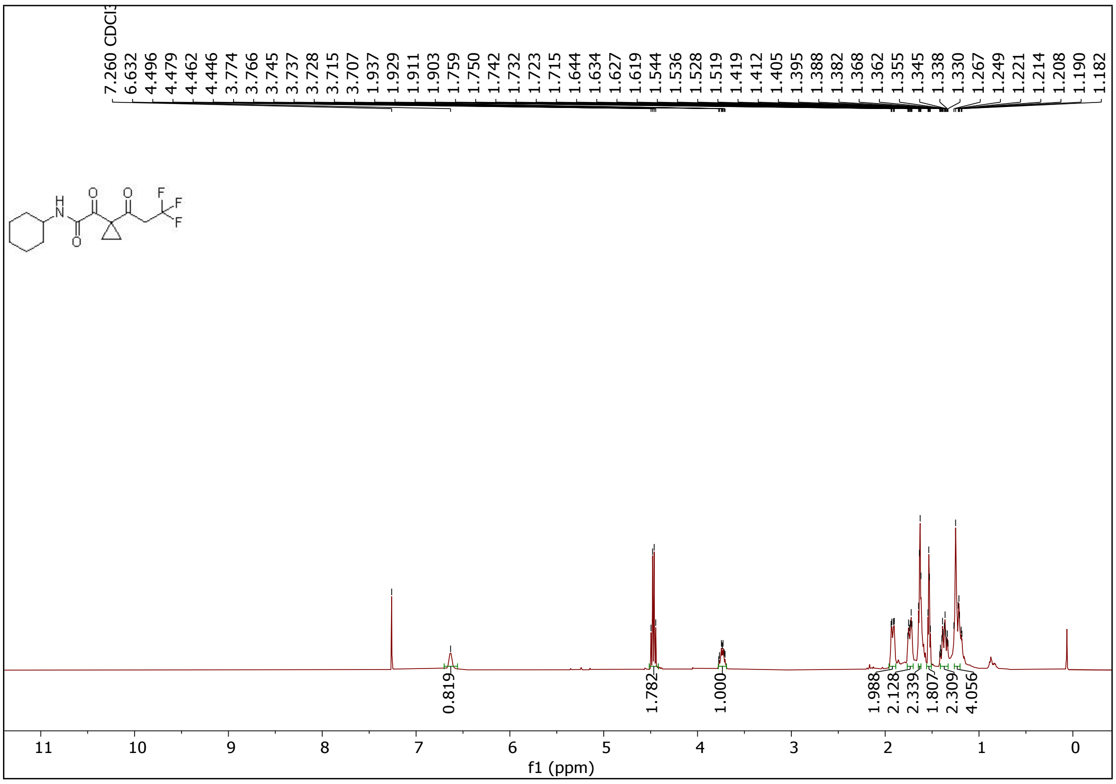

^{13}C NMR spectrum (125 MHz, $CDCl_3$) of compound 19d

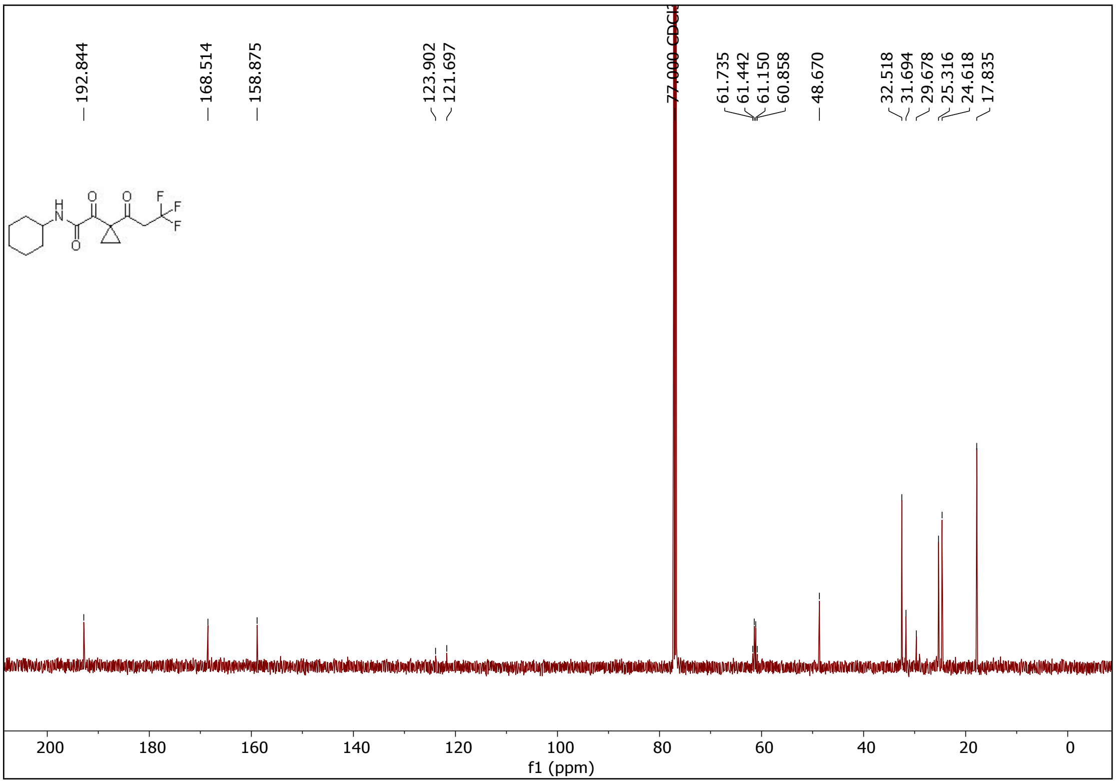

^{1}H NMR spectrum (500 MHz, $CDCl_3$) of compound 21a

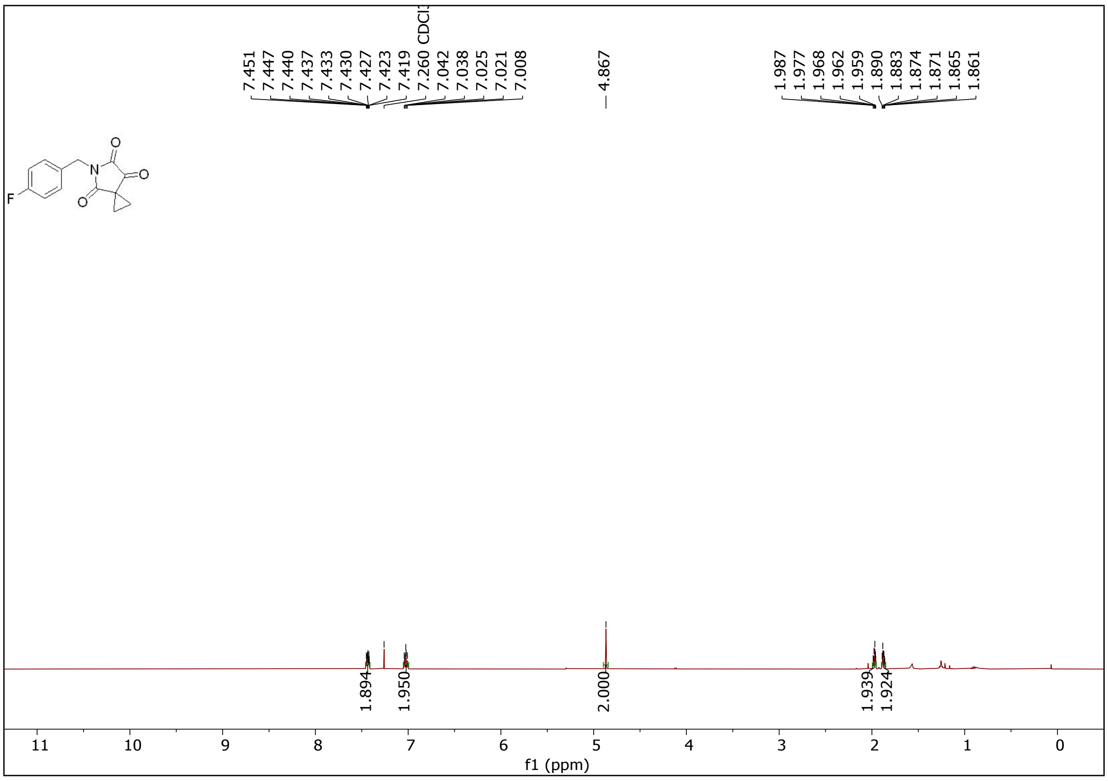

^{13}C NMR spectrum (125 MHz, $CDCl_3$) of compound 21a

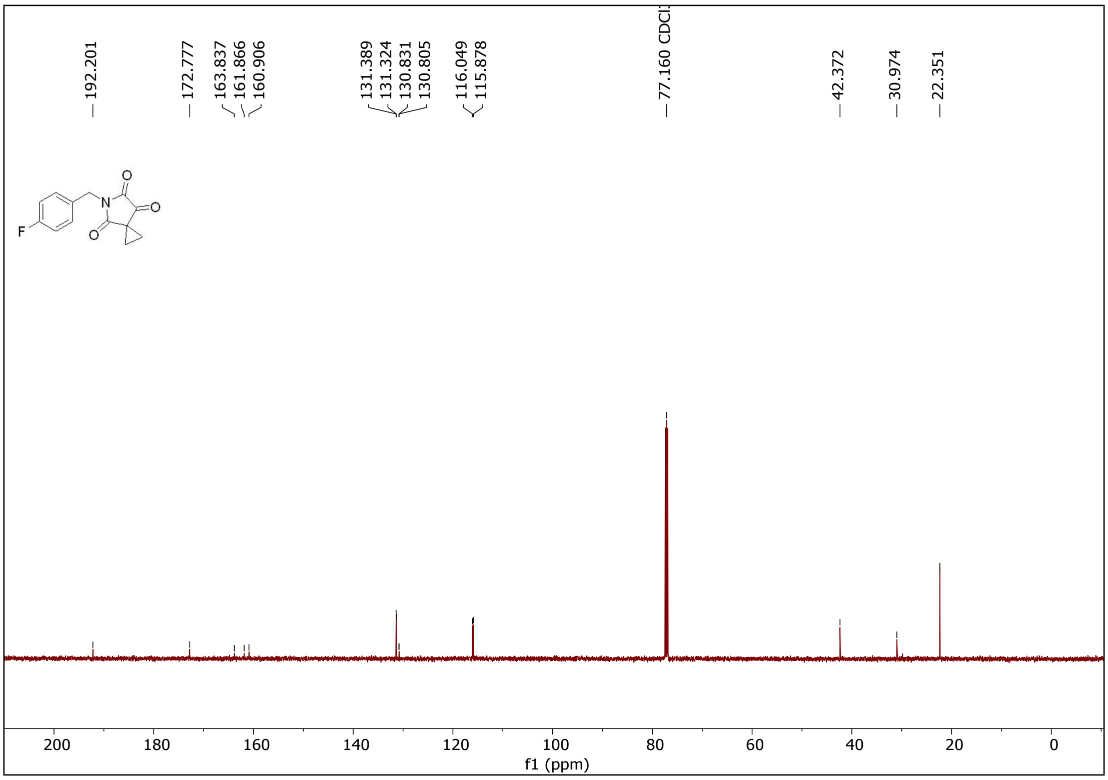

^{1}H NMR spectrum (500 MHz, $CDCl_3$) of compound 21b

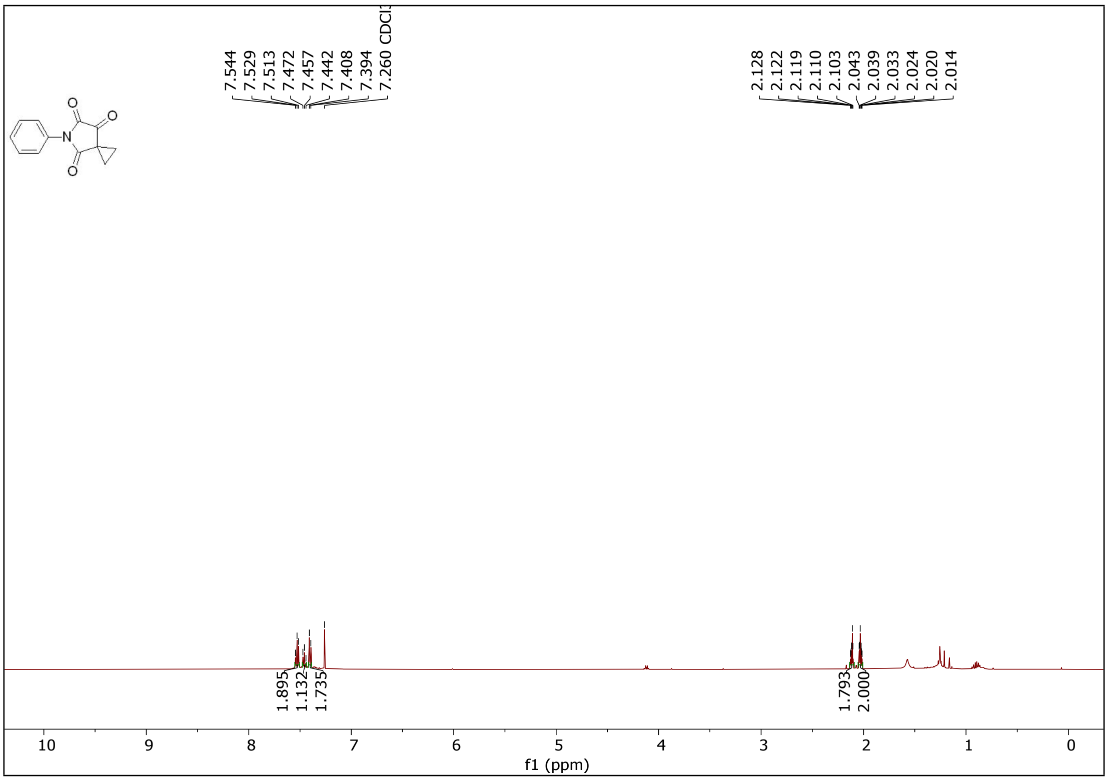

^{13}C NMR spectrum (125 MHz, $CDCl_3$) of compound 21b

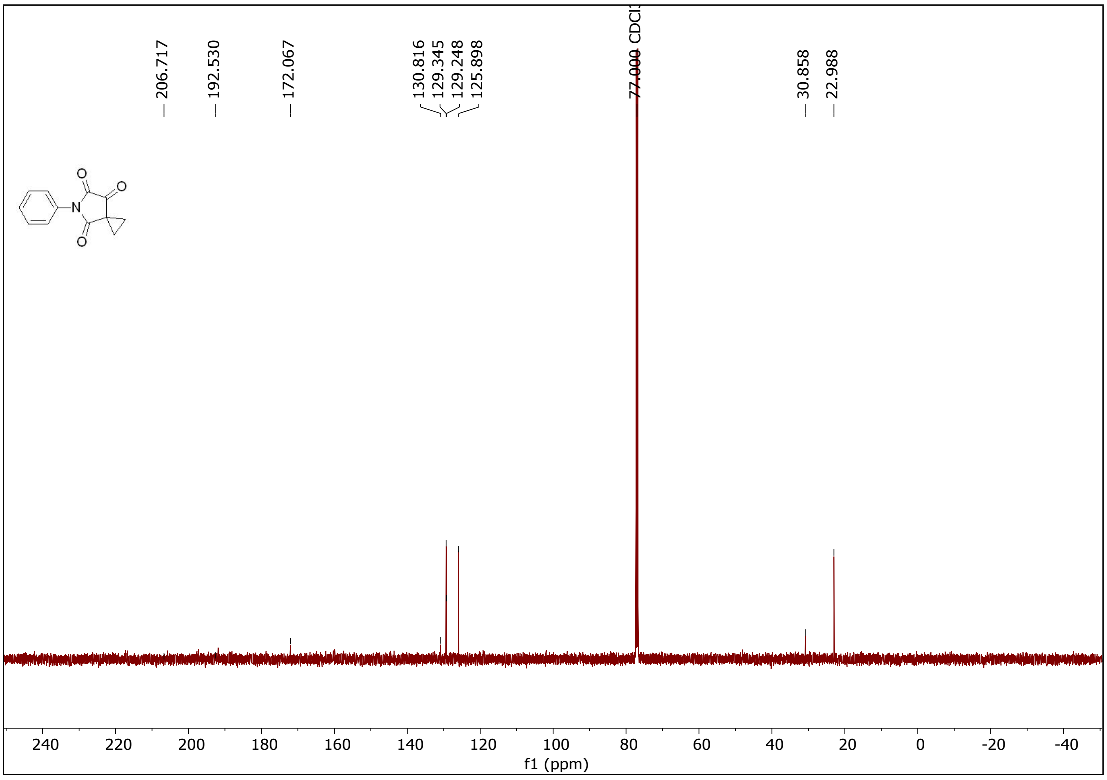

^{1}H NMR spectrum (500 MHz, $CDCl_3$) of compound 21c

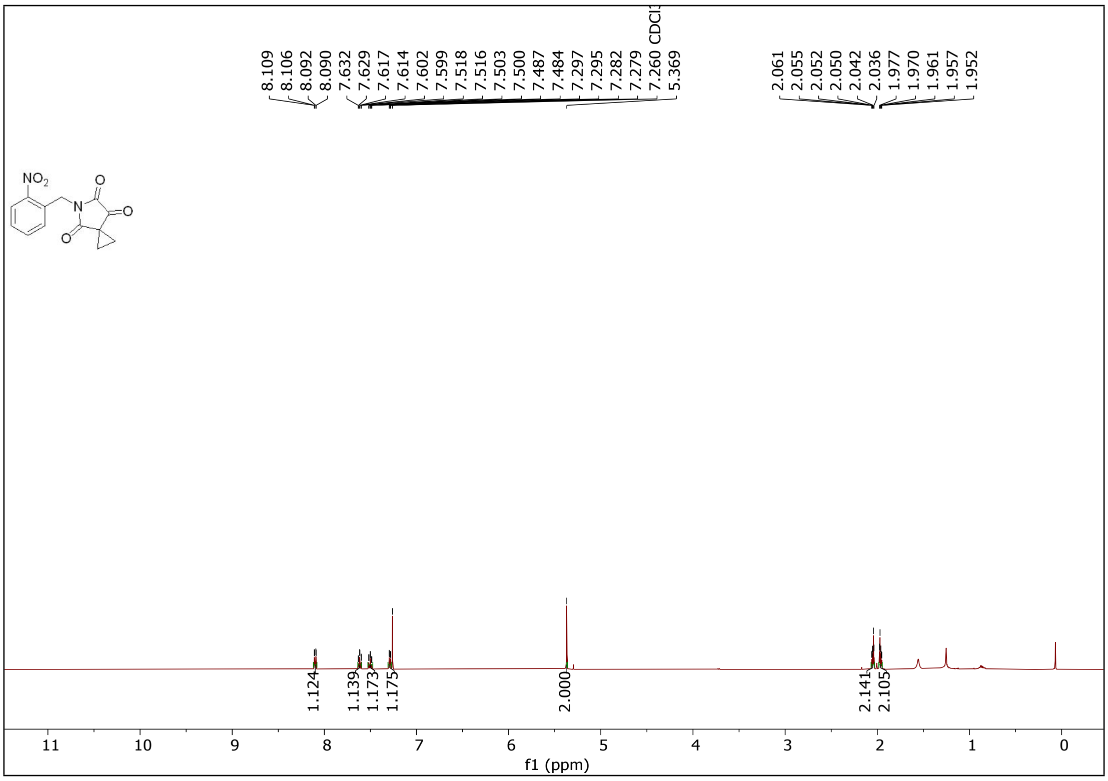

^{13}C NMR spectrum (125 MHz, $CDCl_3$) of compound 21c

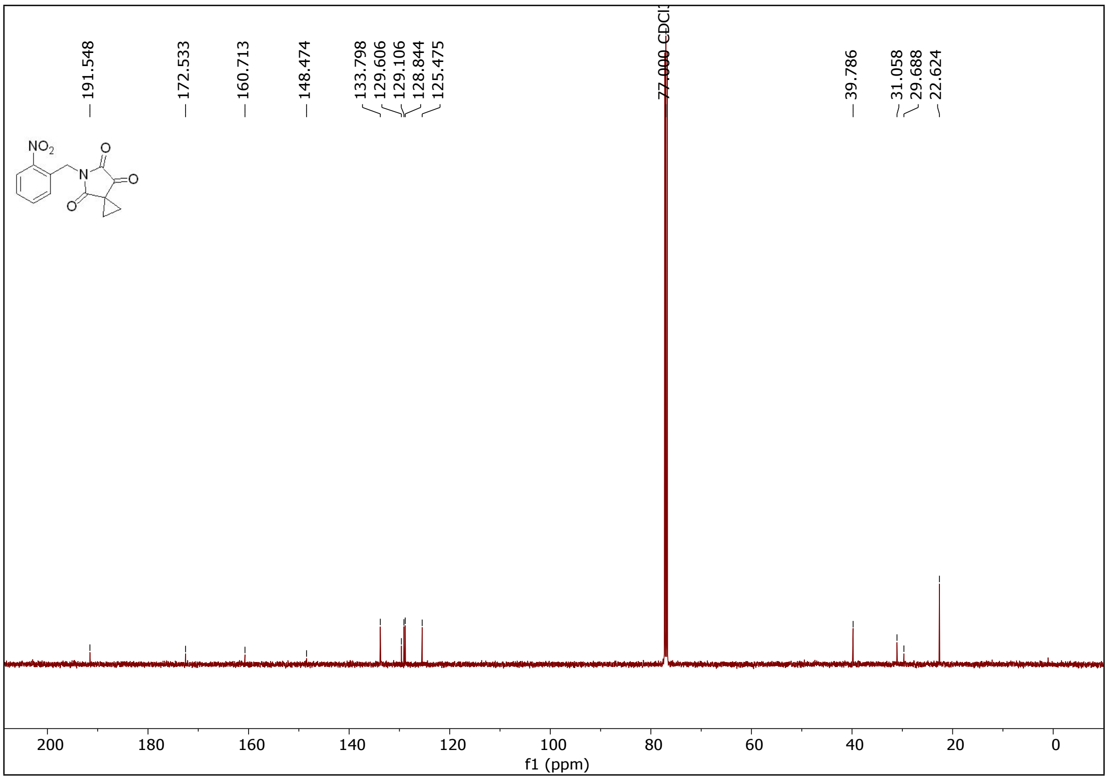

6. Single crystal x-ray structure determination

Single crystal structure of compound **16n**

A specimen of $C_{24}H_{31}N_3O_2$ was used for the X-ray crystallographic analysis. The X-ray intensity data were measured (λ = 1.54178 Å). The total exposure time was 22.90 hours. The frames were integrated with the Bruker SAINT software package using a narrow-frame algorithm. The integration of the data using an orthorhombic unit cell yielded a total of 29009 reflections to a maximum θ angle of 65.33° (0.85 Å resolution), of which 3783 were independent (average redundancy 7.668, completeness = 99.8%, R_{int} = 14.85%, R_{sig} = 6.97%) and 2468 (65.24%) were greater than $2\sigma(F^2)$. The final cell constants of a = 18.8503(10) Å, b = 9.7657(5) Å, c = 24.0318(10) Å, volume = 4423.9(4) $Å^3$, are based upon the refinement of the XYZ-centroids of 2507 reflections above 20 σ(I) with 7.357° < 2θ < 128.4°. Data were corrected for absorption effects using the Multi-Scan method (SADABS). The ratio of minimum to maximum apparent transmission was 0.770. The calculated minimum and maximum transmission coefficients (based on crystal size) are 0.9760 and 0.9940.

The structure was solved and refined using the Bruker SHELXTL Software Package,[2] using the space group P b c a, with Z = 8 for the formula unit, $C_{24}H_{31}N_3O_2$. The final anisotropic full-matrix least-squares refinement on F^2 with 265 variables converged at R1 = 6.13%, for the observed data and wR2 = 17.06% for all data. The goodness-of-fit was 1.021. The largest peak in the final difference electron density synthesis was 0.426 $e^-/Å^3$ and the largest hole was -0.500 $e^-/Å^3$ with an RMS deviation of 0.044 $e^-/Å^3$. On the basis of the final model, the calculated density was 1.182 g/cm^3 and F(000), 1696 e^-. CCDC Number: 2412460.

Table 1. Crystal data and structure refinement for **16n**

**Identification code**	PA97	
**Chemical formula**	$C_{24}H_{31}N_3O_2$	
**Formula weight**	393.52 g/mol	
**Temperature**	220(2) K	
**Wavelength**	1.54178 Å	
**Crystal size**	0.010 x 0.020 x 0.040 mm	
**Crystal system**	orthorhombic	
**Space group**	P b c a	
**Unit cell dimensions**	a = 18.8503(10) Å	α = 90°
	b = 9.7657(5) Å	β = 90°
	c = 24.0318(10) Å	γ = 90°
**Volume**	4423.9(4) $Å^3$	
**Z**	8	
**Density (calculated)**	1.182 g/cm^3	
**Absorption coefficient**	0.598 mm^{-1}	
**F(000)**	1696	

**Theta range for data collection**	3.68 to 65.33°
**Index ranges**	-22<=h<=22, -11<=k<=11, -28<=l<=27
**Reflections collected**	29009
**Independent reflections**	3783 [R(int) = 0.1485]
**Coverage of independent reflections**	99.8%
**Absorption correction**	Multi-Scan
**Max. and min. transmission**	0.9940 and 0.9760
**Structure solution technique**	Direct methods
**Refinement method**	Full-matrix least-squares on F^2
**Function minimized**	Σ w(F_o^2 - F_c^2)^2
**Data / restraints / parameters**	3783 / 0 / 265
**Goodness-of-fit on F²**	1.021
**Final R indices**	2468 data; I>2σ(I) R1 = 0.0613, wR2 = 0.1472
	all data R1 = 0.1001, wR2 = 0.1706
**Weighting scheme**	w=1/[σ^2(F_o^2)+(0.0724P)^2+1.6821P] where P=(F_o^2+2F_c^2)/3
**Largest diff. peak and hole**	0.426 and -0.500 eÅ^{-3}
**R.M.S. deviation from mean**	0.044 eÅ^{-3}

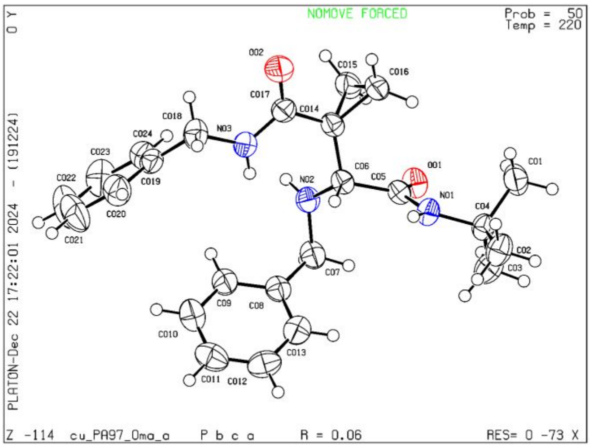

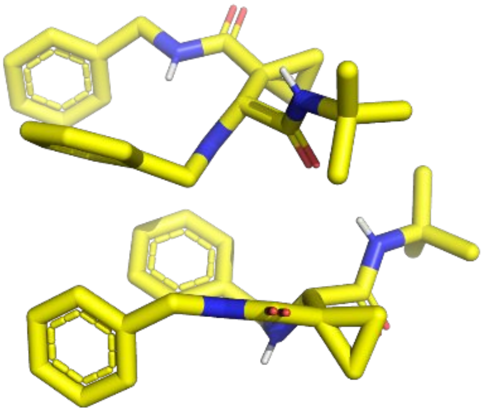

Single crystal structure of compound **17f**

A specimen of $C_{17}H_{20}F_3NO_4$ was used for the X-ray crystallographic analysis. The X-ray intensity data were measured (λ = 1.54178 Å). The total exposure time was 4.45 hours. The frames were integrated with the Bruker SAINT software package using a narrow-frame algorithm. The integration of the data using a monoclinic unit cell yielded a total of 12732 reflections to a maximum θ angle of 59.01° (0.90 Å resolution), of which 2869 were independent (average redundancy 4.438, completeness = 99.7%, R_{int} = 2.48%, R_{sig} = 2.23%) and 2435 (84.87%) were greater than $2\sigma(F^2)$. The final cell constants of a = 26.0028(5) Å, b = 16.2225(3) Å, c = 9.4671(2) Å, β = 93.1310(10)°, volume = 3987.55(14) $Å^3$, are based upon the refinement of the XYZ-centroids of 7644 reflections above 20 σ(I) with 6.808° < 2θ < 118.0°. Data were corrected for absorption effects using the Multi-Scan method (SADABS). The ratio of minimum to maximum apparent transmission was 0.826. The calculated minimum and maximum transmission coefficients (based on crystal size) are 0.9650 and 0.9910.

The structure was determined using OLEX2[3] suite, solved using SHELXT[4] and refined with SHELXL,[2] using the space group C 1 2/c 1, with Z = 8 for the formula unit, $C_{17}H_{20}F_3NO_4$. The final anisotropic full-matrix least-squares refinement converged at R1 = 6.50%, for the observed data and wR2 = 18.76% for all data. The goodness-of-fit was 1.078. The largest peak in the final difference electron density synthesis was 0.59 $e^-/Å^3$ and the largest hole was -0.55 $e^-/Å^3$. On the basis of the final model, the calculated density was 1.197 g/cm^3 and F(000), 1504 e^-. CCDC Number: 2412459.

Table 2. Crystal data and structure refinement for **17f.**

**Identification code**	PA53	
**Chemical formula**	$C_{17}H_{20}F_3NO_4$	
**Formula weight**	359.34 g/mol	
**Temperature**	200(2) K	
**Wavelength**	1.54178 Å	
**Crystal size**	0.010 x 0.020 x 0.040 mm	
**Crystal system**	monoclinic	
**Space group**	C 1 2/c 1	
**Unit cell dimensions**	a = 26.0028(5) Å	α = 90°
	b = 16.2225(3) Å	β = 93.1310(10)°
	c = 9.4671(2) Å	γ = 90°
**Volume**	3987.55(14) $Å^3$	
**Z**	8	
**Density (calculated)**	1.197 g/cm^3	
**Absorption coefficient**	0.888 mm^{-1}	
**F(000)**	1504	
**Theta range for data collection**	3.21 to 59.01°	
**Index ranges**	-28<=h<=28, -18<=k<=17, -10<=l<=10	

**Reflections collected**	12732
**Independent reflections**	2869 [R(int) = 0.0248]
**Coverage of independent reflections**	99.7%
**Absorption correction**	Multi-Scan
**Max. and min. transmission**	0.9910 and 0.9650
**Structure solution technique**	direct methods
**Data / restraints / parameters**	2869 / 0 / 230
**Goodness-of-fit on F²**	1.078
**Final R indices**	2435 data; I>2σ(I) R1 = 0.0650, wR2 = 0.1775
	all data R1 = 0.0734, wR2 = 0.1876
**Largest diff. peak and hole**	0.59 and -0.55 eÅ^{-3}

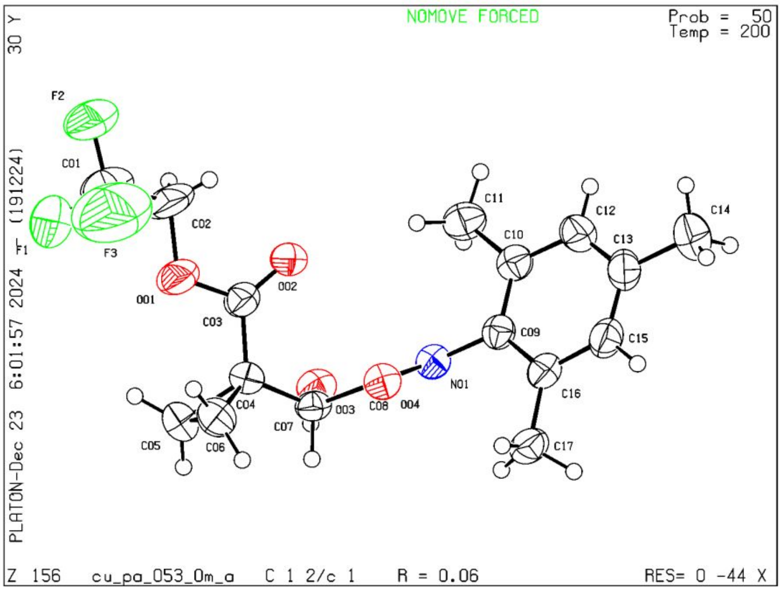

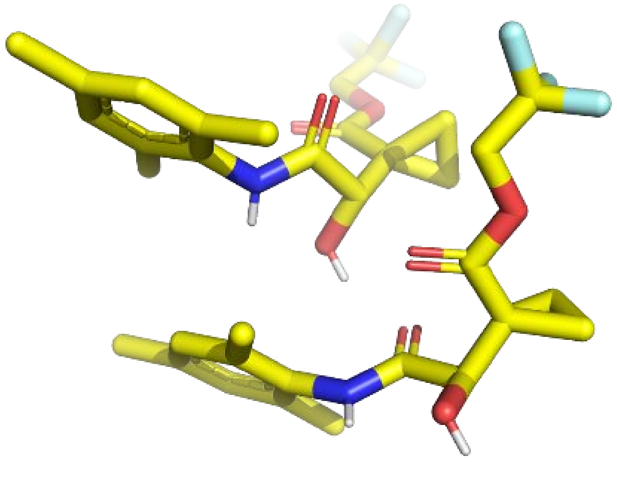

Single crystal structure of compound **21c**

A specimen of $C_{13}H_{10}N_2O_5$ was used for the X-ray crystallographic analysis. The X-ray intensity data were measured (λ = 1.54178 Å). The total exposure time was 9.05 hours. The frames were integrated with the Bruker SAINT software package using a narrow-frame algorithm. The integration of the data using an orthorhombic unit cell yielded a total of 7824 reflections to a maximum θ angle of 65.07° (0.85 Å resolution), of which 1935 were independent (average redundancy 4.043, completeness = 100.0%, R_{int} = 2.52%, R_{sig} = 2.13%) and 1865 (96.38%) were greater than 2σ(F^2). The final cell constants of <u>a</u> = 10.7917(2) Å, <u>b</u> = 22.5937(5) Å, <u>c</u> = 5.21170(10) Å, volume = 1270.74(4) $Å^3$, are based upon the refinement of the XYZ-centroids of 5978 reflections above 20 σ(I) with 7.826° < 2θ < 130.0°. Data were corrected for absorption effects using the Multi-Scan method (SADABS). The ratio of minimum to maximum apparent transmission was 0.918. The calculated minimum and maximum transmission coefficients (based on crystal size) are 0.9630 and 0.9810.

The structure was solved and refined using the Bruker SHELXTL Software Package,[2] using the space group P n a 21, with Z = 4 for the formula unit, $C_{13}H_{10}N_2O_5$. The final anisotropic full-matrix least-squares refinement on F^2 with 181 variables converged at R1 = 3.35%, for the observed data and wR2 = 9.06% for all data. The goodness-of-fit was 1.040. The largest peak in the final difference electron density synthesis was 0.211 $e^-/Å^3$ and the largest hole was -0.197 $e^-/Å^3$ with an RMS deviation of 0.033 $e^-/Å^3$. On the basis of the final model, the calculated density was 1.433 g/cm^3 and F(000), 568 e^-. CCDC Number: 2412461.

Table 3. Crystal data and structure refinement for **21c.**

**Identification code**	PA146	
**Chemical formula**	$C_{13}H_{10}N_2O_5$	
**Formula weight**	274.23 g/mol	
**Temperature**	220(2) K	
**Wavelength**	1.54178 Å	
**Crystal size**	0.020 x 0.020 x 0.040 mm	
**Crystal system**	orthorhombic	
**Space group**	P n a 21	
**Unit cell dimensions**	a = 10.7917(2) Å	α = 90°
	b = 22.5937(5) Å	β = 90°
	c = 5.21170(10) Å	γ = 90°
**Volume**	1270.74(4) $Å^3$	
**Z**	4	
**Density (calculated)**	1.433 g/cm^3	
**Absorption coefficient**	0.957 mm^{-1}	
**F(000)**	568	
**Theta range for data collection**	3.91 to 65.07°	
**Index ranges**	-12<=h<=12, -26<=k<=26, -6<=l<=4	
**Reflections collected**	7824	
**Independent reflections**	1935 [R(int) = 0.0252]	

**Coverage of independent reflections**	100.0%	
**Absorption correction**	Multi-Scan	
**Max. and min. transmission**	0.9810 and 0.9630	
**Structure solution technique**	direct methods	
**Refinement method**	Full-matrix least-squares on F^2	
**Function minimized**	$\Sigma\ w(F_o^2 - F_c^2)^2$	
**Data / restraints / parameters**	1935 / 1 / 181	
**Goodness-of-fit on F²**	1.040	
**Final R indices**	1865 data; I>2σ(I)	R1 = 0.0335, wR2 = 0.0886
	all data	R1 = 0.0350, wR2 = 0.0906
**Weighting scheme**	$w=1/[\sigma^2(F_o^2)+(0.0520P)^2+0.2649P]$ where $P=(F_o^2+2F_c^2)/3$	
**Absolute structure parameter**	0.26(11)	
**Largest diff. peak and hole**	0.211 and -0.197 eÅ^{-3}	
**R.M.S. deviation from mean**	0.033 eÅ^{-3}	

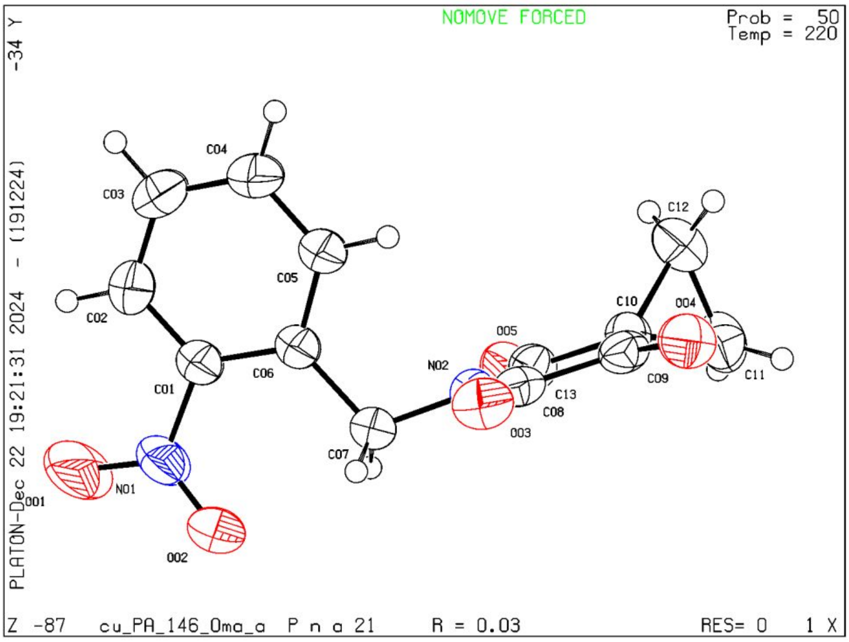

References

(1) Ashida, Y. Synthesis of Methyl 1-Formylcyclopropanecarboxylate Utilizing Ti-Claisen Condensation. *Organic Syntheses* **2016**, *93*, 286–305. https://doi.org/10.15227/orgsyn.093.0286.

(2) Sheldrick, G. M. SHELXT – Integrated Space-Group and Crystal-Structure Determination. *Acta Crystallogr A Found Adv* **2015**, *71* (1), 3–8. https://doi.org/10.1107/S2053273314026370.

(3) Dolomanov, O. V; Bourhis, L. J.; Gildea, R. J.; Howard, J. A. K.; Puschmann, H. OLEX2 : A Complete Structure Solution, Refinement and Analysis Program. *J Appl Crystallogr* **2009**, *42* (2), 339–341. https://doi.org/10.1107/S0021889808042726.

(4) Sheldrick, G. M. A Short History of SHELX. *Acta Crystallogr A* **2008**, *64* (1), 112–122. https://doi.org/10.1107/S0108767307043930.
